# Global, regional, and national burden and trends of stroke among youths and young adults aged 15–39 years from 1990 to 2021: findings from the Global Burden of Disease study 2021

**DOI:** 10.3389/fneur.2025.1535278

**Published:** 2025-03-07

**Authors:** Qian Yu, Yuan Tian, Nan Jiang, Furong Zhao, Shuang Wang, Miao Sun, Zhining Liu, Xin Liu

**Affiliations:** ^1^Huludao Central Hospital Teaching Base of Jinzhou Medical University, Liaoning, China; ^2^Liaoning Provincial Key Laboratory of Clinical Oncology Metabonomics, The First Affiliated Hospital of Jinzhou Medical University, Jinzhou, China; ^3^Fenyang College of Shanxi Medical University, Fenyang, China; ^4^Clinical Research Department, Dalian Boyuan Medical Technology Co., Ltd, Dalian, China; ^5^Department of Laboratory Medicine, General Hospital of Fushun Mining Bureau of Liaoning Health Industry Group, Fushun, China; ^6^Ultrasound Department, The First Affiliated Hospital of Jinzhou Medical University, Jinzhou, China

**Keywords:** stroke, risk factors, incidence, mortality, disability-adjusted life years

## Abstract

**Background:**

Stroke is a leading cause of disability and mortality worldwide, with rising incidence rates among youths and young adults aged 15–39 years. However, comprehensive assessments of stroke burden in this age group at global, regional, and national levels are limited. This study examines trends in stroke incidence, mortality, and disability-adjusted life years (DALYs) from 1990 to 2021 using data from the Global Burden of Disease (GBD) study.

**Methods:**

Data from the GBD study (1990–2021) were analyzed to assess the age-standardized incidence, mortality, and DALYs related to stroke in individuals aged 15–39 years. The relationship between stroke burden and the Socio-Demographic Index (SDI) was explored across 204 countries and 21 regions. Trends were analyzed using the estimated annual percentage change (EAPC) and average annual percentage change (AAPC).

**Results:**

This study reveals global, regional, and national trends in stroke burden among youths and young adults (15–39 years) from 1990 to 2021. In 2021, the global age-standardized stroke incidence was 757,234.61 cases, with 8.72 million DALYs and 122,742 stroke-related deaths. Although global incidence increased by 19.09%, age-standardized rates (ASRs) declined by 0.67% annually. DALYs and mortality rates also decreased globally. Notably, stroke burden increased in low and low-middle SDI regions. South Asia had the highest number of cases, while Oceania reported the highest mortality rate. These findings underscore regional disparities in stroke trends. Globally, metabolic risks (46.2%) and high systolic blood pressure (37.87%) are major contributors to stroke-related mortality.

## Introduction

Stroke is a major global health concern, contributing substantially to disability and premature mortality (1, 2). From 1990 to 2019, the age-standardized DALYs rate (ASDR) of stroke decreased by 36.0% globally, yet the absolute DALYs increased by 32.0% (3). While traditionally considered a disease of older populations, stroke among youths and young adults (15–39 years) has gained increasing attention due to its rising incidence, distinct etiologies, and significant socioeconomic burden (4, 5). Young stroke survivors often face long-term disabilities, reduced quality of life, and significant socioeconomic burdens, resulting in substantial societal and economic challenges for both the individuals and their communities (6).

Previous studies have reported regional disparities in stroke burden, with variations driven by factors such as lifestyle changes, healthcare access, and socioeconomic development (7). The Global Burden of Disease (GBD) study provides a valuable framework for assessing these trends by analyzing age-standardized prevalence, mortality, and disability-adjusted life years (DALYs) for stroke (8, 9, 10).Evidence shows regional variations and an increasing prevalence of stroke among young individuals, especially in low- and middle-SDI regions (11). However, the burden of stroke in these areas is aggravated by a lack of targeted interventions, limited healthcare access, and insufficient awareness of stroke risk factors. Understanding these trends is critical for developing effective public health policies and interventions tailored to youths and young adults at risk (12).

This study aims to analyze the global, regional, and national burden of stroke among youths and young adults aged 15–39 years from 1990 to 2021 using GBD data. We seek to identify patterns in incidence, mortality, and DALYs, explore associations with the Socio-Demographic Index, and evaluate temporal trends to inform future strategies for stroke prevention and management in this population.

## Methods

### Overview and data collection

This cross-sectional study was approved by Jinzhou Medical University. The university’s ethics committee waived the requirement for informed consent, as the study involved only data analysis with no personally identifiable information.

The data on youth and young adult stroke used in this study were derived from the Global Burden of Disease Study 2021 (GBD 2021), which provides comprehensive and up-to-date estimates for 369 diseases, injuries, and conditions across 204 countries and territories, as well as 88 risk factors (7, 13). The GBD 2021 classification system divides the world into 21 geographic regions based on epidemiological similarities and geographical proximity. This regional categorization enables a nuanced understanding of disease burden variations across different parts of the world, thereby facilitating the development of targeted public health policies and interventions (10, 14). The 21 regions considered are as follows: Andean Latin America, Australasia, the Caribbean, Central Asia, Central Europe, Central Latin America, Central Sub-Saharan Africa, East Asia, Eastern Europe, Eastern Sub-Saharan Africa, High-Income Asia Pacific, High-Income North America, North Africa and the Middle East, Oceania, South Asia, Southeast Asia, Southern Latin America, Southern Sub-Saharan Africa, Tropical Latin America, Western Europe, and Western Sub-Saharan Africa. This classification has consistently been utilized in previous GBD iterations and has proven effective in analyzing and comparing health metrics across different geographic and epidemiological regions (7, 10, 14).

For this study, we specifically extracted data on young stroke, focusing on metrics such as incidence, mortality, and disability-adjusted life years (DALYs) to provide a detailed analysis of the burden of stroke among youths and young adults. All data used in this study is publicly accessible through the Global Health Data Exchange platform.^1^ The study was conducted in compliance with the Strengthening the Reporting of Observational Studies in Epidemiology (STROBE) guidelines (15).

### Sociodemographic index

The Sociodemographic index is a composite measure of a country’s or region’s level of development, incorporating indicators such as fertility rates, educational attainment, and per capita income. SDI values range from 0 to 1, with higher scores reflecting greater socioeconomic development. Previous studies have demonstrated an association between SDI and both disease incidence and mortality rates (16). In this study, we classified countries and regions into five SDI categories (low, low-medium, medium, medium-high, and high) to investigate the relationship between socioeconomic development and the burden of stroke in youths and young adults aged 15–39 years from 1990 to 2021.

### Statistical analysis

To assess the burden of stroke in youths and young adults, the primary indicators analyzed were incidence, mortality, and disability-adjusted life years (DALYs). Trends were interpreted based on the estimated annual percent change (EAPC) and its 95% confidence interval (CI): a negative upper limit of the EAPC indicated a decreasing trend, while a positive lower limit suggested an increasing trend (17, 18, 19).

In this study, ASRs for stroke incidence, mortality, and DALYs were calculated using direct standardization, with the GBD 2021 world standard population as the weighting factor. ASRs were reported per 100,000 population, accompanied by 95% uncertainty intervals (UI). To analyze trends in age-specific stroke incidence, mortality, and DALYs at global, regional, and national levels, GBDR analysis was used (20). JD_GBDR software was applied to assess trends from 1990 to 2021. The analysis calculated the average annual percentage change (AAPC) and its 95% CI. Trends were classified as upward (AAPC >0), downward (AAPC <0), or stable (95% CI includes 0). UIs, which differ from CIs by accounting for uncertainties from multiple sources such as sampling error, model estimation, and specification, were also computed (21).

In this study, the R software package (version 4.2.3) and JD_GBDR (V2.22, Jingding Medical Technology Co., Ltd.) were used for the drawing of the figures.

## Results

### Global trends in stroke incidence, mortality and DALY rate

In 2021, the global age-standardized incidence of stroke was 757,234.61 cases (95% UI: 632,119.62–924,338.12) (Table 1). The age-standardized disability-adjusted life years (DALYs) due to stroke totaled 8,718,567.32 (95% UI: 7,951,154.52–9,472,143.29) (Supplementary Table S1), while age-standardized stroke-related deaths were 122,742.66 (95% UI: 113,243.10–133,292.05) (Table 2). From 1990 to 2021, the global age-standardized incidence of stroke increased by 19.09% (95% UI: 13.56–24.02%). Despite this rise, the incidence rate declined from 29.01 (95% UI: 24.22–35.16) in 1990 to 25.45 (95% UI: 21.25–31.07) in 2021, with an estimated annual percentage change (EAPC) of −0.67 (95% CI: −0.76, −0.58) (Table 1). Over the same period, the age-standardized DALY burden decreased by 5.47%, and age-standardized mortality fell by 8.16%. Additionally, age-standardized mortality rates showed a significant reduction (AAPC = −0.065; 95% CI: −0.068, −0.063), as did age-standardized DALY rates (AAPC = −4.176; 95% CI: −4.301, −4.052) (Table 2; Supplementary Table S1).

**Table 1:** Age-standardized incidence and AAPC of stroke in individuals aged 15–39 years at global and regional levels, 1990–2021.

Location	Rate per 100,000 (95% UI)						
	1990		2021		1990–2021		
	Incident cases	Incidence rate	Incident cases	Incidence rate	Cases change (%)	EAPC	AAPC
Global	635867.93 (530781.78, 770613.31)	29.01 (24.22, 35.16)	757234.61 (632119.62, 924338.12)	25.45 (21.25, 31.07)	19.09 (13.56, 24.02)	−0.67 (−0.76, −0.58)	−0.115 (−0.119, −0.111)
SDI							
High SDI	81380.38 (66030.13, 99921.07)	23.45 (19.03, 28.80)	69221.71 (55379.35, 86111.87)	19.60 (15.68, 24.38)	−14.94 (−17.97, −12.29)	−0.80 (−0.90, −0.70)	−0.124 (−0.126, −0.122)
High-middle SDI	144626.60 (121130.07, 174311.74)	31.96 (26.77, 38.52)	118406.63 (98129.15, 144548.77)	26.89 (22.29, 32.83)	−18.13 (−22.71, −13.64)	−0.89 (−1.01, −0.77)	−0.166 (−0.173, −0.158)
Middle SDI	222297.51 (185298.99, 269106.61)	29.54 (24.62, 35.76)	243882.45 (203975.05, 297448.61)	26.29 (21.99, 32.07)	9.71 (2.98, 16.05)	−0.67 (−0.78, −0.57)	−0.106 (−0.110, −0.102)
Low-middle SDI	129606.49 (108082.82, 156659.25)	28.59 (23.84, 34.55)	212570.36 (179589.10, 258338.37)	26.49 (22.38, 32.19)	64.01 (56.68, 70.41)	−0.41 (−0.48, −0.34)	−0.064 (−0.069, −0.060)
Low SDI	57356.42 (47891.44, 69984.35)	31.12 (25.98, 37.97)	112565.00 (94143.46, 137847.50)	25.07 (20.96, 30.70)	96.26 (88.52, 103.48)	−0.83 (−0.89, −0.77)	−0.195 (−0.196, −0.193)
Regions							
Andean Latin America	4980.64 (4316.98, 5888.32)	32.21 (27.92, 38.08)	6493.75 (5477.44, 7813.55)	23.98 (20.23, 28.85)	30.38 (22.84, 37.97)	−1.09 (−1.17, −1.01)	−0.262 (−0.269, −0.255)
Australasia	1439.55 (1188.18, 1757.78)	17.65 (14.57, 21.56)	1496.18 (1207.88, 1869.18)	14.29 (11.54, 17.85)	3.93 (−2.67, 11.26)	−0.78 (−0.89, −0.68)	−0.108 (−0.110, −0.107)
Caribbean	4057.19 (3454.97, 4917.55)	27.29 (23.24, 33.08)	4729.47 (4067.87, 5635.65)	25.98 (22.35, 30.96)	16.57 (11.95, 21.70)	−0.39 (−0.48, −0.30)	−0.042 (−0.047, −0.038)
Central Asia	11738.37 (10074.67, 14190.04)	41.25 (35.41, 49.87)	13067.83 (11122.63, 15731.78)	34.95 (29.75, 42.08)	11.33 (7.05, 15.75)	−0.82 (−0.95, −0.68)	−0.193 (−0.209, −0.177)
Central Europe	15016.93 (12727.93, 18023.05)	32.05 (27.17, 38.47)	7425.65 (6079.67, 9189.20)	21.20 (17.36, 26.24)	−50.55 (−53.28, −47.98)	−1.50 (−1.68, −1.32)	−0.347 (−0.352, −0.343)
Central Latin America	17768.76 (14761.29, 21976.53)	26.03 (21.62, 32.19)	19935.21 (16210.27, 24710.43)	19.71 (16.02, 24.43)	12.19 (7.06, 17.38)	−1.16 (−1.32,.−1.01)	−0.207 (−0.214, −0.200)
Central Sub-Saharan Africa	6191.15 (5130.08, 7605.41)	29.82 (24.71, 36.63)	13299.34 (11092.74, 16415.07)	24.58 (20.51, 30.34)	114.81 (104.53, 125.18)	−0.72 (−0.77, −0.68)	−0.167 (−0.169, −0.165)
East Asia	160259.06 (131424.16, 195126.45)	28.33 (23.23, 34.49)	129650.38 (107473.57, 158203.34)	27.06 (22.43, 33.02)	−19.10 (−26.40, −11.58)	−0.62 (−0.79, −0.44)	−0.042 (−0.050, −0.035)
Eastern Europe	31311.01 (25629.93, 38416.71)	36.51 (29.88, 44.79)	24434.81 (20470.82, 29863.37)	36.93 (30.94, 45.13)	−21.96 (−26.09, −17.76)	0.01 (−0.07, 0.09)	0.029 (0.001, 0.056)
Eastern Sub-Saharan Africa	25423.86 (21338.77, 30848.55)	35.86 (30.10, 43.52)	45362.91 (37851.22, 55585.61)	25.89 (21.61, 31.73)	78.43 (69.63, 87.23)	−1.23 (−1.31, −1.15)	−0.323 (−0.326, −0.321)
High-income Asia Pacific	18708.09 (15117.14, 23160.86)	27.72 (22.40, 34.31)	11246.65 (8736.03, 14344.23)	22.25 (17.29, 28.38)	−39.88 (−44.43, −35.58)	−0.99 (−1.08, −0.90)	−0.180 (−0.190, −0.170)
High-income North America	23844.74 (18439.37, 30628.23)	21.04 (16.27, 27.03)	22367.35 (17564.04, 28744.41)	18.16 (14.26, 23.33)	−6.20 (−10.60, −1.92)	−0.67 (−0.80, −0.54)	−0.096 (−0.105, −0.088)
North Africa and Middle East	46246.05 (39538.24, 55690.70)	34.56 (29.54, 41.61)	73765.73 (62408.08, 88840.92)	29.01 (24.54, 34.94)	59.51 (53.09, 66.76)	−0.71 (−0.77, −0.65)	−0.175 (−0.180, −0.169)
Oceania	772.87 (657.13, 926.89)	29.09 (24.74, 34.89)	1412.05 (1199.39, 1691.36)	25.06 (21.29, 30.02)	82.70 (75.85, 90.55)	−0.64 (−0.72, −0.56)	−0.132 (−0.135, −0.129)
South Asia	106654.68 (87016.17, 131066.31)	24.71 (20.16, 30.37)	183854.67 (151305.27, 225946.66)	23.25 (19.13, 28.57)	72.38 (63.88, 79.64)	−0.39 (−0.48, −0.30)	−0.050 (−0.054, −0.046)
Southeast Asia	72260.48 (60837.29, 86987.99)	36.68 (30.88, 44.16)	93906.57 (80668.77, 111978.70)	33.86 (29.09, 40.38)	29.96 (24.35, 35.49)	−0.40 (−0.50, −0.30)	−0.092 (−0.098, −0.087)
Southern Latin America	6435.67 (5494.53, 7666.40)	33.73 (28.80, 40.18)	5687.29 (4653.90, 6920.73)	22.05 (18.04, 26.83)	−11.63 (−18.00, −6.15)	−1.64 (−1.85, −1.43)	−0.382 (−0.387, −0.377)
Southern Sub-Saharan Africa	7185.94 (5886.23, 8877.53)	33.24 (27.23, 41.07)	8201.45 (6693.97, 10150.29)	24.10 (19.67, 29.82)	14.13 (5.94, 21.54)	−1.47 (−1.73, −1.22)	−0.288 (−0.295, −0.280)
Tropical Latin America	22356.55 (18617.56, 27063.95)	34.76 (28.95, 42.08)	16954.06 (13822.29, 20945.39)	19.20 (15.65, 23.72)	−24.17 (−30.63, −17.48)	−2.37 (−2.69, −2.05)	−0.505 (−0.519, −0.491)
Western Europe	29275.58 (24105.24, 35846.95)	20.31 (16.73, 24.87)	18546.43 (14656.60, 23267.35)	14.29 (11.29, 17.93)	−36.65 (−40.47, −33.32)	−1.46 (−1.60, −1.33)	−0.195 (−0.198, −0.191)
Western Sub-Saharan Africa	23940.77 (19896.30, 29455.76)	33.45 (27.80, 41.15)	55396.84 (46523.61, 68037.62)	28.97 (24.33, 35.58)	131.39 (123.85, 139.03)	−0.52 (−0.59, −0.45)	−0.143 (−0.147, −0.138)

EAPC, estimated annual percentage change; AAPC, average annual percentage change; CI, confidence interval; GBD, Global Burden of Disease; SDI, socio-demographic index; UI, uncertainty interval.

**Table 2:** Age-standardized morality rate and AAPC of stroke in individuals aged 15–39 years at global and regional levels, 1990–2021.

Location	Rate per 100,000 (95% UI)						
	1990		2021		1990–2021		
	Mortality cases	Mortality rate	Mortality cases	Mortality rate	Cases change (%)	EAPC	AAPC
Global	133655.14 (125459.26, 141704.20)	6.10 (5.72, 6.47)	122742.66 (113243.10, 133292.05)	4.13 (3.81, 4.48)	−8.16 (−16.81, 1.95)	−1.41 (−1.52, −1.31)	−0.065 (−0.068, −0.063)
SDI							
High SDI	11446.30 (11082.18, 11836.13)	3.30 (3.19, 3.41)	6098.67 (5627.11, 6709.62)	1.73 (1.59, 1.90)	−46.72 (−51.29, −40.77)	−2.12 (−2.29, −1.95)	−0.051 (−0.052, −0.050)
High-middle SDI	28656.11 (26147.22, 30905.25)	6.33 (5.78, 6.83)	17430.14 (15633.61, 19337.53)	3.96 (3.55, 4.39)	−39.17 (−46.99, −30.11)	−1.87 (−2.04, −1.69)	−0.077 (−0.084, −0.070)
Middle SDI	51735.51 (48122.27, 55321.49)	6.87 (6.39, 7.35)	42785.06 (39177.94, 46646.02)	4.61 (4.22, 5.03)	−17.30 (−25.96, −7.57)	−1.41 (−1.53, −1.29)	−0.075 (−0.078, −0.073)
Low-middle SDI	30412.48 (27595.99, 33348.61)	6.71 (6.09, 7.36)	37134.37 (33326.89, 41466.21)	4.63 (4.15, 5.17)	22.10 (6.47, 41.53)	−1.28 (−1.41, −1.16)	−0.066 (−0.067, −0.064)
Low SDI	11266.67 (9743.16, 12893.83)	6.11 (5.29, 7.00)	19172.77 (16714.16, 21737.48)	4.27 (3.72, 4.84)	70.17 (43.05, 97.88)	−1.28 (−1.35, −1.21)	−0.060 (−0.062, −0.059)
Regions							
Andean Latin America	1263.50 (1123.33, 1423.23)	8.17 (7.26, 9.20)	1095.98 (892.55, 1313.35)	4.05 (3.30, 4.85)	−13.26 (−32.41, 8.55)	−2.37 (−2.56, −2.17)	−0.120 (−0.131, −0.108)
Australasia	143.06 (133.12, 153.18)	1.75 (1.63, 1.88)	73.40 (66.70, 81.02)	0.70 (0.64, 0.77)	−48.69 (−54.02, −42.48)	−3.46 (−3.77, −3.15)	−0.033 (−0.035, −0.031)
Caribbean	1015.13 (907.68, 1133.95)	6.83 (6.11, 7.63)	1085.32 (868.29, 1361.46)	5.96 (4.77, 7.48)	6.91 (−13.54, 32.29)	−0.19 (−0.39, 0.00)	−0.029 (−0.038, −0.020)
Central Asia	1755.32 (1662.52, 1840.39)	6.17 (5.84, 6.47)	1412.26 (1227.81, 1604.26)	3.78 (3.28, 4.29)	−19.54 (−30.31, −9.17)	−2.55 (−2.89, −2.21)	−0.079 (−0.093, −0.066)
Central Europe	2771.48 (2677.61, 2860.66)	5.92 (5.72, 6.11)	794.68 (724.18, 855.56)	2.27 (2.07, 2.44)	−71.33 (−73.82, −68.75)	−3.21 (−3.36, −3.06)	−0.117 (−0.121, −0.114)
Central Latin America	2833.77 (2747.43, 2912.23)	4.15 (4.02, 4.27)	2758.92 (2434.93, 3078.57)	2.73 (2.41, 3.04)	−2.64 (−14.78, 8.92)	−1.46 (−1.77, −1.15)	−0.044 (−0.047, −0.042)
Central Sub-Saharan Africa	1029.23 (776.76, 1318.95)	4.96 (3.74, 6.35)	1920.04 (1398.47, 2479.67)	3.55 (2.59, 4.58)	86.55 (42.61, 150.80)	−1.16 (−1.21, −1.12)	−0.046 (−0.049, −0.043)
East Asia	40340.17 (35103.19, 46066.82)	7.13 (6.21, 8.14)	24330.42 (20257.31, 28408.55)	5.08 (4.23, 5.93)	−39.69 (−52.12, −24.60)	−1.41 (−1.65, −1.18)	−0.067 (−0.073, −0.061)
Eastern Europe	4138.11 (3962.58, 4283.79)	4.82 (4.62, 4.99)	3594.79 (3258.44, 3899.69)	5.43 (4.92, 5.89)	−13.13 (−21.20, −4.08)	−0.34 (−0.75, 0.08)	0.022 (0.011, 0.033)
Eastern Sub-Saharan Africa	5365.71 (4648.83, 6334.22)	7.57 (6.56, 8.94)	8182.59 (6889.35, 9594.77)	4.67 (3.93, 5.48)	52.50 (16.99, 84.92)	−1.78 (−1.89, −1.67)	−0.096 (−0.098, −0.094)
High-income Asia Pacific	2826.23 (2601.38, 3087.38)	4.19 (3.85, 4.57)	822.35 (776.95, 889.57)	1.63 (1.54, 1.76)	−70.90 (−73.86, −67.57)	−3.21 (−3.35, −3.06)	−0.081 (−0.084, −0.078)
High-income North America	2589.18 (2520.67, 2661.03)	2.28 (2.22, 2.35)	1742.34 (1630.74, 1844.08)	1.41 (1.32, 1.50)	−32.71 (−37.56, −28.58)	−1.67 (−1.90, −1.43)	−0.026 (−0.028, −0.025)
North Africa and Middle East	10628.10 (9406.83, 11908.17)	7.94 (7.03, 8.90)	11560.03 (9659.74, 13694.93)	4.55 (3.80, 5.39)	8.77 (−7.99, 30.79)	−1.77 (−1.86, −1.67)	−0.105 (−0.108, −0.102)
Oceania	273.76 (197.74, 373.24)	10.31 (7.44, 14.05)	505.98 (363.81, 668.66)	8.98 (6.46, 11.87)	84.83 (32.19, 159.77)	−0.58 (−0.67, −0.48)	−0.040 (−0.049, −0.031)
South Asia	20393.91 (17936.05, 23112.47)	4.73 (4.16, 5.35)	26280.88 (23405.44, 29365.15)	3.32 (2.96, 3.71)	28.87 (7.61, 53.32)	−1.20 (−1.38, −1.02)	−0.043 (−0.047, −0.038)
Southeast Asia	19723.04 (17991.90, 21674.16)	10.01 (9.13, 11.00)	22700.88 (19792.89, 26209.35)	8.19 (7.14, 9.45)	15.10 (−1.70, 36.84)	−0.67 (−0.84, −0.50)	−0.062 (−0.065, −0.058)
Southern Latin America	1289.65 (1223.39, 1354.93)	6.76 (6.41, 7.10)	563.97 (525.60, 603.99)	2.19 (2.04, 2.34)	−56.27 (−60.10, −52.56)	−3.44 (−3.73, −3.14)	−0.143 (−0.149, −0.138)
Southern Sub-Saharan Africa	1513.88 (1377.51, 1656.10)	7.00 (6.37, 7.66)	1576.54 (1388.14, 1795.09)	4.63 (4.08, 5.27)	4.14 (−9.31, 21.66)	−1.52 (−2.41, −0.62)	−0.096 (−0.110, −0.081)
Tropical Latin America	5585.34 (5437.42, 5737.60)	8.68 (8.45, 8.92)	3193.70 (3071.67, 3317.06)	3.62 (3.48, 3.76)	−42.82 (−45.44, −40.03)	−3.12 (−3.37, −2.87)	−0.164 (−0.170, −0.159)
Western Europe	4228.98 (4117.33, 4333.56)	2.93 (2.86, 3.01)	1053.21 (1012.43, 1093.17)	0.81 (0.78, 0.84)	−75.10 (−76.28, −73.94)	−4.27 (−4.38, −4.15)	−0.068 (−0.070, −0.066)
Western Sub-Saharan Africa	3947.59 (3377.99, 4511.04)	5.52 (4.72, 6.30)	7494.37 (6103.57, 8868.39)	3.92 (3.19, 4.64)	89.85 (57.96, 130.80)	−1.08 (−1.19, −0.97)	−0.050 (−0.052, −0.049)

EAPC, estimated annual percentage change; AAPC, average annual percentage change; CI, confidence interval; GBD, Global Burden of Disease; SDI, socio-demographic.

### Regional trend in stroke incidence, mortality and DALY rate in five SDI regions

From 1990 to 2021, both mortality and DALY cases increased in the low-middle and low-SDI regions. In the low-middle SDI group, mortality rose by 22.10% (95% UI: 6.47–41.53%) (Table 2), and DALYs increased by 25.77% (95% UI: 11.05–43.12%) (Supplementary Table S1). In the low SDI group, mortality increased by 70.17% (95% UI: 43.05–97.88%) (Table 2), and DALYs rose by 75.36% (95% UI: 50.23–100.50%) (Supplementary Table S1). Despite these absolute increases, the average annual percentage change (AAPC) for age-standardized mortality and DALY rates was negative across all five SDI regions, indicating a reduction in age-standardized stroke mortality and DALY burdens over this period. In 2021, the middle SDI region had the highest number of stroke cases, with 243,882.45 (95% UI: 203,975.05-297,448.61), while the low SDI region saw a 96.26% increase in incidence (95% UI: 88.52–103.48%) (Table 1). The greatest reduction in stroke incidence among youths was observed in regions with high to mid-SDI (EAPC = −0.89, 95% CI: −1.01 to −0.77) (Table 1).

### Regional trend in stroke incidence, mortality and DALY rate in 21 GBD regions

In terms of incidence, South Asia had the highest number of stroke cases in youths and young adults aged 15–39 years in 2021, with 183,854.67 cases (95% UI: 151,305.27–225,946.66), while Oceania had the fewest, with 1,412.05 cases (95% UI: 1,199.39–1,691.36). The highest stroke incidence rate among youths and young adults was observed in Eastern Europe (36.93; 95% UI: 30.94–45.13). In contrast, Australasia and Western Europe had the lowest stroke incidence rates, both at 14.29 (95% UI: 11.54–17.85 and 95% UI: 11.29–17.93, respectively). From 1990 to 2021, the incidence rate of stroke in youths and young adults increased only in Eastern Europe (EAPC: 0.01; 95% CI: −0.07–0.09). The largest decline in stroke incidence was seen in the High-income Asia Pacific region (EAPC: -2.06; 95% CI: −2.17 to −1.94) (Table 1).

In 2021, East Asia had the highest number of stroke-related deaths among youths and young adults aged 15–39 years, with 24,330.42 deaths (95% UI: 20,257.31–28,408.55). Oceania had the highest stroke-related mortality rate for youths and young adults (8.98; 95% UI: 6.46–11.87). The Caribbean experienced the smallest decrease in stroke-related mortality rate (EAPC: −0.19; 95% CI: −0.39 to 0.00), while Australasia saw the largest reduction (EAPC: −3.46; 95% CI: −3.77 to −3.15) (Table 2).

**Figure 1:**
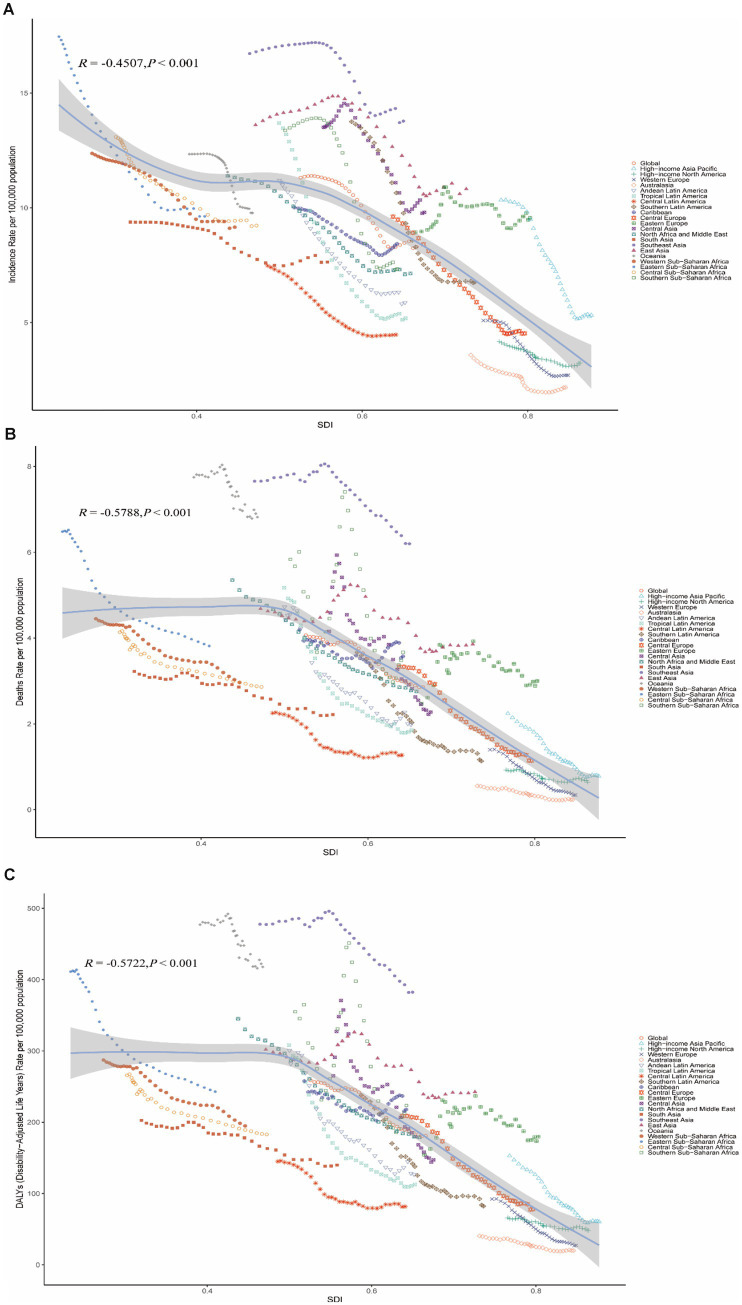
The association between the socio-demographic index and young stroke in youths and young adults aged 15–39 years across 21 GBD regions. **(A)** The relationship between age-standardized stroke incidence and socio-demographic index. **(B)** The relationship between age-standardized stroke mortality and socio-demographic index. **(C)** The relationship between age-standardized stroke disability-adjusted life years and socio-demographic index.

In 2021, South Asia had the highest number of disability-adjusted life years (DALYs) related to stroke in youths and young adults (1,837,332.75; 95% UI, 1,622,972.92–2,033,261.45), while Australasia had the lowest (7,611.68; 95% UI, 6,516.81–8,696.71). Oceania reported the highest DALY rate (599.51; 95% UI, 452.16–778.14), while Australasia had the lowest DALY rate (72.69; 95% UI, 62.24–83.06) (Table 1). Figure 1 shows a negative correlation between the age-standardized prevalence rate, mortality rate, and disability-adjusted life years (DALY) rate for young stroke in youths and young adults aged 15–39 years and the Socio-Demographic Index (SDI). In 2021, the global SDI was 0.37. Eight regions, including Eastern Europe, Southeast Asia, and High-Income North America, had prevalence rates higher than the global average (Figure 1A), while another eight regions exceeded the global average mortality rate (Figure 1B). Additionally, eight regions reported DALY rates above the global average (Figure 1C).

### National trend in stroke incidence, mortality and DALY rate

**Figure 2:**
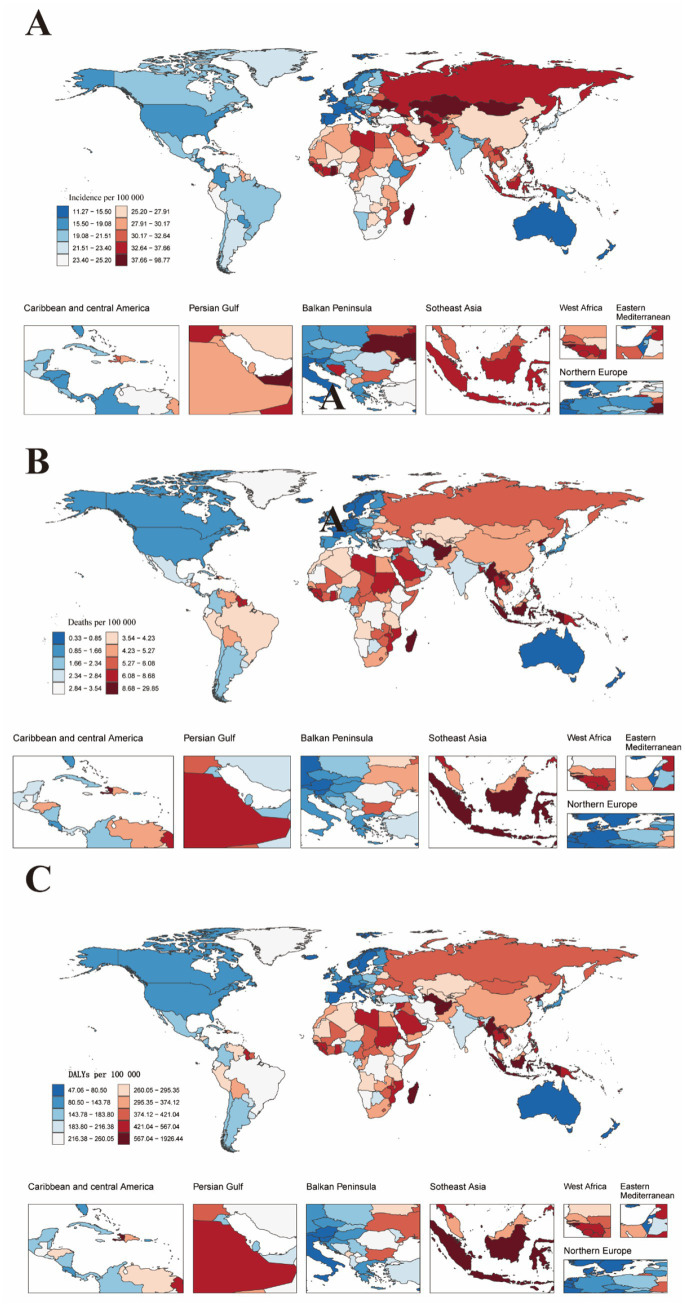
Global maps showing the age-standardized incidence rate, mortality rate, and DALYs rate of stroke in youths and young adults aged 15–39 years for both sexes in 2021. DALYs refer to disability-adjusted life years. **(A)** Age-standardized incidence rates of stroke in youths and young adults aged 15–39 years for both sexes combined across 204 countries worldwide. **(B)** Age-standardized mortality rates of stroke in youths and young adults aged 15–39 years for both sexes combined across 204 countries worldwide. **(C)** Age-standardized disability-adjusted life years rates of stroke in youths and young adults aged 15–39 years for both sexes combined across 204 countries worldwide.

From 1990 to 2021, the global incidence of stroke among individuals aged 15 to 39 varied significantly across countries. In 2021, India reported the highest number of young stroke cases at 131,103.66 (95% UI, 106,504.02–162,808.88) among the 204 countries analyzed, while Tokelau had the lowest incidence, with just 0.19 cases per 100,000 people (95% UI, 0.17–0.22). Kiribati recorded the highest incidence rate of young stroke cases in 2021, at 97.8 per 100,000 people (95% UI, 88.46–107.52), while Portugal had the lowest rate, at 11.39 per 100,000 people (95% UI, 8.65–15.01). The Philippines saw the largest increase in incidence (EAPC, 2.23; 95% CI, 1.92–2.54), while Rwanda experienced the greatest decrease (EAPC, −3.16; 95% CI, −3.50 to −2.81). The global incidence rate for young stroke in 2021 was 25.45 per 100,000 people (95% UI, 21.25–31.07), with 101 countries reporting rates above and 103 countries below the global average (Supplementary Table S2; Figure 2A).

In 2021, China reported the highest number of stroke-related deaths among youths and young adults, with 23,099.87 cases (95% UI, 19,113.48–27,336.47). Nauru had the highest stroke-related mortality rate for this age group, at 29.56 per 100,000 people (95% CI, 21.22–42.56), while Slovenia (SDI 0.84) recorded the lowest rate at 0.33 per 100,000 people (95% CI, 0.27–0.41). Zimbabwe experienced the largest increase in mortality (EAPC, 4.72; 95% CI, 3.56–5.90), while Luxembourg (EAPC, −7.39; 95% CI, −7.73 to −7.06) and Slovenia (EAPC, −6.67; 95% CI, −6.96 to −6.39) showed the greatest decreases. The global stroke-related mortality rate for youths and young adults in 2021 was 4.13 per 100,000 people (95% UI, 3.81–4.48), with 87 countries reporting rates above the global average and 117 countries below it (Supplementary Table S3; Figure 2B).

In 2021, China had the highest number of stroke-related disability-adjusted life years (DALYs) among youths and young adults, totaling 1,654,368.25 (95% UI, 1,394,426.09–1,906,661.45). Nauru recorded the highest DALY rate for young stroke cases, at 1,907.37 per 100,000 people (95% UI, 1,411.15–2,672.63). Zimbabwe experienced the largest increase in DALY rate (EAPC, 3.97; 95% CI, 2.99–4.96), while Luxembourg (EAPC, −5.64; 95% CI, −5.89 to −5.38) and Rwanda (EAPC, −5.33; 95% CI, −5.94 to −4.72) showed the greatest decreases. In 2021, the global DALY rate for young stroke cases was 293.08 per 100,000 people (95% UI, 267.28–318.41), with 86 countries/regions reporting rates above the global average and 118 countries/regions below it (Supplementary Table S4; Figure 2C).

### Factors influencing EAPCs

EAPCs showed significant differences compared to the incidence, mortality, and disability-adjusted life years rates in 2021. Furthermore, there were also notable differences between EAPCs and the 2021 Socio-Demographic Index. Specifically, EAPCs were positively correlated with DALY rates (Pearson *r* = 0.54; *p* < 0.001), and they also exhibited positive correlations with incidence rates (Pearson *r* = 0.51; *p* < 0.001) and mortality rates (Pearson *r* = 0.56; *p* < 0.001). In contrast, the EAPCs for incidence, mortality, and DALYs were negatively correlated with the SDI (Supplementary Figure S1).

### Stroke-related deaths attributable to major risk factors by SDI level

The results highlight the proportion of stroke-related deaths in youths and young adults aged 15–39 years for both sexes attributable to various risk factors, both globally and across different Socio-Demographic Index levels: High, High-Middle, Middle, Low-Middle, and Low SDI. The key risk factors analyzed include ambient air pollution, high sodium intake, low fruit intake, tobacco use, and exposure to secondhand smoke. Globally, metabolic risks (46.2%) and high systolic blood pressure (37.87%) are major contributors to stroke-related mortality in youths and young adults aged 15–39 years for both sexes. The impact of these risk factors varies by SDI level: low SDI regions are predominantly affected by environmental and occupational risks as well as metabolic factors, while high SDI regions experience a greater influence from behavioral and metabolic risks. These findings highlight the distinct public health challenges faced by different socio-economic groups and emphasize the need for targeted intervention strategies (Supplementary Figure S2).

## Discussion

This study provides a comprehensive analysis of the global, regional, and national trends in stroke incidence, mortality, and disability-adjusted life years among youths and young adults aged 15–39 years from 1990 to 2021. Our findings reveal significant variations in stroke burden across different SDI regions, with notable disparities between high and low SDI regions.

### Global trends

During the study period, the global age-standardized incidence of stroke increased by 19.09%, reflecting a growing global burden of stroke. From 1990 to 2021, both the global incidence rate and the prevalence of stroke in the general population rose by over 70%, which aligns with our findings. However, despite this increase, the age-standardized stroke incidence showed a gradual decline, with an estimated annual percentage change of −0.67, indicating a decrease in overall incidence relative to the global population (22). This suggests that while the burden of stroke continues to rise, the rate of incidence has been stabilizing over time. Notably, in 2021, the global stroke incidence continued to increase, further highlighting the ongoing global burden (23).

### Regional trends

At the regional level, the low-middle and low SDI regions experienced a significant rise in both mortality and DALYs, with mortality increasing by 22.10 and 70.17%, highlighting the growing health challenge posed by stroke in low-resource settings (24). These increases are likely driven by limited healthcare infrastructure, insufficient stroke prevention programs, delayed access to acute care, and cultural and lifestyle factors such as high-salt and high-fat diets, sedentary lifestyles, and higher rates of smoking and alcohol consumption. These challenges, compounded by inadequate access to healthcare education, significantly exacerbate the risk of stroke and emphasize the urgent need for targeted prevention strategies (25).

In low SDI regions, where the increase in stroke burden is most pronounced, implementing targeted prevention strategies, improving healthcare infrastructure, and enhancing access to treatment are critical to reducing the burden (26, 27). Despite the absolute increase in stroke burden, the negative average annual percentage change for both mortality and DALY rates across all SDI regions reflects a decline in age-standardized rates over time, particularly in high-SDI regions. Addressing these disparities in low-resource settings is essential to prevent further escalation of the stroke burden and improve health outcomes in these populations (28).

### National trends

At the national level, India reported the highest number of stroke cases among youths and young adults in 2021, reflecting the country’s high population density and the rising burden of non-communicable diseases. In contrast, countries like Tokelau recorded the lowest incidence rates, likely due to a smaller population and specific regional health factors. The Philippines experienced the largest increase in stroke incidence, while Rwanda saw the greatest decline, potentially reflecting differences in national health policies, healthcare system improvements, and increased awareness of stroke risk factors.

The wide variability in stroke incidence, mortality, and DALYs among countries highlights disparities in healthcare access, quality, and stroke prevention efforts (29). Nations with high stroke incidence and mortality, such as Zimbabwe, could benefit from intensified stroke prevention, early detection, and treatment initiatives. Conversely, countries with significant declines in stroke-related outcomes, like Luxembourg and Rwanda, demonstrate how enhancements in healthcare infrastructure and stroke management can improve outcomes for youths and young adults (1).

### Stroke incidence and mortality trends by region

The regional analysis shows that Eastern Europe had the highest stroke incidence rate, indicating a heavy burden of stroke due to factors such as high rates of smoking, unhealthy diets, and limited healthcare access. In contrast, Australasia and Western Europe reported the lowest incidence rates, likely due to better healthcare systems and effective preventive measures. The High-Income Asia Pacific region experienced the largest decline in stroke incidence, reflecting the impact of strong healthcare systems, public health initiatives, and improved stroke care. Significant disparities were also noted in stroke-related mortality and DALY rates, with Oceania recording the highest figures, potentially linked to higher obesity and hypertension rates as well as restricted healthcare access (30, 31).

### Implications for public health

The findings of this study have important public health implications. First, targeted stroke prevention programs are essential, particularly in low-middle and low SDI regions where the burden is rising (32). These programs should focus on managing key risk factors such as hypertension, diabetes, and smoking, while also considering the influence of cultural and socioeconomic factors on health behaviors and access to care. Second, improving healthcare infrastructure and access to acute stroke care in low-resource regions is critical to reducing stroke mortality and DALYs (33). Economic disparities limit access to advanced treatments, widening the gap in outcomes. Countries with strong healthcare systems, such as those in Western Europe, have successfully reduced stroke-related mortality and DALY rates through effective policies, early detection, and emergency response systems, providing valuable models for other regions.

Finally, ongoing research and surveillance are needed to track stroke trends and assess intervention effectiveness. Policy measures, including tobacco control, dietary regulations, and expanded healthcare coverage, can help mitigate regional disparities. Further studies should examine the interplay of economic development, healthcare policies, and cultural factors to inform tailored strategies (34, 35).

### Limitations

This study has several limitations. First, the analysis relies on data from the GBD study, which may be affected by reporting inconsistencies and data quality issues, especially in regions with limited healthcare infrastructure. Second, while the study includes ASRs, it does not fully account for potential confounders such as socioeconomic status, healthcare access, and lifestyle factors, which could influence stroke incidence and outcomes. Additionally, the use of estimated data and uncertainty intervals may introduce variability in the results, particularly in regions with sparse data. Finally, the study does not explore underlying causes or specific risk factors for stroke, limiting the understanding of its etiology in different populations.

## Conclusion

In conclusion, this study provides a detailed assessment of the burden of stroke among youths and young adults globally, regionally, and nationally. While the global trend shows a decline in age-standardized stroke incidence, mortality, and DALYs, significant disparities persist across SDI regions and countries. Addressing these disparities through targeted prevention, improved healthcare access and better stroke management is essential to reducing the stroke burden, particularly in low and middle-income countries. Further research and policy interventions are needed to sustain these improvements and tackle the rising stroke burden in certain regions.

## Data Availability

The original contributions presented in the study are included in the article/Supplementary material, further inquiries can be directed to the corresponding authors.

## Footnotes

^1^ https://ghdx.healthdata.org/gbd-2021/sources
