# Supplementary material for: Global, regional, and national burden and trends of stroke among youths and young adults aged 15–39 years from 1990 to 2021: findings from the Global Burden of Disease study 2021
Source: Front Neurol. 2025 Mar 7;16:1535278. doi: 10.3389/fneur.2025.1535278 (PMC11938946; doi:10.3389/fneur.2025.1535278)
Supplement: Supplementary file 1 [file Supplementary_file_1.docx]

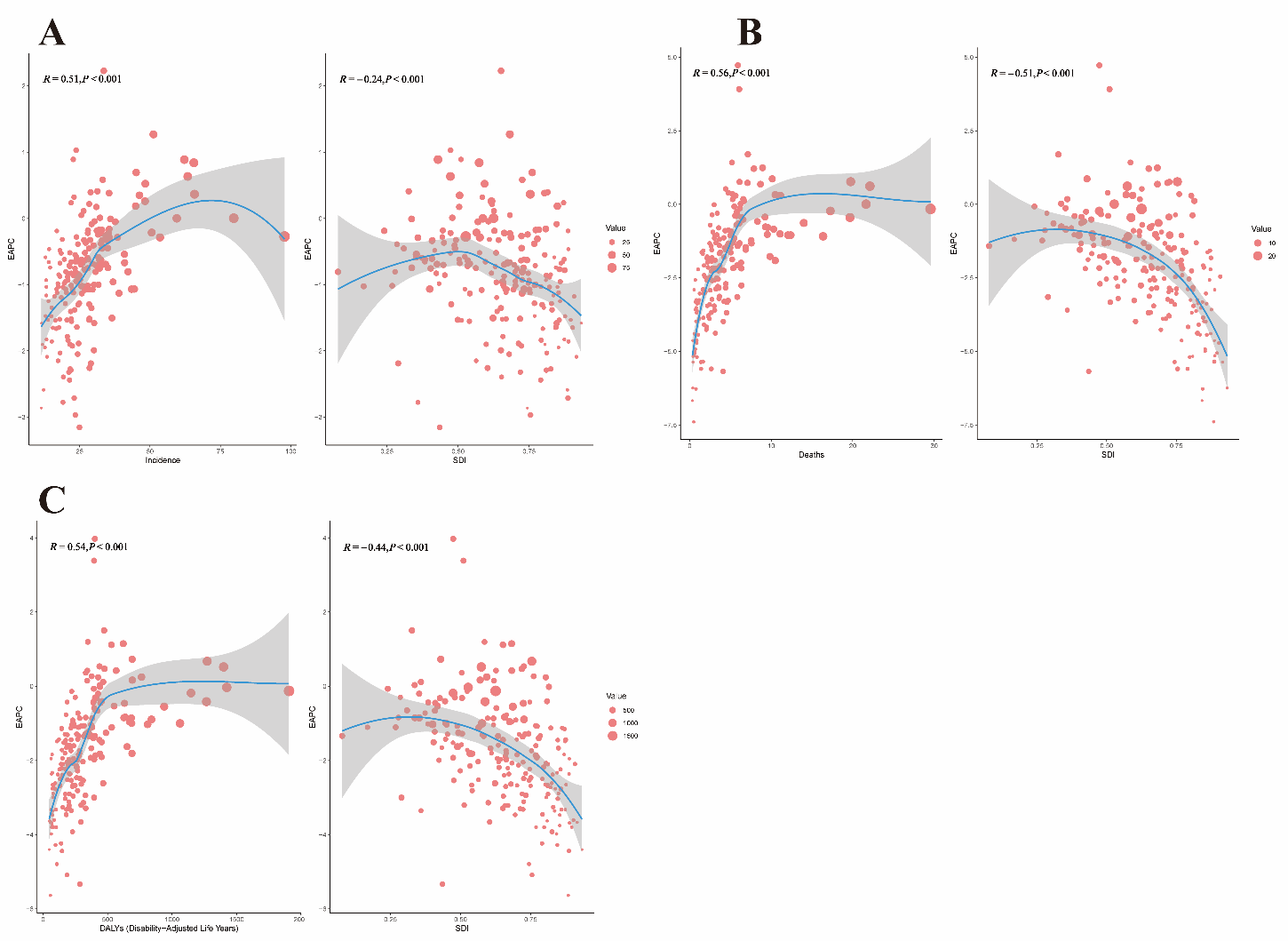


Figure. S1 Correlation analysis between EAPC and stroke in youths and young adults aged 15-39 years for both sexes in 2021. A. Correlation analysis between EAPC and stroke in youths and young adults aged 15-39 years for both sexes incidence rates in 2021. B. Correlation analysis between EAPC and stroke in youths and young adults aged 15-39 years for both sexes mortality rates in 2021. C. Correlation analysis between EAPC and stroke in youths and young adults aged 15-39 years for both sexes disability-adjusted life years rates in 2021.

**
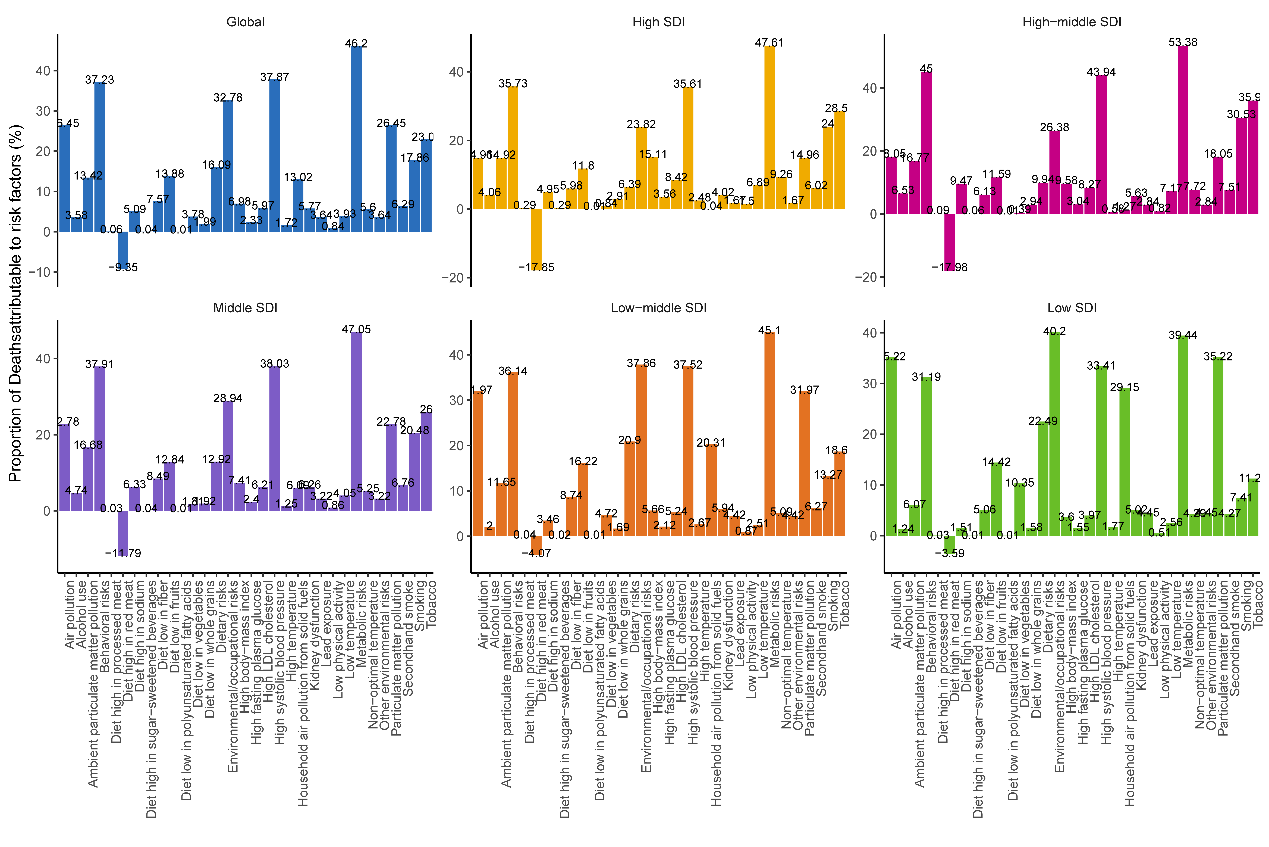
**

Figure. S2 Global and regional distribution of stroke-related deaths in youths and young adults aged 15-39 years for both sexes, attributable to major risk factors, by SDI level.

| **Table S1. Age-standardized DALY rate and AAPC of Stroke in Individuals Aged 15-39 Years at Global and Regional Levels, 1990-2021** | | | | | | | | | | | |
| --- | --- | --- | --- | --- | --- | --- | --- | --- | --- | --- | --- |
| **Location** | **Rate per 100 000 (95% UI)** |  |  |  |  | |  | |  | |  |
|  | **1990** |  | **2021** |  | **1990-2021** |  | |  | |  |  |
|  | **DALYs cases** | **DALYs rate** | **DALYs cases** | **DALYs rate** | **Cases change(%)** | | **EAPC** | | **AAPC** | |  |
| **Global** | 9223402.97(8625790.83,9847842.05) | 420.81(393.55,449.30) | 8718567.32(7951154.52,9472143.29) | 293.08(267.28,318.41) | -5.47(-13.42,2.89) | | -1.31(-1.40,-1.22) | | -4.176(-4.301,-4.052) | |  |
| **SDI** |  |  |  |  |  | |  | |  | |  |
| High SDI | 860541.18(793411.57,920347.24) | 248.02(228.67,265.26) | 531636.51(477135.38,597299.02) | 150.50(135.07,169.09) | -38.22(-42.85,-33.29) | | -1.64(-1.76,-1.53) | | -3.174(-3.233,-3.116) | |  |
| High-middle SDI | 2018854.34(1846284.54,2192689.30) | 446.12(407.98,484.53) | 1281836.01(1149483.08,1418381.16) | 291.15(261.09,322.16) | -36.51(-43.16,-29.28) | | -1.68(-1.82,-1.54) | | -5.013(-5.415,-4.611) | |  |
| Middle SDI | 3536396.16(3276147.15,3779625.06) | 469.87(435.29,502.19) | 2995803.53(2732634.01,3258038.09) | 323.00(294.63,351.27) | -15.29(-23.05,-7.24) | | -1.33(-1.43,-1.23) | | -4.888(-5.043,-4.733) | |  |
| Low-middle SDI | 2029205.96(1867219.84,2217488.65) | 447.55(411.83,489.08) | 2552173.20(2292929.19,2829818.75) | 318.03(285.73,352.63) | 25.77(11.05,43.12) | | -1.19(-1.30,-1.09) | | -4.093(-4.203,-3.983) | |  |
| Low SDI | 769136.74(678229.73,874859.64) | 417.31(367.99,474.67) | 1348782.55(1178409.31,1516510.54) | 300.36(262.42,337.71) | 75.36(50.23,100.50) | | -1.19(-1.25,-1.13) | | -3.845(-3.920,-3.771) | |  |
| **Regions** |  |  |  |  |  | |  | |  | |  |
| Andean Latin America | 82999.72(74439.17,92844.85) | 536.74(481.38,600.41) | 74083.29(61774.90,87379.70) | 273.58(228.12,322.68) | -10.74(-28.35,8.68) | | -2.26(-2.43,-2.08) | | -7.657(-8.386,-6.928) | |  |
| Australasia | 11234.26(10244.30,12306.90) | 137.78(125.64,150.93) | 7611.68(6516.81,8696.71) | 72.69(62.24,83.06) | -32.25(-38.76,-25.32) | | -2.44(-2.64,-2.24) | | -2.007(-2.122,-1.893) | |  |
| Caribbean | 65108.76(58778.79,71984.07) | 438.01(395.43,484.26) | 69460.69(56398.66,84809.22) | 381.60(309.84,465.92) | 6.68(-12.10,30.03) | | -0.21(-0.39,-0.03) | | -1.630(-2.037,-1.222) | |  |
| Central Asia | 126190.99(118078.23,134527.11) | 443.50(414.98,472.79) | 108607.21(94531.57,121491.18) | 290.50(252.85,324.96) | -13.93(-22.70,-4.86) | | -2.21(-2.49,-1.93) | | -5.074(-5.869,-4.278) | |  |
| Central Europe | 190980.83(179661.82,202093.06) | 407.66(383.50,431.38) | 65415.27(58316.21,72433.19) | 186.80(166.52,206.84) | -65.75(-68.43,-63.15) | | -2.65(-2.78,-2.51) | | -7.122(-7.307,-6.936) | |  |
| Central Latin America | 193734.80(185496.33,201514.52) | 283.79(271.72,295.18) | 191159.39(169931.77,212441.33) | 188.96(167.98,210.00) | -1.33(-11.65,8.93) | | -1.40(-1.67,-1.13) | | -2.972(-3.134,-2.809) | |  |
| Central Sub-Saharan Africa | 71981.61(57284.50,89761.62) | 346.70(275.91,432.33) | 136424.11(103070.52,170806.05) | 252.19(190.53,315.75) | 89.53(50.61,144.50) | | -1.11(-1.15,-1.07) | | -3.110(-3.292,-2.928) | |  |
| East Asia | 2821889.77(2476090.92,3168555.54) | 498.83(437.70,560.11) | 1740447.67(1484384.79,1991204.66) | 363.31(309.86,415.66) | -38.32(-47.93,-26.23) | | -1.31(-1.49,-1.12) | | -4.419(-4.787,-4.051) | |  |
| Eastern Europe | 300266.89(277760.25,324195.40) | 350.09(323.85,377.99) | 247761.92(226118.10,269498.56) | 374.41(341.71,407.26) | -17.49(-23.77,-10.47) | | -0.35(-0.69,-0.01) | | 0.876(0.236,1.515) | |  |
| Eastern Sub-Saharan Africa | 361572.69(315454.16,418629.85) | 510.05(444.99,590.54) | 568966.25(481777.35,657030.47) | 324.78(275.01,375.05) | 57.36(24.14,86.44) | | -1.68(-1.78,-1.57) | | -6.144(-6.281,-6.007) | |  |
| High-income Asia Pacific | 203317.65(185220.09,221405.50) | 301.23(274.42,328.03) | 72524.06(64008.75,80233.04) | 143.50(126.65,158.75) | -64.33(-67.65,-60.90) | | -2.55(-2.67,-2.43) | | -4.914(-5.139,-4.689) | |  |
| High-income North America | 220906.55(199911.64,243179.14) | 194.95(176.42,214.60) | 172582.34(151707.86,195709.00) | 140.10(123.16,158.88) | -21.88(-25.88,-18.09) | | -1.15(-1.25,-1.04) | | -1.745(-1.823,-1.668) | |  |
| North Africa and Middle East | 739248.30(658026.23,824625.98) | 552.38(491.69,616.18) | 846597.38(724750.63,984012.19) | 332.96(285.04,387.00) | 14.52(-0.56,33.91) | | -1.62(-1.70,-1.53) | | -6.814(-6.978,-6.650) | |  |
| Oceania | 18254.21(13765.76,24352.33) | 687.18(518.21,916.75) | 33779.15(25476.74,43843.78) | 599.51(452.16,778.14) | 85.05(37.53,150.25) | | -0.56(-0.65,-0.47) | | -2.661(-3.198,-2.124) | |  |
| South Asia | 1376633.88(1216304.16,1548717.67) | 318.95(281.80,358.82) | 1837332.75(1622972.92,2033261.45) | 232.30(205.20,257.07) | 33.47(14.21,54.52) | | -1.10(-1.26,-0.95) | | -2.666(-2.898,-2.434) | |  |
| Southeast Asia | 1323236.33(1210916.57,1457928.60) | 671.68(614.66,740.05) | 1527297.30(1350592.51,1737939.93) | 550.72(487.00,626.68) | 15.42(0.80,34.15) | | -0.68(-0.82,-0.53) | | -4.064(-4.275,-3.853) | |  |
| Southern Latin America | 86606.90(81551.66,91481.37) | 453.93(427.44,479.48) | 44781.13(41056.88,48633.69) | 173.60(159.16,188.53) | -48.29(-52.33,-44.16) | | -2.97(-3.24,-2.71) | | -9.487(-9.873,-9.102) | |  |
| Southern Sub-Saharan Africa | 99974.78(90967.64,108267.45) | 462.52(420.85,500.88) | 106743.23(95646.74,120297.12) | 313.62(281.02,353.44) | 6.77(-5.76,21.73) | | -1.43(-2.21,-0.63) | | -5.865(-6.708,-5.022) | |  |
| Tropical Latin America | 342477.38(332952.32,352935.10) | 532.52(517.71,548.78) | 204322.25(195044.50,213327.56) | 231.37(220.86,241.57) | -40.34(-42.90,-37.78) | | -2.95(-3.19,-2.71) | | -9.775(-10.101,-9.449) | |  |
| Western Europe | 303889.72(286800.44,322402.89) | 210.86(199.00,223.70) | 101340.54(89061.14,113724.99) | 78.09(68.63,87.63) | -66.65(-69.11,-64.27) | | -3.36(-3.46,-3.25) | | -4.315(-4.422,-4.208) | |  |
| Western Sub-Saharan Africa | 282896.92(247150.35,318231.09) | 395.25(345.31,444.62) | 561329.72(473921.41,646827.86) | 293.57(247.86,338.28) | 98.42(70.72,133.63) | | -0.95(-1.05,-0.86) | | -3.209(-3.300,-3.118) | |  |

Note: EAPC, estimated annual percentage change; AAPC, average annual percentage change; CI, confidence interval; GBD, Global Burden of Disease; SDI, socio-demographic

| **Table S2. Age-standardized incidence due to stroke and their temporal change among youths and young adults (15-39 years) in 204 countries or territories from 1990 to 2021** | | | | | | | | | | | | | | |
| --- | --- | --- | --- | --- | --- | --- | --- | --- | --- | --- | --- | --- | --- | --- |
|  | | **Rate per 100 000 (95% UI)** | | |  | |  | |  | |  | |  | |
|  | **1990** | |  | **2021** | |  | | **1990-2021** | |  | |  | |  |
| **Location** | **Incident cases** | | **Incidence rate** | **Incident cases** | | **Incidence rate** | | **Cases change(%)** | | **EAPC** | | **AAPC** | |  |
| Afghanistan | 1400.11(1188.19,1651.27) | | 44.45(37.73,52.43) | 4284.84(3696.62,4984.21) | | 35.06(30.25,40.78) | | 206.04(182.55,232.92) | | -0.75(-1.18,-0.31) | | -0.302(-0.337,-0.266) | |  |
| Albania | 386.45(321.32,474.02) | | 27.21(22.62,33.37) | 221.54(184.51,273.82) | | 23.37(19.46,28.88) | | -42.67(-45.59,-39.51) | | -0.76(-0.86,-0.66) | | -0.122(-0.128,-0.117) | |  |
| Algeria | 3787.57(3215.00,4562.46) | | 37.48(31.82,45.15) | 5120.31(4336.93,6169.19) | | 30.07(25.47,36.23) | | 35.19(22.85,46.85) | | -1.00(-1.14,-0.87) | | -0.242(-0.254,-0.230) | |  |
| American Samoa | 7.51(6.47,8.91) | | 37.14(31.98,44.06) | 6.12(5.32,7.11) | | 35.01(30.46,40.67) | | -18.58(-23.23,-13.81) | | -0.45(-0.54,-0.36) | | -0.074(-0.079,-0.069) | |  |
| Andorra | 4.05(3.06,5.29) | | 16.20(12.23,21.14) | 3.58(2.67,4.67) | | 14.02(10.47,18.33) | | -11.76(-19.01,-5.54) | | -0.66(-0.77,-0.55) | | -0.065(-0.069,-0.061) | |  |
| Angola | 1270.35(1058.98,1551.06) | | 32.47(27.07,39.64) | 3039.75(2492.14,3764.03) | | 24.99(20.48,30.94) | | 139.28(124.87,155.21) | | -0.86(-0.89,-0.82) | | -0.235(-0.245,-0.226) | |  |
| Antigua and Barbuda | 6.50(5.43,7.91) | | 25.21(21.07,30.68) | 7.17(5.87,8.94) | | 20.83(17.06,26.00) | | 10.22(3.23,18.29) | | -0.93(-1.06,-0.81) | | -0.148(-0.155,-0.140) | |  |
| Argentina | 4525.91(3894.03,5356.70) | | 37.05(31.88,43.85) | 3928.30(3255.96,4765.45) | | 22.42(18.58,27.20) | | -13.20(-20.77,-6.45) | | -1.93(-2.15,-1.70) | | -0.476(-0.482,-0.470) | |  |
| Armenia | 501.50(409.02,625.00) | | 34.89(28.46,43.49) | 300.00(241.26,379.43) | | 27.91(22.44,35.29) | | -40.18(-45.64,-35.06) | | -1.02(-1.23,-0.80) | | -0.227(-0.232,-0.222) | |  |
| Australia | 1201.69(993.56,1461.29) | | 17.74(14.67,21.58) | 1258.62(1024.07,1558.26) | | 14.52(11.81,17.97) | | 4.74(-2.90,12.92) | | -0.74(-0.82,-0.66) | | -0.104(-0.106,-0.103) | |  |
| Austria | 633.34(513.59,818.33) | | 21.10(17.11,27.26) | 479.55(370.28,624.55) | | 16.99(13.12,22.13) | | -24.28(-33.15,-14.05) | | -1.12(-1.27,-0.98) | | -0.137(-0.143,-0.131) | |  |
| Azerbaijan | 1120.71(931.57,1364.16) | | 35.26(29.31,42.92) | 1193.32(976.32,1474.62) | | 28.17(23.05,34.81) | | 6.48(-1.25,15.02) | | -1.08(-1.26,-0.90) | | -0.217(-0.223,-0.210) | |  |
| Bahamas | 30.85(26.28,37.14) | | 26.14(22.27,31.47) | 36.19(30.81,44.03) | | 23.40(19.92,28.47) | | 17.29(11.01,23.91) | | -0.55(-0.63,-0.47) | | -0.092(-0.097,-0.087) | |  |
| Bahrain | 75.81(62.42,94.63) | | 29.57(24.35,36.92) | 160.26(127.65,204.74) | | 22.78(18.14,29.10) | | 111.40(94.20,128.14) | | -1.12(-1.24,-1.00) | | -0.234(-0.255,-0.213) | |  |
| Bangladesh | 13003.44(11048.05,15280.11) | | 30.81(26.17,36.20) | 19773.71(16784.14,23719.51) | | 28.73(24.39,34.47) | | 52.07(42.52,63.77) | | -0.33(-0.41,-0.24) | | -0.061(-0.069,-0.053) | |  |
| Barbados | 28.81(24.18,35.02) | | 26.40(22.16,32.08) | 21.74(17.81,26.90) | | 22.00(18.03,27.23) | | -24.56(-29.05,-20.23) | | -0.83(-0.93,-0.73) | | -0.148(-0.160,-0.137) | |  |
| Belarus | 1413.76(1163.78,1782.60) | | 35.84(29.50,45.19) | 937.07(768.97,1168.43) | | 31.94(26.21,39.83) | | -33.72(-38.39,-28.60) | | -0.17(-0.29,-0.05) | | -0.130(-0.150,-0.111) | |  |
| Belgium | 753.66(597.98,944.17) | | 20.26(16.07,25.38) | 444.62(339.21,579.10) | | 12.69(9.68,16.53) | | -41.00(-47.28,-34.68) | | -1.95(-2.18,-1.71) | | -0.248(-0.255,-0.241) | |  |
| Belize | 14.00(11.43,17.45) | | 19.14(15.63,23.85) | 35.60(29.47,43.46) | | 18.85(15.61,23.02) | | 154.23(139.24,172.13) | | -0.32(-0.46,-0.17) | | -0.008(-0.014,-0.003) | |  |
| Benin | 551.91(457.54,673.04) | | 32.41(26.87,39.53) | 1457.24(1212.49,1810.40) | | 27.80(23.13,34.53) | | 164.03(150.14,179.18) | | -0.60(-0.64,-0.55) | | -0.147(-0.150,-0.144) | |  |
| Bermuda | 6.37(5.09,7.95) | | 24.77(19.77,30.90) | 3.62(2.82,4.65) | | 20.67(16.12,26.54) | | -43.19(-46.81,-39.29) | | -0.88(-1.00,-0.77) | | -0.131(-0.141,-0.122) | |  |
| Bhutan | 53.07(42.42,67.36) | | 19.70(15.74,25.00) | 63.97(50.08,80.96) | | 18.46(14.45,23.36) | | 20.55(10.84,30.75) | | -0.53(-0.65,-0.40) | | -0.041(-0.048,-0.034) | |  |
| Bolivia (Plurinational State of) | 852.77(741.31,1006.43) | | 34.58(30.06,40.81) | 1058.41(884.89,1302.35) | | 21.54(18.01,26.50) | | 24.11(13.66,34.16) | | -1.90(-2.05,-1.76) | | -0.423(-0.428,-0.418) | |  |
| Bosnia and Herzegovina | 690.66(572.52,832.96) | | 36.37(30.15,43.86) | 345.30(289.24,422.64) | | 34.32(28.74,42.00) | | -50.00(-53.48,-46.43) | | -0.27(-0.39,-0.14) | | -0.059(-0.068,-0.051) | |  |
| Botswana | 138.61(113.47,172.11) | | 26.93(22.04,33.43) | 282.77(231.06,351.06) | | 26.54(21.69,32.95) | | 104.01(89.24,120.85) | | -0.33(-0.48,-0.17) | | -0.003(-0.010,0.003) | |  |
| Brazil | 21975.81(18287.45,26632.22) | | 35.02(29.15,42.44) | 16388.51(13362.00,20253.74) | | 19.22(15.67,23.76) | | -25.42(-31.85,-18.81) | | -2.39(-2.72,-2.07) | | -0.512(-0.527,-0.498) | |  |
| Brunei Darussalam | 45.91(38.18,55.42) | | 37.24(30.97,44.95) | 44.81(35.35,56.48) | | 21.96(17.32,27.68) | | -2.39(-11.37,6.82) | | -2.29(-2.58,-2.01) | | -0.505(-0.517,-0.493) | |  |
| Bulgaria | 1108.45(968.88,1296.33) | | 37.24(32.55,43.55) | 619.63(529.66,737.04) | | 32.62(27.88,38.80) | | -44.10(-48.02,-39.72) | | -0.38(-0.44,-0.32) | | -0.149(-0.155,-0.143) | |  |
| Burkina Faso | 865.39(699.31,1101.71) | | 27.15(21.94,34.56) | 2110.51(1721.93,2629.49) | | 24.39(19.90,30.38) | | 143.88(128.64,159.63) | | -0.39(-0.44,-0.33) | | -0.090(-0.094,-0.086) | |  |
| Burundi | 1017.17(878.90,1191.27) | | 49.07(42.40,57.47) | 1528.11(1298.26,1864.06) | | 28.98(24.62,35.36) | | 50.23(39.36,61.73) | | -2.19(-2.45,-1.92) | | -0.649(-0.663,-0.636) | |  |
| Cabo Verde | 46.87(38.97,57.27) | | 35.85(29.80,43.79) | 78.89(64.62,97.26) | | 31.48(25.78,38.81) | | 68.30(52.65,83.60) | | -0.72(-0.84,-0.60) | | -0.135(-0.148,-0.122) | |  |
| Cambodia | 1074.17(888.93,1334.60) | | 27.90(23.09,34.66) | 2021.85(1745.05,2414.09) | | 27.91(24.09,33.33) | | 88.22(75.60,104.96) | | -0.22(-0.33,-0.11) | | 0.006(-0.001,0.013) | |  |
| Cameroon | 1194.58(992.38,1463.23) | | 31.41(26.09,38.47) | 4157.98(3538.08,4965.36) | | 32.25(27.44,38.51) | | 248.07(224.26,272.91) | | 0.01(-0.14,0.16) | | 0.034(0.024,0.043) | |  |
| Canada | 2633.74(2057.09,3388.98) | | 23.69(18.50,30.49) | 2317.91(1992.72,2703.05) | | 19.54(16.80,22.79) | | -11.99(-28.01,5.18) | | -0.62(-0.78,-0.46) | | -0.133(-0.144,-0.122) | |  |
| Central African Republic | 330.47(274.54,404.94) | | 31.74(26.36,38.89) | 618.13(524.77,746.62) | | 28.31(24.04,34.20) | | 87.04(76.59,99.42) | | -0.45(-0.50,-0.41) | | -0.110(-0.113,-0.107) | |  |
| Chad | 714.52(594.70,875.67) | | 34.04(28.33,41.72) | 2017.61(1721.49,2421.57) | | 32.06(27.36,38.48) | | 182.37(163.84,201.92) | | -0.19(-0.29,-0.09) | | -0.061(-0.066,-0.056) | |  |
| Chile | 1479.71(1217.69,1825.05) | | 25.84(21.26,31.87) | 1505.49(1198.13,1887.91) | | 21.28(16.93,26.68) | | 1.74(-4.20,7.98) | | -0.71(-0.89,-0.54) | | -0.147(-0.153,-0.140) | |  |
| China | 154307.37(126426.59,188055.75) | | 28.15(23.06,34.31) | 123830.39(102533.54,151222.02) | | 26.84(22.22,32.77) | | -19.75(-27.24,-11.94) | | -0.64(-0.82,-0.46) | | -0.042(-0.050,-0.034) | |  |
| Colombia | 3639.25(3023.38,4432.81) | | 25.89(21.51,31.54) | 3601.31(2886.33,4493.76) | | 17.92(14.36,22.36) | | -1.04(-8.66,6.97) | | -1.52(-1.69,-1.35) | | -0.261(-0.267,-0.256) | |  |
| Comoros | 65.36(55.54,78.28) | | 37.84(32.15,45.32) | 87.99(71.35,108.39) | | 28.40(23.03,34.99) | | 34.63(24.88,45.17) | | -1.30(-1.44,-1.16) | | -0.311(-0.320,-0.302) | |  |
| Congo | 342.79(290.26,423.88) | | 36.19(30.64,44.75) | 592.39(487.10,733.58) | | 26.74(21.98,33.11) | | 72.81(59.97,86.37) | | -1.08(-1.15,-1.01) | | -0.296(-0.306,-0.286) | |  |
| Cook Islands | 2.86(2.46,3.43) | | 37.03(31.85,44.47) | 2.02(1.75,2.39) | | 34.23(29.65,40.56) | | -29.41(-33.51,-25.24) | | -0.31(-0.41,-0.20) | | -0.079(-0.087,-0.072) | |  |
| Costa Rica | 301.62(245.39,373.68) | | 23.48(19.10,29.09) | 382.08(306.56,477.58) | | 20.07(16.11,25.09) | | 26.68(18.90,34.32) | | -0.81(-0.96,-0.66) | | -0.112(-0.119,-0.105) | |  |
| Croatia | 474.83(390.16,580.06) | | 26.17(21.50,31.97) | 208.55(162.51,274.42) | | 16.71(13.02,21.99) | | -56.08(-60.08,-52.39) | | -1.67(-1.88,-1.45) | | -0.306(-0.309,-0.303) | |  |
| Cuba | 1210.61(992.53,1485.27) | | 24.81(20.34,30.44) | 727.14(583.80,907.45) | | 20.28(16.28,25.31) | | -39.94(-44.17,-35.07) | | -1.12(-1.32,-0.91) | | -0.132(-0.142,-0.122) | |  |
| Cyprus | 50.14(39.36,63.75) | | 16.30(12.80,20.73) | 66.13(51.06,85.64) | | 13.18(10.18,17.07) | | 31.89(20.98,42.07) | | -1.16(-1.35,-0.98) | | -0.100(-0.106,-0.095) | |  |
| Czechia | 1076.09(878.41,1352.00) | | 29.00(23.67,36.44) | 587.92(455.03,751.69) | | 19.91(15.41,25.45) | | -45.37(-51.14,-39.75) | | -1.29(-1.47,-1.11) | | -0.289(-0.295,-0.282) | |  |
| Côte d'Ivoire | 1873.28(1572.11,2268.34) | | 39.60(33.24,47.96) | 3843.72(3273.94,4600.01) | | 34.28(29.20,41.03) | | 105.19(93.19,117.19) | | -0.69(-0.79,-0.59) | | -0.170(-0.179,-0.162) | |  |
| Democratic People's Republic of Korea | 2992.09(2568.46,3616.07) | | 35.87(30.79,43.35) | 3788.14(3279.15,4443.17) | | 37.66(32.60,44.17) | | 26.60(19.27,35.77) | | -0.05(-0.20,0.10) | | 0.062(0.055,0.069) | |  |
| Democratic Republic of the Congo | 4077.63(3335.46,5027.50) | | 28.46(23.28,35.10) | 8723.85(7301.76,10826.64) | | 24.18(20.23,30.00) | | 113.94(101.20,127.67) | | -0.65(-0.70,-0.60) | | -0.139(-0.140,-0.137) | |  |
| Denmark | 336.79(286.97,401.13) | | 17.65(15.04,21.02) | 251.21(189.30,325.72) | | 13.78(10.38,17.86) | | -25.41(-37.95,-12.55) | | -1.53(-1.79,-1.27) | | -0.106(-0.122,-0.091) | |  |
| Djibouti | 57.32(47.38,70.75) | | 32.69(27.02,40.36) | 161.92(133.44,200.06) | | 29.94(24.68,37.00) | | 182.51(162.26,205.37) | | -0.41(-0.47,-0.35) | | -0.077(-0.084,-0.070) | |  |
| Dominica | 6.62(5.48,8.21) | | 22.68(18.76,28.11) | 5.06(4.16,6.26) | | 19.54(16.06,24.16) | | -23.63(-29.13,-18.53) | | -0.98(-1.21,-0.75) | | -0.108(-0.115,-0.100) | |  |
| Dominican Republic | 785.48(669.44,952.92) | | 25.57(21.79,31.02) | 1291.56(1117.31,1528.33) | | 28.40(24.57,33.61) | | 64.43(53.11,76.59) | | 0.22(0.16,0.27) | | 0.098(0.090,0.107) | |  |
| Ecuador | 1406.62(1222.51,1644.96) | | 34.09(29.63,39.86) | 1866.70(1597.74,2225.09) | | 25.56(21.87,30.46) | | 32.71(25.03,40.67) | | -1.03(-1.11,-0.95) | | -0.266(-0.277,-0.254) | |  |
| Egypt | 6708.80(5715.38,8002.38) | | 30.60(26.07,36.50) | 12777.74(10671.11,15815.27) | | 30.27(25.28,37.47) | | 90.46(75.63,106.55) | | -0.04(-0.21,0.12) | | -0.004(-0.010,0.002) | |  |
| El Salvador | 622.90(533.49,733.14) | | 29.81(25.53,35.09) | 449.13(363.61,566.12) | | 17.33(14.03,21.84) | | -27.90(-34.92,-20.77) | | -2.15(-2.39,-1.91) | | -0.408(-0.417,-0.399) | |  |
| Equatorial Guinea | 50.49(42.11,61.16) | | 33.44(27.89,40.50) | 143.49(112.62,181.74) | | 20.63(16.19,26.13) | | 184.17(156.58,211.83) | | -1.78(-1.95,-1.61) | | -0.413(-0.418,-0.408) | |  |
| Eritrea | 501.62(427.71,600.40) | | 38.71(33.01,46.33) | 899.97(763.23,1083.93) | | 32.14(27.26,38.71) | | 79.41(68.28,93.18) | | -0.76(-0.83,-0.68) | | -0.214(-0.221,-0.206) | |  |
| Estonia | 199.15(170.34,237.37) | | 35.06(29.99,41.79) | 94.97(72.33,124.91) | | 24.02(18.29,31.59) | | -52.31(-60.08,-42.97) | | -1.64(-1.85,-1.44) | | -0.349(-0.365,-0.332) | |  |
| Eswatini | 63.11(50.19,82.66) | | 20.94(16.65,27.43) | 117.20(95.30,144.76) | | 23.02(18.72,28.43) | | 85.69(72.11,100.51) | | 0.32(0.12,0.52) | | 0.075(0.066,0.083) | |  |
| Ethiopia | 7166.68(5897.62,8744.44) | | 39.23(32.28,47.86) | 8842.23(7035.81,11124.39) | | 19.08(15.18,24.00) | | 23.38(10.72,37.33) | | -2.78(-2.98,-2.57) | | -0.650(-0.653,-0.647) | |  |
| Fiji | 150.34(132.16,175.98) | | 46.62(40.98,54.58) | 146.21(127.78,168.09) | | 40.99(35.82,47.12) | | -2.75(-8.39,3.62) | | -0.67(-0.76,-0.58) | | -0.195(-0.207,-0.182) | |  |
| Finland | 463.28(397.95,551.81) | | 25.52(21.92,30.40) | 333.20(274.13,410.79) | | 20.00(16.45,24.66) | | -28.08(-34.43,-21.12) | | -0.96(-1.01,-0.90) | | -0.181(-0.191,-0.171) | |  |
| France | 3347.16(2840.55,3927.92) | | 15.21(12.91,17.85) | 2559.12(2135.35,3068.20) | | 12.88(10.75,15.44) | | -23.54(-28.97,-18.75) | | -0.59(-0.66,-0.52) | | -0.072(-0.076,-0.069) | |  |
| Gabon | 119.40(97.96,147.84) | | 31.04(25.47,38.44) | 181.74(147.90,227.60) | | 24.25(19.73,30.36) | | 52.21(41.66,62.71) | | -0.97(-1.05,-0.88) | | -0.212(-0.217,-0.208) | |  |
| Gambia | 134.94(111.44,164.40) | | 35.79(29.56,43.60) | 330.10(277.60,401.33) | | 33.01(27.76,40.13) | | 144.63(129.62,162.35) | | -0.33(-0.43,-0.23) | | -0.087(-0.101,-0.073) | |  |
| Georgia | 1205.87(1048.24,1408.45) | | 56.64(49.24,66.16) | 493.40(425.06,580.86) | | 43.48(37.46,51.19) | | -59.08(-62.12,-56.01) | | -1.07(-1.29,-0.85) | | -0.375(-0.411,-0.339) | |  |
| Germany | 7630.85(6401.31,9461.21) | | 25.69(21.55,31.85) | 4459.89(3426.78,5741.62) | | 17.63(13.55,22.70) | | -41.55(-48.48,-34.12) | | -1.65(-1.90,-1.40) | | -0.261(-0.267,-0.254) | |  |
| Ghana | 3060.18(2620.86,3567.53) | | 53.31(45.66,62.15) | 7236.45(6166.18,8453.97) | | 50.60(43.11,59.11) | | 136.47(121.93,155.22) | | -0.21(-0.30,-0.13) | | -0.119(-0.142,-0.096) | |  |
| Greece | 885.94(725.83,1083.34) | | 23.57(19.31,28.82) | 485.44(380.67,607.22) | | 17.44(13.67,21.81) | | -45.21(-49.26,-40.80) | | -1.03(-1.13,-0.93) | | -0.189(-0.201,-0.176) | |  |
| Greenland | 8.60(7.03,10.65) | | 32.50(26.58,40.24) | 4.41(3.53,5.55) | | 21.63(17.29,27.23) | | -48.71(-54.25,-43.82) | | -2.27(-2.66,-1.89) | | -0.357(-0.376,-0.338) | |  |
| Grenada | 10.87(9.48,12.98) | | 32.60(28.43,38.92) | 8.91(7.47,10.95) | | 22.04(18.47,27.09) | | -18.04(-24.63,-11.80) | | -1.52(-1.65,-1.39) | | -0.345(-0.351,-0.339) | |  |
| Guam | 18.37(15.55,22.09) | | 28.96(24.52,34.83) | 19.66(16.96,23.27) | | 35.47(30.60,41.97) | | 7.04(-0.43,15.25) | | 0.38(0.25,0.51) | | 0.208(0.203,0.213) | |  |
| Guatemala | 930.11(819.64,1072.05) | | 31.49(27.75,36.29) | 1504.48(1284.62,1772.78) | | 22.10(18.87,26.04) | | 61.75(51.62,71.52) | | -1.55(-1.72,-1.39) | | -0.309(-0.316,-0.302) | |  |
| Guinea | 746.75(638.46,899.67) | | 36.35(31.08,43.79) | 1857.52(1574.36,2227.53) | | 35.95(30.47,43.11) | | 148.75(132.19,166.98) | | -0.01(-0.06,0.04) | | -0.015(-0.024,-0.006) | |  |
| Guinea-Bissau | 175.58(152.37,210.50) | | 47.35(41.09,56.76) | 345.25(300.32,406.19) | | 40.92(35.59,48.14) | | 96.63(85.46,108.86) | | -0.54(-0.59,-0.49) | | -0.192(-0.208,-0.176) | |  |
| Guyana | 131.78(116.24,152.42) | | 38.72(34.15,44.78) | 90.76(79.37,106.31) | | 29.21(25.54,34.21) | | -31.13(-34.90,-27.26) | | -1.35(-1.55,-1.16) | | -0.320(-0.330,-0.309) | |  |
| Haiti | 901.43(778.96,1057.25) | | 37.03(32.00,43.43) | 1682.75(1466.71,1969.49) | | 30.65(26.72,35.88) | | 86.68(75.55,98.45) | | -0.70(-0.87,-0.53) | | -0.205(-0.212,-0.198) | |  |
| Honduras | 408.17(344.93,493.57) | | 23.64(19.98,28.59) | 719.75(568.69,920.78) | | 16.37(12.94,20.95) | | 76.34(62.60,91.33) | | -1.54(-1.67,-1.41) | | -0.238(-0.242,-0.235) | |  |
| Hungary | 1592.59(1381.12,1901.25) | | 43.09(37.37,51.44) | 589.65(474.83,742.65) | | 21.43(17.26,26.99) | | -62.98(-67.62,-58.69) | | -2.44(-2.62,-2.26) | | -0.712(-0.720,-0.705) | |  |
| Iceland | 21.20(16.51,26.81) | | 20.41(15.89,25.80) | 17.84(13.42,23.05) | | 14.91(11.22,19.26) | | -15.83(-22.84,-9.70) | | -1.48(-1.65,-1.30) | | -0.178(-0.185,-0.171) | |  |
| India | 80280.47(65079.60,99531.18) | | 23.54(19.08,29.19) | 131103.66(106504.02,162808.88) | | 21.51(17.48,26.71) | | 63.31(55.13,70.84) | | -0.52(-0.63,-0.40) | | -0.068(-0.075,-0.061) | |  |
| Indonesia | 36529.54(30408.17,43490.49) | | 46.81(38.96,55.73) | 41993.25(35744.46,50076.31) | | 36.88(31.39,43.98) | | 14.96(7.91,21.85) | | -0.96(-1.11,-0.81) | | -0.313(-0.327,-0.300) | |  |
| Iran (Islamic Republic of) | 6715.95(5490.20,8393.46) | | 30.92(25.28,38.65) | 8824.56(7227.58,10987.13) | | 25.43(20.83,31.66) | | 31.40(20.65,41.92) | | -0.70(-0.77,-0.64) | | -0.178(-0.185,-0.171) | |  |
| Iraq | 2942.53(2522.72,3493.73) | | 40.95(35.11,48.62) | 5834.45(4966.44,6968.26) | | 33.46(28.48,39.96) | | 98.28(85.87,111.20) | | -0.70(-0.87,-0.52) | | -0.216(-0.232,-0.200) | |  |
| Ireland | 271.25(215.69,339.98) | | 19.77(15.72,24.79) | 207.12(157.08,270.84) | | 13.24(10.04,17.31) | | -23.65(-31.98,-16.64) | | -1.83(-2.03,-1.63) | | -0.208(-0.214,-0.203) | |  |
| Israel | 345.00(263.28,440.11) | | 18.05(13.78,23.03) | 434.77(320.44,573.20) | | 13.08(9.64,17.25) | | 26.02(15.14,35.75) | | -0.93(-1.42,-0.45) | | -0.149(-0.166,-0.131) | |  |
| Italy | 4199.26(3187.44,5561.49) | | 19.67(14.93,26.05) | 1865.88(1410.89,2429.14) | | 11.81(8.93,15.38) | | -55.57(-60.76,-51.27) | | -1.90(-2.03,-1.78) | | -0.254(-0.258,-0.250) | |  |
| Jamaica | 228.06(189.17,282.48) | | 23.21(19.25,28.74) | 262.02(220.76,323.70) | | 21.96(18.50,27.13) | | 14.89(7.37,22.51) | | -0.66(-0.82,-0.50) | | -0.043(-0.056,-0.029) | |  |
| Japan | 9525.96(7332.15,12220.42) | | 21.26(16.36,27.27) | 7117.58(5501.86,9122.53) | | 21.96(16.98,28.15) | | -25.28(-30.04,-20.39) | | 0.24(0.02,0.45) | | 0.019(0.008,0.031) | |  |
| Jordan | 473.43(376.81,610.75) | | 30.80(24.52,39.74) | 1352.74(1058.53,1749.55) | | 25.20(19.72,32.59) | | 185.73(164.33,210.91) | | -0.93(-1.09,-0.76) | | -0.170(-0.178,-0.161) | |  |
| Kazakhstan | 3358.48(2856.81,3984.45) | | 49.47(42.08,58.69) | 2717.01(2303.58,3282.99) | | 38.99(33.05,47.11) | | -19.10(-24.66,-12.11) | | -1.13(-1.50,-0.76) | | -0.303(-0.340,-0.266) | |  |
| Kenya | 2410.17(1937.80,3046.29) | | 27.52(22.13,34.78) | 5146.17(4168.15,6441.08) | | 23.77(19.25,29.75) | | 113.52(102.01,123.67) | | -0.58(-0.71,-0.44) | | -0.121(-0.129,-0.112) | |  |
| Kiribati | 29.82(26.44,33.58) | | 97.68(86.60,110.00) | 48.63(43.99,53.46) | | 97.80(88.46,107.52) | | 63.06(52.35,73.99) | | -0.27(-0.40,-0.15) | | 0.048(-0.006,0.101) | |  |
| Kuwait | 251.55(203.41,318.92) | | 29.77(24.07,37.74) | 620.97(494.95,787.82) | | 29.25(23.32,37.11) | | 146.85(128.02,168.08) | | -0.09(-0.32,0.15) | | -0.016(-0.030,-0.003) | |  |
| Kyrgyzstan | 854.65(741.65,1008.55) | | 47.39(41.12,55.92) | 776.19(656.79,946.20) | | 28.52(24.13,34.77) | | -9.18(-16.39,-1.80) | | -2.26(-2.51,-2.01) | | -0.613(-0.630,-0.597) | |  |
| Lao People's Democratic Republic | 555.82(471.80,673.83) | | 35.97(30.54,43.61) | 1056.98(916.91,1246.08) | | 32.95(28.58,38.84) | | 90.17(78.41,104.57) | | -0.45(-0.55,-0.36) | | -0.086(-0.094,-0.077) | |  |
| Latvia | 307.22(254.65,375.00) | | 32.20(26.69,39.31) | 125.06(98.19,157.12) | | 23.22(18.23,29.17) | | -59.29(-62.66,-56.23) | | -1.38(-1.53,-1.23) | | -0.284(-0.293,-0.275) | |  |
| Lebanon | 391.71(330.09,476.34) | | 33.98(28.63,41.32) | 731.43(609.09,908.38) | | 31.52(26.25,39.15) | | 86.73(71.15,103.83) | | -0.24(-0.41,-0.07) | | -0.077(-0.084,-0.070) | |  |
| Lesotho | 102.13(79.43,133.37) | | 18.93(14.72,24.72) | 189.15(157.69,235.71) | | 22.74(18.96,28.34) | | 85.20(66.41,106.70) | | 0.89(0.77,1.01) | | 0.126(0.121,0.132) | |  |
| Liberia | 351.20(296.68,427.89) | | 38.07(32.16,46.38) | 712.36(597.50,865.33) | | 31.73(26.61,38.54) | | 102.84(90.94,114.51) | | -0.59(-0.73,-0.44) | | -0.209(-0.224,-0.194) | |  |
| Libya | 484.66(409.99,587.03) | | 28.85(24.40,34.94) | 996.28(848.31,1185.33) | | 33.20(28.27,39.50) | | 105.56(87.05,128.05) | | 0.58(0.52,0.65) | | 0.142(0.136,0.147) | |  |
| Lithuania | 456.26(369.43,572.57) | | 32.75(26.51,41.09) | 214.69(171.55,275.18) | | 26.67(21.31,34.18) | | -52.94(-56.59,-49.75) | | -0.77(-0.89,-0.64) | | -0.197(-0.206,-0.187) | |  |
| Luxembourg | 32.28(26.03,39.58) | | 21.88(17.64,26.82) | 26.91(20.44,34.61) | | 12.20(9.27,15.69) | | -16.63(-26.79,-7.78) | | -2.59(-2.91,-2.26) | | -0.315(-0.321,-0.309) | |  |
| Madagascar | 2711.56(2387.92,3134.03) | | 59.86(52.72,69.19) | 5203.20(4532.07,6070.90) | | 44.43(38.70,51.84) | | 91.89(80.38,104.48) | | -1.07(-1.14,-1.00) | | -0.496(-0.516,-0.476) | |  |
| Malawi | 1121.20(935.24,1389.93) | | 30.01(25.03,37.20) | 2097.73(1745.44,2588.49) | | 25.63(21.33,31.63) | | 87.10(76.62,97.89) | | -0.62(-0.72,-0.52) | | -0.133(-0.137,-0.128) | |  |
| Malaysia | 2863.91(2443.80,3466.88) | | 38.59(32.93,46.71) | 4538.27(3824.35,5485.30) | | 32.64(27.51,39.46) | | 58.46(48.15,69.86) | | -0.63(-0.82,-0.43) | | -0.206(-0.218,-0.195) | |  |
| Maldives | 36.12(31.25,41.91) | | 44.45(38.46,51.57) | 78.29(64.41,95.51) | | 30.09(24.76,36.71) | | 116.76(90.61,144.11) | | -1.99(-2.39,-1.59) | | -0.477(-0.498,-0.455) | |  |
| Mali | 1022.64(857.58,1246.51) | | 34.25(28.72,41.75) | 2304.09(1908.51,2863.10) | | 25.87(21.43,32.14) | | 125.31(111.25,140.64) | | -1.02(-1.10,-0.94) | | -0.269(-0.272,-0.267) | |  |
| Malta | 28.86(22.89,35.86) | | 20.92(16.59,25.99) | 20.16(15.73,25.62) | | 15.06(11.75,19.14) | | -30.14(-35.77,-24.83) | | -1.35(-1.51,-1.19) | | -0.187(-0.192,-0.183) | |  |
| Marshall Islands | 9.00(7.89,10.37) | | 52.49(46.00,60.45) | 15.55(13.93,17.56) | | 65.56(58.71,74.04) | | 72.73(62.72,83.64) | | 0.84(0.71,0.97) | | 0.394(0.377,0.412) | |  |
| Mauritania | 332.12(280.46,402.04) | | 43.20(36.48,52.29) | 485.12(398.54,612.30) | | 28.42(23.35,35.87) | | 46.07(36.00,56.89) | | -1.53(-1.63,-1.43) | | -0.476(-0.482,-0.469) | |  |
| Mauritius | 198.59(170.85,234.36) | | 39.95(34.37,47.14) | 129.72(109.75,155.31) | | 28.50(24.11,34.12) | | -34.68(-38.98,-30.35) | | -1.52(-1.69,-1.34) | | -0.367(-0.380,-0.354) | |  |
| Mexico | 9175.94(7439.99,11625.57) | | 25.73(20.86,32.60) | 10306.39(8331.99,12902.04) | | 20.01(16.17,25.05) | | 12.32(6.16,18.08) | | -1.07(-1.25,-0.89) | | -0.185(-0.191,-0.179) | |  |
| Micronesia (Federated States of) | 23.85(20.53,27.40) | | 59.53(51.23,68.39) | 25.27(22.35,28.74) | | 59.51(52.64,67.68) | | 5.96(-0.25,13.19) | | 0.00(-0.05,0.05) | | -0.018(-0.045,0.009) | |  |
| Monaco | 1.90(1.50,2.44) | | 20.79(16.36,26.66) | 1.50(1.15,1.92) | | 16.07(12.38,20.59) | | -21.28(-26.28,-16.69) | | -1.18(-1.30,-1.06) | | -0.155(-0.159,-0.151) | |  |
| Mongolia | 374.10(322.00,451.68) | | 42.33(36.44,51.11) | 609.06(524.19,723.41) | | 48.26(41.53,57.32) | | 62.81(51.54,76.45) | | 0.26(0.05,0.47) | | 0.206(0.176,0.237) | |  |
| Montenegro | 79.94(67.04,96.75) | | 31.85(26.71,38.54) | 50.24(41.53,62.53) | | 24.42(20.19,30.40) | | -37.15(-41.51,-33.17) | | -1.30(-1.49,-1.12) | | -0.243(-0.254,-0.232) | |  |
| Morocco | 3479.24(2942.37,4178.04) | | 33.46(28.30,40.19) | 4087.83(3390.85,4954.19) | | 27.84(23.10,33.74) | | 17.49(9.03,24.84) | | -0.75(-0.91,-0.59) | | -0.160(-0.174,-0.146) | |  |
| Mozambique | 1423.78(1172.83,1782.52) | | 30.02(24.73,37.58) | 3767.23(3220.52,4630.26) | | 31.33(26.79,38.51) | | 164.59(145.64,186.95) | | 0.41(0.28,0.54) | | 0.046(0.039,0.053) | |  |
| Myanmar | 6454.27(5548.47,7703.91) | | 37.60(32.32,44.88) | 7207.22(6244.18,8616.44) | | 32.06(27.77,38.33) | | 11.67(4.17,19.63) | | -0.63(-0.85,-0.42) | | -0.155(-0.169,-0.140) | |  |
| Namibia | 137.31(109.77,175.74) | | 24.55(19.63,31.43) | 217.50(172.51,274.25) | | 20.81(16.51,26.24) | | 58.40(47.14,71.47) | | -0.83(-0.99,-0.66) | | -0.118(-0.124,-0.111) | |  |
| Nauru | 3.14(2.77,3.61) | | 77.74(68.44,89.22) | 3.71(3.29,4.13) | | 79.75(70.74,88.78) | | 18.10(11.19,25.31) | | 0.00(-0.21,0.22) | | 0.039(-0.028,0.106) | |  |
| Nepal | 1626.23(1318.15,2024.84) | | 22.26(18.04,27.72) | 2317.53(1827.92,2947.59) | | 17.29(13.63,21.98) | | 42.51(32.59,52.79) | | -0.98(-1.07,-0.89) | | -0.160(-0.164,-0.156) | |  |
| Netherlands | 1335.06(1056.68,1702.29) | | 22.14(17.52,28.23) | 797.71(602.64,1034.84) | | 15.11(11.41,19.60) | | -40.25(-45.04,-36.00) | | -1.86(-2.10,-1.62) | | -0.233(-0.240,-0.226) | |  |
| New Zealand | 237.87(176.99,314.26) | | 17.22(12.81,22.75) | 237.56(174.43,315.46) | | 13.19(9.68,17.51) | | -0.13(-6.73,7.46) | | -1.03(-1.27,-0.79) | | -0.129(-0.133,-0.125) | |  |
| Nicaragua | 321.92(262.01,402.27) | | 21.81(17.75,27.25) | 479.45(375.46,607.80) | | 16.85(13.19,21.36) | | 48.93(37.79,59.56) | | -1.07(-1.20,-0.94) | | -0.157(-0.163,-0.152) | |  |
| Niger | 973.18(823.10,1193.00) | | 34.96(29.57,42.86) | 2427.69(2010.98,2991.23) | | 27.22(22.55,33.54) | | 149.46(132.06,163.79) | | -1.03(-1.10,-0.95) | | -0.251(-0.255,-0.247) | |  |
| Nigeria | 9690.39(7777.55,12204.96) | | 28.38(22.78,35.75) | 21419.34(17206.10,27298.32) | | 23.82(19.13,30.35) | | 121.04(109.25,132.14) | | -0.62(-0.73,-0.51) | | -0.147(-0.153,-0.140) | |  |
| Niue | 0.41(0.36,0.48) | | 51.22(44.73,59.34) | 0.24(0.21,0.28) | | 42.18(36.89,49.21) | | -41.91(-45.35,-38.35) | | -0.81(-0.92,-0.69) | | -0.313(-0.333,-0.293) | |  |
| North Macedonia | 297.43(258.90,353.39) | | 37.46(32.60,44.50) | 226.25(193.39,275.37) | | 29.58(25.29,36.01) | | -23.93(-28.77,-18.65) | | -1.07(-1.22,-0.93) | | -0.254(-0.264,-0.245) | |  |
| Northern Mariana Islands | 9.21(7.83,10.92) | | 39.31(33.44,46.63) | 5.22(4.46,6.21) | | 31.66(27.06,37.64) | | -43.30(-47.39,-39.15) | | -1.00(-1.27,-0.73) | | -0.226(-0.281,-0.171) | |  |
| Norway | 347.69(256.83,462.56) | | 21.73(16.05,28.91) | 237.71(175.88,311.80) | | 13.39(9.91,17.57) | | -31.63(-38.08,-25.29) | | -2.09(-2.27,-1.91) | | -0.268(-0.276,-0.261) | |  |
| Oman | 300.99(257.25,360.88) | | 36.28(31.01,43.50) | 762.61(636.33,936.50) | | 32.95(27.50,40.47) | | 153.37(132.67,175.02) | | -0.18(-0.28,-0.07) | | -0.096(-0.122,-0.071) | |  |
| Pakistan | 11691.46(9556.40,14281.83) | | 28.65(23.42,35.00) | 30595.79(25996.01,36953.97) | | 30.93(26.28,37.36) | | 161.69(144.68,181.43) | | 0.17(0.04,0.30) | | 0.077(0.065,0.088) | |  |
| Palau | 3.92(3.44,4.52) | | 56.22(49.33,64.77) | 3.88(3.45,4.42) | | 65.81(58.49,75.01) | | -1.16(-7.74,6.84) | | 0.37(0.20,0.53) | | 0.274(0.234,0.315) | |  |
| Palestine | 193.35(157.46,248.77) | | 25.19(20.52,32.41) | 469.78(382.60,595.91) | | 21.51(17.52,27.29) | | 142.97(124.35,159.18) | | -0.73(-0.81,-0.64) | | -0.118(-0.127,-0.109) | |  |
| Panama | 228.29(187.81,278.76) | | 22.57(18.57,27.56) | 296.61(235.45,372.30) | | 17.97(14.27,22.56) | | 29.93(20.89,38.03) | | -0.88(-0.94,-0.81) | | -0.152(-0.154,-0.150) | |  |
| Papua New Guinea | 332.39(266.30,416.40) | | 20.08(16.09,25.16) | 771.01(614.96,960.83) | | 18.02(14.37,22.45) | | 131.96(118.06,146.22) | | -0.48(-0.59,-0.38) | | -0.068(-0.075,-0.061) | |  |
| Paraguay | 380.74(315.60,458.95) | | 24.29(20.14,29.28) | 565.55(456.23,700.80) | | 18.48(14.91,22.91) | | 48.54(38.05,58.90) | | -1.20(-1.34,-1.05) | | -0.189(-0.192,-0.185) | |  |
| Peru | 2721.25(2351.63,3236.28) | | 30.67(26.51,36.48) | 3568.64(2991.05,4287.85) | | 24.01(20.13,28.85) | | 31.14(22.45,40.09) | | -0.87(-0.99,-0.76) | | -0.213(-0.219,-0.207) | |  |
| Philippines | 5310.34(4173.44,6820.78) | | 20.49(16.10,26.32) | 15868.28(13512.64,18929.02) | | 33.58(28.60,40.06) | | 198.82(167.56,236.99) | | 2.23(1.92,2.54) | | 0.429(0.420,0.437) | |  |
| Poland | 4505.02(3713.07,5536.31) | | 31.19(25.70,38.33) | 2185.38(1745.20,2782.15) | | 18.06(14.42,23.00) | | -51.49(-55.15,-47.56) | | -2.03(-2.38,-1.68) | | -0.421(-0.430,-0.411) | |  |
| Portugal | 884.31(744.62,1060.72) | | 23.36(19.67,28.02) | 336.07(255.36,443.04) | | 11.39(8.65,15.01) | | -62.00(-67.16,-56.56) | | -2.86(-3.09,-2.63) | | -0.385(-0.393,-0.377) | |  |
| Puerto Rico | 299.82(243.67,370.76) | | 21.20(17.23,26.21) | 181.83(141.79,233.58) | | 17.58(13.71,22.58) | | -39.35(-43.68,-35.19) | | -0.85(-0.97,-0.73) | | -0.118(-0.128,-0.109) | |  |
| Qatar | 99.67(85.17,118.19) | | 42.15(36.02,49.98) | 498.56(391.43,643.70) | | 30.17(23.69,38.95) | | 400.19(342.71,463.16) | | -1.58(-1.76,-1.41) | | -0.390(-0.413,-0.366) | |  |
| Republic of Korea | 8716.91(7298.04,10493.56) | | 41.41(34.67,49.85) | 3690.24(2861.91,4770.52) | | 23.06(17.89,29.81) | | -57.67(-62.92,-51.69) | | -2.71(-3.03,-2.40) | | -0.600(-0.619,-0.581) | |  |
| Republic of Moldova | 587.88(492.42,708.49) | | 33.73(28.25,40.65) | 347.60(283.79,430.45) | | 28.03(22.88,34.71) | | -40.87(-45.18,-36.63) | | -0.62(-0.73,-0.51) | | -0.178(-0.196,-0.160) | |  |
| Romania | 2584.10(2184.85,3156.60) | | 29.75(25.15,36.34) | 1186.83(967.32,1472.82) | | 22.02(17.95,27.33) | | -54.07(-57.11,-50.54) | | -1.01(-1.08,-0.94) | | -0.248(-0.255,-0.242) | |  |
| Russian Federation | 21433.77(17617.99,26350.18) | | 36.85(30.29,45.30) | 16502.20(13823.26,20245.95) | | 35.51(29.74,43.56) | | -23.01(-26.70,-18.93) | | -0.16(-0.28,-0.04) | | -0.020(-0.051,0.011) | |  |
| Rwanda | 1458.98(1256.71,1705.86) | | 53.24(45.86,62.24) | 1414.72(1160.22,1742.82) | | 24.94(20.46,30.73) | | -3.03(-12.73,7.07) | | -3.16(-3.50,-2.81) | | -0.904(-0.922,-0.887) | |  |
| Saint Kitts and Nevis | 8.30(7.26,9.58) | | 48.03(42.03,55.41) | 5.33(4.36,6.61) | | 23.45(19.19,29.07) | | -35.79(-42.58,-27.53) | | -2.97(-3.24,-2.69) | | -0.806(-0.828,-0.783) | |  |
| Saint Lucia | 16.16(13.81,19.90) | | 28.73(24.55,35.37) | 14.85(12.24,18.34) | | 22.46(18.52,27.75) | | -8.14(-15.61,-0.64) | | -1.07(-1.17,-0.97) | | -0.203(-0.207,-0.199) | |  |
| Saint Vincent and the Grenadines | 13.67(11.89,16.23) | | 29.76(25.88,35.33) | 9.02(7.60,11.01) | | 21.82(18.39,26.63) | | -34.05(-39.14,-28.72) | | -1.40(-1.54,-1.27) | | -0.259(-0.266,-0.252) | |  |
| Samoa | 26.76(23.01,31.69) | | 39.96(34.36,47.32) | 38.83(34.30,44.54) | | 48.33(42.68,55.43) | | 45.12(35.65,55.50) | | 0.52(0.34,0.70) | | 0.272(0.263,0.281) | |  |
| San Marino | 1.47(1.10,1.88) | | 15.64(11.73,20.06) | 1.27(0.96,1.64) | | 14.15(10.66,18.27) | | -13.66(-18.25,-8.55) | | -0.46(-0.58,-0.35) | | -0.048(-0.050,-0.045) | |  |
| Sao Tome and Principe | 17.31(14.58,21.19) | | 40.33(33.96,49.37) | 42.04(36.08,50.10) | | 46.25(39.70,55.13) | | 142.80(123.28,163.29) | | 0.35(0.17,0.53) | | 0.178(0.146,0.210) | |  |
| Saudi Arabia | 1742.94(1467.34,2166.26) | | 26.23(22.08,32.60) | 5352.41(4591.55,6411.82) | | 28.90(24.79,34.62) | | 207.09(177.99,237.31) | | 0.39(0.34,0.45) | | 0.093(0.087,0.100) | |  |
| Senegal | 1059.11(879.36,1308.14) | | 38.38(31.87,47.41) | 2063.51(1712.38,2538.39) | | 32.00(26.56,39.37) | | 94.83(85.33,105.69) | | -0.61(-0.65,-0.57) | | -0.197(-0.203,-0.191) | |  |
| Serbia | 1191.04(1017.04,1450.26) | | 33.17(28.32,40.38) | 618.29(507.88,766.48) | | 20.86(17.13,25.86) | | -48.09(-52.35,-43.71) | | -1.84(-1.99,-1.69) | | -0.401(-0.406,-0.395) | |  |
| Seychelles | 11.48(9.83,13.64) | | 36.76(31.48,43.68) | 12.24(10.45,14.51) | | 31.88(27.21,37.80) | | 6.62(0.44,14.58) | | -0.75(-0.84,-0.65) | | -0.152(-0.162,-0.142) | |  |
| Sierra Leone | 610.42(509.38,750.64) | | 38.21(31.89,46.99) | 1328.56(1132.18,1599.54) | | 35.62(30.36,42.89) | | 117.65(101.65,132.99) | | -0.29(-0.34,-0.24) | | -0.076(-0.084,-0.068) | |  |
| Singapore | 419.30(332.07,528.46) | | 27.78(22.00,35.01) | 394.02(292.87,525.92) | | 20.48(15.22,27.34) | | -6.03(-17.28,6.63) | | -1.54(-1.85,-1.22) | | -0.235(-0.244,-0.226) | |  |
| Slovakia | 608.49(504.13,745.47) | | 29.73(24.63,36.42) | 389.68(314.08,491.53) | | 22.77(18.35,28.72) | | -35.96(-40.74,-30.93) | | -0.86(-1.10,-0.62) | | -0.222(-0.225,-0.220) | |  |
| Slovenia | 181.67(146.48,225.57) | | 23.71(19.11,29.43) | 88.28(67.64,116.12) | | 15.50(11.88,20.39) | | -51.40(-56.33,-47.23) | | -1.65(-1.92,-1.39) | | -0.264(-0.269,-0.259) | |  |
| Solomon Islands | 63.64(54.53,73.97) | | 49.62(42.52,57.67) | 170.13(152.89,193.88) | | 62.20(55.90,70.88) | | 167.31(148.88,188.08) | | 0.89(0.74,1.03) | | 0.381(0.365,0.397) | |  |
| Somalia | 1182.45(994.37,1424.74) | | 40.78(34.30,49.14) | 2543.78(2166.26,3052.58) | | 30.68(26.13,36.82) | | 115.13(100.87,130.21) | | -0.81(-0.94,-0.67) | | -0.324(-0.335,-0.312) | |  |
| South Africa | 5997.00(4893.93,7323.35) | | 38.11(31.10,46.54) | 5890.01(4768.14,7351.40) | | 24.29(19.66,30.32) | | -1.78(-9.66,5.87) | | -2.05(-2.36,-1.74) | | -0.443(-0.453,-0.433) | |  |
| South Sudan | 683.45(563.43,843.20) | | 29.61(24.41,36.53) | 843.28(686.49,1064.69) | | 23.43(19.07,29.58) | | 23.39(14.00,33.15) | | -0.81(-0.86,-0.76) | | -0.197(-0.201,-0.193) | |  |
| Spain | 2956.05(2433.80,3632.66) | | 19.93(16.41,24.50) | 1598.16(1273.21,2030.72) | | 12.87(10.25,16.36) | | -45.94(-52.82,-39.53) | | -1.47(-1.52,-1.43) | | -0.227(-0.233,-0.222) | |  |
| Sri Lanka | 2153.00(1793.31,2633.47) | | 29.12(24.25,35.61) | 1912.62(1571.91,2369.90) | | 23.72(19.49,29.39) | | -11.17(-16.14,-5.66) | | -0.95(-1.05,-0.85) | | -0.177(-0.183,-0.171) | |  |
| Sudan | 2721.19(2318.92,3219.45) | | 35.71(30.43,42.25) | 5458.68(4540.82,6551.80) | | 29.53(24.56,35.44) | | 100.60(86.12,117.79) | | -0.68(-0.76,-0.61) | | -0.197(-0.201,-0.192) | |  |
| Suriname | 47.93(41.34,57.33) | | 29.43(25.38,35.21) | 57.67(50.04,68.82) | | 26.86(23.31,32.06) | | 20.31(13.23,28.33) | | -0.52(-0.59,-0.45) | | -0.088(-0.093,-0.082) | |  |
| Sweden | 503.94(384.95,651.01) | | 17.17(13.11,22.18) | 546.84(407.07,725.19) | | 16.87(12.56,22.37) | | 8.51(-0.23,17.77) | | -0.19(-0.36,-0.02) | | -0.002(-0.009,0.005) | |  |
| Switzerland | 434.64(340.45,556.66) | | 16.50(12.92,21.13) | 319.65(238.81,427.13) | | 11.51(8.60,15.38) | | -26.46(-34.76,-19.13) | | -1.58(-1.79,-1.37) | | -0.163(-0.168,-0.157) | |  |
| Syrian Arab Republic | 2688.86(2350.82,3087.51) | | 56.05(49.00,64.36) | 1856.34(1591.62,2203.08) | | 36.50(31.29,43.32) | | -30.96(-35.76,-25.77) | | -1.50(-1.63,-1.38) | | -0.621(-0.634,-0.609) | |  |
| Taiwan (Province of China) | 2959.59(2469.07,3602.12) | | 32.08(26.76,39.04) | 2031.85(1644.47,2508.76) | | 26.93(21.80,33.25) | | -31.35(-37.16,-25.33) | | -0.78(-0.87,-0.69) | | -0.166(-0.169,-0.163) | |  |
| Tajikistan | 686.11(575.00,842.73) | | 32.45(27.19,39.85) | 1075.28(879.73,1336.64) | | 25.78(21.09,32.04) | | 56.72(46.78,66.54) | | -1.13(-1.37,-0.90) | | -0.216(-0.225,-0.207) | |  |
| Thailand | 8343.28(7077.78,9958.93) | | 32.17(27.29,38.40) | 6880.85(5958.65,8091.80) | | 32.46(28.11,38.17) | | -17.53(-22.99,-11.64) | | -0.23(-0.42,-0.03) | | 0.015(0.008,0.022) | |  |
| Timor-Leste | 79.96(64.72,98.83) | | 25.12(20.33,31.05) | 136.42(114.71,167.15) | | 23.88(20.08,29.26) | | 70.61(59.73,84.13) | | -0.28(-0.35,-0.21) | | -0.035(-0.043,-0.027) | |  |
| Togo | 519.62(436.41,638.76) | | 37.90(31.83,46.60) | 1178.23(989.15,1413.06) | | 35.02(29.40,42.00) | | 126.75(113.62,142.82) | | -0.34(-0.40,-0.27) | | -0.101(-0.109,-0.092) | |  |
| Tokelau | 0.24(0.21,0.28) | | 40.65(35.45,47.57) | 0.19(0.17,0.22) | | 38.47(33.52,45.27) | | -19.29(-24.31,-13.77) | | -0.29(-0.41,-0.16) | | -0.071(-0.097,-0.045) | |  |
| Tonga | 9.43(7.82,11.59) | | 25.55(21.20,31.41) | 10.28(8.66,12.51) | | 26.42(22.26,32.17) | | 8.98(3.00,16.23) | | 0.11(0.04,0.18) | | 0.029(0.026,0.032) | |  |
| Trinidad and Tobago | 163.36(140.73,194.00) | | 32.58(28.07,38.70) | 123.81(101.89,153.84) | | 24.87(20.47,30.91) | | -24.21(-30.78,-16.79) | | -1.28(-1.44,-1.12) | | -0.253(-0.262,-0.245) | |  |
| Tunisia | 851.48(700.42,1073.09) | | 24.75(20.36,31.20) | 1113.42(922.33,1389.10) | | 25.59(21.19,31.92) | | 30.76(20.46,42.78) | | 0.05(-0.01,0.11) | | 0.034(0.029,0.039) | |  |
| Turkey | 9072.19(7711.37,11074.70) | | 37.93(32.24,46.30) | 7851.83(6339.51,9898.33) | | 24.65(19.90,31.07) | | -13.45(-21.40,-5.26) | | -1.89(-2.05,-1.72) | | -0.443(-0.455,-0.430) | |  |
| Turkmenistan | 547.24(465.62,666.76) | | 35.65(30.33,43.44) | 1065.51(914.86,1236.28) | | 51.23(43.99,59.44) | | 94.71(77.36,113.02) | | 1.27(1.08,1.46) | | 0.484(0.456,0.513) | |  |
| Tuvalu | 2.00(1.75,2.31) | | 55.35(48.50,64.08) | 2.66(2.38,3.04) | | 53.55(47.99,61.20) | | 33.22(24.15,43.57) | | -0.29(-0.38,-0.19) | | -0.052(-0.074,-0.030) | |  |
| Uganda | 1821.27(1490.92,2309.10) | | 28.39(23.24,36.00) | 4153.24(3405.88,5147.42) | | 24.15(19.80,29.93) | | 128.04(115.94,141.09) | | -0.83(-1.02,-0.63) | | -0.127(-0.136,-0.118) | |  |
| Ukraine | 6912.97(5555.63,8511.90) | | 36.39(29.25,44.81) | 6213.22(5210.08,7617.43) | | 45.07(37.80,55.26) | | -10.12(-18.86,-1.31) | | 0.69(0.50,0.89) | | 0.299(0.265,0.334) | |  |
| United Arab Emirates | 388.29(323.12,476.30) | | 40.62(33.80,49.83) | 1804.70(1440.66,2247.96) | | 44.92(35.86,55.96) | | 364.78(299.14,435.65) | | 0.19(-0.24,0.61) | | 0.150(0.131,0.169) | |  |
| United Kingdom | 3783.38(2948.66,4741.33) | | 18.10(14.11,22.69) | 3035.76(2374.23,3828.43) | | 13.96(10.92,17.60) | | -19.76(-23.79,-15.93) | | -1.25(-1.42,-1.08) | | -0.134(-0.138,-0.130) | |  |
| United Republic of Tanzania | 2852.16(2376.33,3456.30) | | 29.44(24.53,35.67) | 6521.86(5370.95,8039.57) | | 27.95(23.02,34.45) | | 128.66(113.26,144.25) | | -0.18(-0.23,-0.13) | | -0.046(-0.049,-0.044) | |  |
| United States of America | 21201.85(16350.73,27347.12) | | 20.75(16.00,26.77) | 20044.67(15466.15,26123.57) | | 18.01(13.90,23.47) | | -5.46(-9.58,-1.27) | | -0.68(-0.81,-0.54) | | -0.078(-0.086,-0.071) | |  |
| United States Virgin Islands | 9.18(7.50,11.28) | | 23.17(18.94,28.48) | 4.42(3.50,5.59) | | 19.11(15.14,24.16) | | -51.83(-55.00,-48.38) | | -1.04(-1.22,-0.85) | | -0.130(-0.139,-0.120) | |  |
| Uruguay | 429.74(365.38,522.22) | | 37.84(32.17,45.98) | 253.18(205.54,313.73) | | 21.17(17.18,26.23) | | -41.08(-46.72,-35.80) | | -2.32(-2.57,-2.07) | | -0.545(-0.554,-0.535) | |  |
| Uzbekistan | 3089.71(2613.51,3853.30) | | 36.00(30.45,44.89) | 4838.05(4080.27,5911.17) | | 35.22(29.70,43.03) | | 56.59(45.70,68.87) | | -0.22(-0.32,-0.12) | | -0.000(-0.014,0.013) | |  |
| Vanuatu | 30.68(26.65,35.43) | | 52.37(45.50,60.48) | 79.04(70.13,89.34) | | 63.42(56.27,71.68) | | 157.65(139.90,176.71) | | 0.63(0.53,0.74) | | 0.347(0.327,0.367) | |  |
| Venezuela (Bolivarian Republic of) | 2140.56(1781.39,2610.45) | | 26.73(22.24,32.59) | 2196.02(1819.92,2653.61) | | 23.46(19.44,28.34) | | 2.59(-3.86,10.06) | | -0.65(-0.84,-0.47) | | -0.102(-0.109,-0.095) | |  |
| Viet Nam | 8545.47(7174.47,10499.19) | | 29.97(25.16,36.82) | 11939.62(10123.99,14402.30) | | 31.10(26.37,37.52) | | 39.72(29.61,49.99) | | -0.19(-0.35,-0.02) | | 0.012(-0.006,0.031) | |  |
| Yemen | 1450.42(1225.01,1755.27) | | 31.55(26.65,38.18) | 3737.20(3165.75,4538.30) | | 27.16(23.01,32.98) | | 157.66(139.04,178.51) | | -0.37(-0.43,-0.31) | | -0.142(-0.147,-0.138) | |  |
| Zambia | 932.54(782.30,1136.80) | | 30.73(25.78,37.47) | 2111.98(1763.11,2572.62) | | 26.10(21.79,31.79) | | 126.48(112.66,140.91) | | -0.68(-0.78,-0.58) | | -0.141(-0.147,-0.136) | |  |
| Zimbabwe | 747.78(603.82,951.57) | | 18.86(15.23,24.00) | 1504.82(1249.07,1864.84) | | 23.74(19.71,29.42) | | 101.24(84.52,119.39) | | 1.03(0.81,1.25) | | 0.162(0.153,0.171) | |  |

Note: EAPC, estimated annual percentage change; AAPC, average annual percentage change; CI, confidence interval; GBD, Global Burden of Disease; SDI, socio-demographic

| **Table S3. Age-standardized morality rate due to stroke and their temporal change among youths and young adults (15-39 years) in 204 countries or territories from 1990 to 2021** | | | | | | | | |
| --- | --- | --- | --- | --- | --- | --- | --- | --- |
|  | **Rate per 100 000 (95% UI)** | |  |  |  |  |  |  |
|  | **1990** |  | **2021** |  | **1990-2021** |  |  |  |
| **Countries or Territories** | **Mortality cases** | **Mortality rate** | **Mortality cases** | **Mortality rate** | **Cases change(%)** | **EAPC** | **AAPC** |  |
| Afghanistan | 388.69(264.07,516.59) | 12.34(8.38,16.40) | 1135.45(818.75,1530.50) | 9.29(6.70,12.52) | 192.12(109.99,323.88) | -0.77(-1.28,-0.26) | -0.100(-0.117,-0.083) |  |
| Albania | 61.29(50.45,72.38) | 4.31(3.55,5.10) | 21.41(15.62,27.45) | 2.26(1.65,2.89) | -65.07(-75.83,-51.88) | -2.53(-2.95,-2.10) | -0.070(-0.082,-0.058) |  |
| Algeria | 651.21(488.47,835.35) | 6.44(4.83,8.27) | 629.71(452.50,850.65) | 3.70(2.66,5.00) | -3.30(-28.58,31.57) | -2.09(-2.32,-1.87) | -0.084(-0.088,-0.081) |  |
| American Samoa | 1.99(1.61,2.47) | 9.81(7.94,12.22) | 1.61(1.21,2.08) | 9.23(6.90,11.88) | -18.71(-43.57,15.67) | -0.44(-0.58,-0.29) | -0.024(-0.031,-0.017) |  |
| Andorra | 0.40(0.28,0.54) | 1.59(1.14,2.17) | 0.17(0.11,0.24) | 0.68(0.44,0.95) | -56.10(-73.46,-31.79) | -2.57(-2.81,-2.33) | -0.031(-0.032,-0.029) |  |
| Angola | 224.66(165.97,292.15) | 5.74(4.24,7.47) | 442.33(304.82,586.80) | 3.64(2.51,4.82) | 96.89(26.02,193.96) | -1.40(-1.58,-1.22) | -0.073(-0.087,-0.058) |  |
| Antigua and Barbuda | 1.40(1.27,1.53) | 5.42(4.94,5.93) | 0.59(0.52,0.67) | 1.72(1.52,1.96) | -57.60(-64.07,-49.40) | -3.04(-3.54,-2.53) | -0.095(-0.112,-0.078) |  |
| Argentina | 992.46(925.07,1055.38) | 8.12(7.57,8.64) | 406.44(373.51,441.99) | 2.32(2.13,2.52) | -59.05(-63.39,-54.67) | -3.78(-4.17,-3.38) | -0.198(-0.206,-0.190) |  |
| Armenia | 42.08(36.01,47.84) | 2.93(2.51,3.33) | 12.51(10.52,14.49) | 1.16(0.98,1.35) | -70.28(-76.35,-62.50) | -3.89(-4.38,-3.41) | -0.055(-0.070,-0.041) |  |
| Australia | 112.44(103.24,122.52) | 1.66(1.52,1.81) | 59.69(53.70,66.89) | 0.69(0.62,0.77) | -46.92(-53.49,-38.73) | -3.32(-3.66,-2.99) | -0.030(-0.033,-0.028) |  |
| Austria | 73.33(68.81,78.37) | 2.44(2.29,2.61) | 17.27(15.83,18.87) | 0.61(0.56,0.67) | -76.45(-78.89,-73.88) | -4.99(-5.30,-4.68) | -0.058(-0.061,-0.056) |  |
| Azerbaijan | 151.97(111.96,201.83) | 4.78(3.52,6.35) | 85.99(54.76,121.96) | 2.03(1.29,2.88) | -43.42(-65.68,-8.70) | -4.27(-4.75,-3.78) | -0.087(-0.101,-0.073) |  |
| Bahamas | 6.68(5.99,7.34) | 5.66(5.08,6.22) | 5.47(4.28,6.79) | 3.54(2.77,4.39) | -18.18(-36.58,6.35) | -2.26(-2.50,-2.02) | -0.081(-0.097,-0.065) |  |
| Bahrain | 10.87(9.30,12.51) | 4.24(3.63,4.88) | 18.86(15.22,22.59) | 2.68(2.16,3.21) | 73.51(34.84,126.73) | -1.94(-2.33,-1.56) | -0.047(-0.053,-0.042) |  |
| Bangladesh | 4801.40(3840.45,5796.11) | 11.38(9.10,13.73) | 5211.04(3926.46,6839.38) | 7.57(5.71,9.94) | 8.53(-19.84,50.06) | -1.33(-1.61,-1.05) | -0.102(-0.112,-0.092) |  |
| Barbados | 5.04(4.62,5.49) | 4.61(4.24,5.03) | 2.47(1.93,3.20) | 2.50(1.96,3.24) | -50.94(-62.30,-35.20) | -2.47(-2.76,-2.18) | -0.075(-0.084,-0.066) |  |
| Belarus | 172.57(151.34,197.08) | 4.37(3.84,5.00) | 110.05(88.04,135.12) | 3.75(3.00,4.61) | -36.23(-51.23,-16.48) | -1.63(-2.21,-1.04) | -0.023(-0.039,-0.008) |  |
| Belgium | 121.89(113.07,131.19) | 3.28(3.04,3.53) | 24.43(22.22,26.90) | 0.70(0.63,0.77) | -79.96(-82.26,-77.24) | -4.92(-5.20,-4.65) | -0.085(-0.088,-0.081) |  |
| Belize | 2.98(2.66,3.30) | 4.07(3.64,4.51) | 4.67(4.02,5.30) | 2.47(2.13,2.81) | 56.90(31.64,85.70) | -1.85(-2.28,-1.43) | -0.052(-0.066,-0.037) |  |
| Benin | 76.04(60.37,97.57) | 4.47(3.55,5.73) | 186.68(137.81,238.39) | 3.56(2.63,4.55) | 145.50(71.68,245.98) | -0.80(-1.01,-0.60) | -0.029(-0.031,-0.027) |  |
| Bermuda | 0.63(0.56,0.70) | 2.46(2.19,2.73) | 0.15(0.12,0.19) | 0.88(0.70,1.09) | -75.69(-81.44,-69.28) | -3.63(-3.88,-3.38) | -0.053(-0.057,-0.049) |  |
| Bhutan | 8.54(5.45,12.31) | 3.17(2.02,4.57) | 7.41(4.72,10.70) | 2.14(1.36,3.09) | -13.19(-46.79,44.73) | -1.78(-1.94,-1.62) | -0.033(-0.035,-0.030) |  |
| Bolivia (Plurinational State of) | 288.30(224.77,378.63) | 11.69(9.11,15.35) | 231.56(164.70,318.81) | 4.71(3.35,6.49) | -19.68(-46.94,17.64) | -3.27(-3.55,-3.00) | -0.227(-0.230,-0.225) |  |
| Bosnia and Herzegovina | 80.10(63.08,100.00) | 4.22(3.32,5.27) | 19.29(13.19,25.71) | 1.92(1.31,2.55) | -75.92(-84.64,-65.91) | -2.98(-3.33,-2.63) | -0.076(-0.091,-0.062) |  |
| Botswana | 26.01(14.71,39.16) | 5.05(2.86,7.61) | 28.05(15.12,42.43) | 2.63(1.42,3.98) | 7.84(-32.38,65.83) | -2.27(-2.58,-1.97) | -0.093(-0.101,-0.085) |  |
| Brazil | 5499.96(5348.72,5652.22) | 8.77(8.52,9.01) | 3108.28(2989.71,3232.80) | 3.65(3.51,3.79) | -43.49(-46.07,-40.66) | -3.13(-3.38,-2.87) | -0.167(-0.172,-0.161) |  |
| Brunei Darussalam | 8.95(7.27,10.97) | 7.26(5.89,8.90) | 7.31(5.87,9.01) | 3.58(2.88,4.42) | -18.25(-39.49,9.85) | -2.83(-3.33,-2.32) | -0.117(-0.123,-0.112) |  |
| Bulgaria | 274.87(250.03,299.77) | 9.23(8.40,10.07) | 110.59(92.56,129.59) | 5.82(4.87,6.82) | -59.77(-67.19,-51.22) | -2.08(-2.31,-1.84) | -0.108(-0.132,-0.084) |  |
| Burkina Faso | 123.05(94.13,158.31) | 3.86(2.95,4.97) | 292.39(211.15,373.33) | 3.38(2.44,4.31) | 137.63(65.62,227.98) | -0.31(-0.50,-0.11) | -0.013(-0.018,-0.008) |  |
| Burundi | 269.62(195.99,353.49) | 13.01(9.45,17.05) | 307.79(228.50,397.21) | 5.84(4.33,7.53) | 14.16(-22.09,70.73) | -3.15(-3.62,-2.68) | -0.236(-0.240,-0.231) |  |
| Cabo Verde | 8.94(7.16,10.94) | 6.83(5.47,8.37) | 8.25(5.61,11.26) | 3.29(2.24,4.49) | -7.70(-34.47,27.72) | -2.50(-2.79,-2.22) | -0.123(-0.131,-0.115) |  |
| Cambodia | 336.70(261.57,414.57) | 8.74(6.79,10.77) | 384.66(269.04,559.89) | 5.31(3.71,7.73) | 14.24(-26.03,78.87) | -2.17(-2.40,-1.94) | -0.110(-0.114,-0.107) |  |
| Cameroon | 245.13(185.13,316.38) | 6.45(4.87,8.32) | 739.28(508.29,1001.90) | 5.73(3.94,7.77) | 201.58(99.47,346.31) | -0.34(-0.74,0.06) | -0.024(-0.027,-0.021) |  |
| Canada | 199.53(183.67,216.91) | 1.79(1.65,1.95) | 122.42(110.13,135.10) | 1.03(0.93,1.14) | -38.65(-46.59,-30.23) | -2.21(-2.76,-1.66) | -0.024(-0.028,-0.021) |  |
| Central African Republic | 73.76(53.94,98.86) | 7.08(5.18,9.49) | 132.71(86.35,193.54) | 6.08(3.96,8.86) | 79.91(24.21,157.75) | -0.65(-0.76,-0.54) | -0.034(-0.041,-0.027) |  |
| Chad | 119.07(90.82,154.03) | 5.67(4.33,7.34) | 343.84(241.48,468.77) | 5.46(3.84,7.45) | 188.78(99.32,320.53) | -0.07(-0.32,0.17) | -0.007(-0.015,0.001) |  |
| Chile | 215.70(202.61,230.71) | 3.77(3.54,4.03) | 124.82(115.59,136.06) | 1.76(1.63,1.92) | -42.13(-48.09,-36.07) | -2.41(-2.56,-2.25) | -0.064(-0.069,-0.059) |  |
| China | 39207.30(34017.57,44969.43) | 7.15(6.21,8.20) | 23099.87(19113.48,27336.47) | 5.01(4.14,5.92) | -41.08(-53.34,-25.53) | -1.48(-1.72,-1.24) | -0.070(-0.076,-0.064) |  |
| Colombia | 663.53(622.53,704.34) | 4.72(4.43,5.01) | 420.46(345.82,498.80) | 2.09(1.72,2.48) | -36.63(-47.80,-24.42) | -2.58(-2.99,-2.17) | -0.084(-0.091,-0.078) |  |
| Comoros | 13.86(5.92,19.46) | 8.03(3.43,11.27) | 13.70(10.00,17.97) | 4.42(3.23,5.80) | -1.20(-35.97,122.60) | -2.70(-3.48,-1.92) | -0.123(-0.150,-0.095) |  |
| Congo | 62.26(43.11,85.08) | 6.57(4.55,8.98) | 101.74(68.65,151.02) | 4.59(3.10,6.82) | 63.40(4.96,160.05) | -1.41(-1.68,-1.15) | -0.063(-0.075,-0.050) |  |
| Cook Islands | 0.73(0.54,1.00) | 9.43(6.98,12.90) | 0.30(0.21,0.42) | 5.04(3.57,7.08) | -59.21(-75.21,-32.60) | -1.64(-2.01,-1.28) | -0.142(-0.150,-0.133) |  |
| Costa Rica | 29.22(26.91,31.49) | 2.27(2.09,2.45) | 30.43(26.63,33.86) | 1.60(1.40,1.78) | 4.12(-10.23,21.24) | -1.69(-2.13,-1.24) | -0.025(-0.030,-0.020) |  |
| Croatia | 77.15(69.49,85.02) | 4.25(3.83,4.69) | 10.96(9.08,13.22) | 0.88(0.73,1.06) | -85.79(-88.72,-82.52) | -5.19(-5.43,-4.95) | -0.109(-0.113,-0.105) |  |
| Cuba | 195.21(181.86,209.86) | 4.00(3.73,4.30) | 71.36(61.22,82.10) | 1.99(1.71,2.29) | -63.44(-69.05,-57.31) | -2.49(-2.75,-2.24) | -0.069(-0.077,-0.061) |  |
| Cyprus | 6.37(5.15,7.66) | 2.07(1.67,2.49) | 3.61(2.83,4.53) | 0.72(0.56,0.90) | -43.27(-58.06,-23.74) | -4.44(-5.04,-3.84) | -0.041(-0.044,-0.038) |  |
| Czechia | 127.19(113.16,142.36) | 3.43(3.05,3.84) | 30.02(24.98,36.31) | 1.02(0.85,1.23) | -76.40(-81.20,-70.97) | -3.37(-3.63,-3.10) | -0.078(-0.081,-0.075) |  |
| Côte d'Ivoire | 310.75(237.73,405.91) | 6.57(5.03,8.58) | 648.57(450.23,881.52) | 5.78(4.02,7.86) | 108.71(41.39,208.91) | -0.25(-0.45,-0.06) | -0.026(-0.030,-0.022) |  |
| Democratic People's Republic of Korea | 706.51(490.83,1008.38) | 8.47(5.88,12.09) | 1054.35(727.86,1571.14) | 10.48(7.24,15.62) | 49.23(-3.37,143.10) | 0.33(0.07,0.58) | 0.067(0.065,0.069) |  |
| Democratic Republic of the Congo | 641.04(454.39,866.28) | 4.47(3.17,6.05) | 1204.26(828.48,1668.75) | 3.34(2.30,4.62) | 87.86(36.36,174.07) | -1.04(-1.12,-0.95) | -0.037(-0.041,-0.034) |  |
| Denmark | 50.91(47.22,54.55) | 2.67(2.47,2.86) | 11.27(10.26,12.35) | 0.62(0.56,0.68) | -77.85(-80.25,-75.09) | -5.06(-5.45,-4.66) | -0.068(-0.074,-0.063) |  |
| Djibouti | 9.88(6.79,13.49) | 5.64(3.87,7.69) | 22.63(14.33,33.93) | 4.18(2.65,6.27) | 128.99(39.30,262.19) | -1.14(-1.38,-0.90) | -0.040(-0.054,-0.026) |  |
| Dominica | 0.83(0.70,0.98) | 2.83(2.41,3.35) | 0.71(0.55,0.92) | 2.75(2.12,3.56) | -14.03(-37.43,15.87) | -0.36(-0.59,-0.13) | -0.005(-0.008,-0.002) |  |
| Dominican Republic | 188.14(158.32,220.40) | 6.12(5.15,7.17) | 239.77(187.30,310.98) | 5.27(4.12,6.84) | 27.44(-5.90,72.42) | 0.17(-0.18,0.52) | -0.035(-0.055,-0.016) |  |
| Ecuador | 319.28(298.33,340.22) | 7.74(7.23,8.25) | 241.06(192.83,300.57) | 3.30(2.64,4.11) | -24.50(-40.36,-6.35) | -2.87(-3.29,-2.46) | -0.177(-0.201,-0.153) |  |
| Egypt | 2278.33(1829.77,2720.98) | 10.39(8.35,12.41) | 2201.76(1627.25,2842.80) | 5.22(3.85,6.73) | -3.36(-27.75,29.41) | -1.88(-2.25,-1.50) | -0.168(-0.181,-0.154) |  |
| El Salvador | 132.31(115.72,150.05) | 6.33(5.54,7.18) | 71.39(54.93,88.79) | 2.75(2.12,3.43) | -46.04(-59.77,-30.74) | -2.60(-3.18,-2.02) | -0.132(-0.147,-0.116) |  |
| Equatorial Guinea | 10.28(7.34,14.45) | 6.81(4.86,9.57) | 18.42(10.48,29.78) | 2.65(1.51,4.28) | 79.12(5.97,206.59) | -3.68(-4.13,-3.23) | -0.130(-0.136,-0.123) |  |
| Eritrea | 140.20(104.28,182.19) | 10.82(8.05,14.06) | 192.62(126.16,273.45) | 6.88(4.51,9.77) | 37.39(-11.28,100.73) | -1.54(-1.68,-1.41) | -0.119(-0.132,-0.107) |  |
| Estonia | 18.65(16.00,21.33) | 3.28(2.82,3.76) | 3.01(2.50,3.54) | 0.76(0.63,0.89) | -83.84(-87.43,-79.67) | -6.28(-6.86,-5.69) | -0.079(-0.085,-0.072) |  |
| Eswatini | 11.87(7.77,15.94) | 3.94(2.58,5.29) | 26.40(15.81,39.01) | 5.19(3.10,7.66) | 122.47(27.47,284.85) | 1.42(0.60,2.25) | 0.043(0.037,0.048) |  |
| Ethiopia | 1450.90(1163.52,2035.22) | 7.94(6.37,11.14) | 1421.00(1105.07,1765.30) | 3.07(2.38,3.81) | -2.06(-41.00,35.87) | -3.60(-3.84,-3.36) | -0.159(-0.161,-0.157) |  |
| Fiji | 41.02(34.16,49.39) | 12.72(10.59,15.32) | 33.95(25.74,44.65) | 9.52(7.22,12.52) | -17.24(-40.71,14.31) | -1.03(-1.23,-0.82) | -0.101(-0.112,-0.091) |  |
| Finland | 76.24(69.79,83.12) | 4.20(3.84,4.58) | 17.41(15.68,19.29) | 1.05(0.94,1.16) | -77.16(-80.16,-73.96) | -4.44(-4.79,-4.09) | -0.099(-0.106,-0.092) |  |
| France | 491.62(456.81,528.85) | 2.23(2.08,2.40) | 156.46(142.80,172.06) | 0.79(0.72,0.87) | -68.17(-71.92,-64.57) | -3.52(-3.69,-3.35) | -0.045(-0.047,-0.043) |  |
| Gabon | 17.22(12.94,22.52) | 4.48(3.36,5.86) | 20.58(12.90,31.17) | 2.75(1.72,4.16) | 19.56(-23.14,91.69) | -1.81(-2.02,-1.61) | -0.052(-0.058,-0.047) |  |
| Gambia | 24.78(18.19,32.31) | 6.57(4.82,8.57) | 63.20(45.23,87.98) | 6.32(4.52,8.80) | 155.02(65.94,294.61) | -0.43(-0.73,-0.13) | -0.012(-0.030,0.005) |  |
| Georgia | 162.20(132.05,191.07) | 7.62(6.20,8.97) | 47.94(38.23,58.51) | 4.23(3.37,5.16) | -70.44(-77.72,-60.77) | -3.30(-4.49,-2.10) | -0.102(-0.166,-0.038) |  |
| Germany | 1100.11(1029.66,1178.22) | 3.70(3.47,3.97) | 214.66(197.91,233.58) | 0.85(0.78,0.92) | -80.49(-82.41,-78.23) | -4.72(-5.00,-4.43) | -0.092(-0.094,-0.089) |  |
| Ghana | 659.55(507.10,834.31) | 11.49(8.83,14.53) | 1078.56(791.39,1409.08) | 7.54(5.53,9.85) | 63.53(13.00,145.38) | -1.23(-1.39,-1.07) | -0.127(-0.131,-0.123) |  |
| Greece | 150.29(139.00,162.20) | 4.00(3.70,4.31) | 45.78(41.30,50.82) | 1.64(1.48,1.83) | -69.54(-73.26,-65.40) | -2.88(-3.07,-2.69) | -0.079(-0.082,-0.075) |  |
| Greenland | 2.35(1.89,2.91) | 8.87(7.14,10.98) | 0.64(0.43,0.82) | 3.13(2.12,4.01) | -72.81(-81.76,-62.86) | -4.38(-4.81,-3.95) | -0.184(-0.190,-0.178) |  |
| Grenada | 3.03(2.73,3.34) | 9.10(8.19,10.02) | 1.22(1.02,1.42) | 3.01(2.53,3.51) | -59.88(-67.48,-51.72) | -3.45(-3.80,-3.11) | -0.213(-0.224,-0.201) |  |
| Guam | 3.31(2.61,3.92) | 5.22(4.12,6.19) | 3.12(2.66,3.62) | 5.63(4.79,6.54) | -5.78(-24.97,26.45) | 0.29(-0.02,0.60) | 0.013(-0.004,0.030) |  |
| Guatemala | 185.12(173.17,198.01) | 6.27(5.86,6.70) | 234.78(200.21,271.14) | 3.45(2.94,3.98) | 26.82(5.40,48.30) | -2.79(-3.32,-2.26) | -0.074(-0.093,-0.055) |  |
| Guinea | 139.03(109.74,180.09) | 6.77(5.34,8.77) | 323.21(224.08,436.79) | 6.26(4.34,8.45) | 132.48(57.16,242.39) | 0.02(-0.09,0.13) | -0.016(-0.020,-0.012) |  |
| Guinea-Bissau | 50.68(37.80,67.21) | 13.67(10.19,18.12) | 86.90(63.71,115.03) | 10.30(7.55,13.63) | 71.48(18.72,149.72) | -0.85(-0.88,-0.81) | -0.108(-0.112,-0.104) |  |
| Guyana | 35.10(28.93,40.07) | 10.31(8.50,11.77) | 21.36(16.20,28.01) | 6.87(5.21,9.01) | -39.13(-55.81,-18.85) | -1.46(-1.81,-1.11) | -0.109(-0.155,-0.064) |  |
| Haiti | 405.80(309.60,513.74) | 16.67(12.72,21.10) | 608.60(423.06,848.98) | 11.09(7.71,15.47) | 49.98(3.20,110.33) | -1.01(-1.23,-0.79) | -0.157(-0.180,-0.135) |  |
| Honduras | 183.19(149.29,220.35) | 10.61(8.65,12.76) | 199.85(109.80,299.22) | 4.55(2.50,6.81) | 9.10(-43.18,67.14) | -3.33(-3.68,-2.98) | -0.199(-0.208,-0.189) |  |
| Hungary | 265.03(240.86,292.29) | 7.17(6.52,7.91) | 38.19(31.60,45.55) | 1.39(1.15,1.66) | -85.59(-88.47,-82.55) | -5.59(-5.98,-5.21) | -0.185(-0.193,-0.176) |  |
| Iceland | 2.35(2.15,2.57) | 2.26(2.07,2.47) | 0.81(0.71,0.91) | 0.67(0.59,0.76) | -65.70(-70.61,-60.06) | -3.58(-3.89,-3.28) | -0.049(-0.051,-0.048) |  |
| India | 13735.64(11827.72,16014.17) | 4.03(3.47,4.70) | 16039.07(14107.82,17917.03) | 2.63(2.31,2.94) | 16.77(-4.22,39.76) | -1.42(-1.70,-1.14) | -0.050(-0.055,-0.046) |  |
| Indonesia | 9848.33(8569.87,11346.47) | 12.62(10.98,14.54) | 10890.42(8973.26,14189.66) | 9.56(7.88,12.46) | 10.58(-11.90,51.47) | -0.79(-1.02,-0.55) | -0.100(-0.103,-0.098) |  |
| Iran (Islamic Republic of) | 882.87(778.17,1005.40) | 4.07(3.58,4.63) | 984.73(900.02,1070.53) | 2.84(2.59,3.08) | 11.54(-4.37,31.42) | -0.94(-1.14,-0.74) | -0.040(-0.042,-0.038) |  |
| Iraq | 738.76(598.49,907.90) | 10.28(8.33,12.64) | 943.25(711.56,1277.71) | 5.41(4.08,7.33) | 27.68(-6.79,85.42) | -2.16(-2.47,-1.85) | -0.165(-0.173,-0.157) |  |
| Ireland | 31.56(29.51,33.74) | 2.30(2.15,2.46) | 8.64(7.77,9.57) | 0.55(0.50,0.61) | -72.63(-76.02,-68.84) | -4.40(-4.76,-4.03) | -0.057(-0.061,-0.053) |  |
| Israel | 37.84(34.98,40.69) | 1.98(1.83,2.13) | 13.33(11.81,15.22) | 0.40(0.36,0.46) | -64.77(-69.71,-59.05) | -5.17(-5.37,-4.97) | -0.050(-0.053,-0.048) |  |
| Italy | 541.73(524.46,560.16) | 2.54(2.46,2.62) | 134.23(126.57,142.05) | 0.85(0.80,0.90) | -75.22(-76.91,-73.28) | -3.68(-3.87,-3.50) | -0.056(-0.060,-0.053) |  |
| Jamaica | 47.40(42.22,53.39) | 4.82(4.30,5.43) | 37.60(27.81,50.42) | 3.15(2.33,4.23) | -20.68(-43.27,9.58) | -2.18(-2.94,-1.42) | -0.051(-0.066,-0.036) |  |
| Japan | 1276.85(1244.67,1317.51) | 2.85(2.78,2.94) | 537.79(520.86,557.18) | 1.66(1.61,1.72) | -57.88(-59.69,-55.96) | -1.58(-1.83,-1.32) | -0.038(-0.041,-0.034) |  |
| Jordan | 92.74(76.55,113.25) | 6.03(4.98,7.37) | 121.34(97.57,147.95) | 2.26(1.82,2.76) | 30.83(-2.12,75.68) | -3.81(-4.15,-3.47) | -0.122(-0.127,-0.117) |  |
| Kazakhstan | 420.62(378.13,461.46) | 6.20(5.57,6.80) | 249.60(183.29,311.70) | 3.58(2.63,4.47) | -40.66(-56.44,-25.79) | -2.86(-3.71,-1.99) | -0.085(-0.102,-0.069) |  |
| Kenya | 291.99(223.33,353.85) | 3.33(2.55,4.04) | 647.62(476.66,841.56) | 2.99(2.20,3.89) | 121.80(75.08,188.62) | -0.02(-0.34,0.31) | -0.012(-0.013,-0.011) |  |
| Kiribati | 6.03(4.69,7.50) | 19.75(15.36,24.57) | 10.76(7.82,14.24) | 21.64(15.72,28.64) | 78.51(23.65,158.67) | 0.01(-0.15,0.16) | 0.061(0.056,0.065) |  |
| Kuwait | 23.48(21.07,25.58) | 2.78(2.49,3.03) | 37.10(30.61,44.78) | 1.75(1.44,2.11) | 58.01(29.76,90.80) | -2.08(-3.00,-1.16) | -0.033(-0.045,-0.021) |  |
| Kyrgyzstan | 140.50(120.59,161.04) | 7.79(6.69,8.93) | 112.18(90.82,138.44) | 4.12(3.34,5.09) | -20.15(-38.71,3.94) | -3.99(-4.62,-3.35) | -0.115(-0.132,-0.098) |  |
| Lao People's Democratic Republic | 240.66(182.68,310.86) | 15.58(11.82,20.12) | 316.02(223.86,438.32) | 9.85(6.98,13.66) | 31.31(-15.61,97.58) | -1.78(-1.89,-1.66) | -0.187(-0.190,-0.183) |  |
| Latvia | 37.77(34.39,41.57) | 3.96(3.60,4.36) | 9.39(7.58,11.70) | 1.74(1.41,2.17) | -75.15(-80.24,-69.43) | -4.09(-4.64,-3.54) | -0.065(-0.077,-0.053) |  |
| Lebanon | 89.36(65.19,119.84) | 7.75(5.65,10.40) | 56.76(46.02,69.59) | 2.45(1.98,3.00) | -36.48(-56.67,-5.58) | -3.41(-4.00,-2.81) | -0.166(-0.171,-0.161) |  |
| Lesotho | 14.78(8.33,20.97) | 2.74(1.54,3.89) | 50.55(34.92,69.78) | 6.08(4.20,8.39) | 242.04(112.73,570.94) | 3.91(3.27,4.56) | 0.110(0.104,0.116) |  |
| Liberia | 61.63(46.67,82.06) | 6.68(5.06,8.89) | 138.00(100.15,194.13) | 6.15(4.46,8.65) | 123.91(50.91,242.18) | -0.13(-0.41,0.14) | -0.021(-0.027,-0.015) |  |
| Libya | 94.12(71.56,124.25) | 5.60(4.26,7.40) | 183.43(126.67,256.26) | 6.11(4.22,8.54) | 94.90(39.89,173.58) | 0.76(0.43,1.09) | 0.028(0.020,0.035) |  |
| Lithuania | 42.46(37.69,48.20) | 3.05(2.71,3.46) | 9.85(8.29,11.67) | 1.22(1.03,1.45) | -76.80(-80.99,-71.50) | -3.04(-3.69,-2.38) | -0.061(-0.071,-0.050) |  |
| Luxembourg | 5.73(5.32,6.15) | 3.88(3.60,4.17) | 1.08(0.96,1.22) | 0.49(0.44,0.55) | -81.17(-83.57,-78.09) | -7.39(-7.73,-7.06) | -0.112(-0.116,-0.109) |  |
| Madagascar | 778.49(645.92,919.01) | 17.19(14.26,20.29) | 1451.43(1007.92,1918.68) | 12.39(8.61,16.38) | 86.44(25.27,158.16) | -1.05(-1.15,-0.95) | -0.156(-0.166,-0.147) |  |
| Malawi | 229.72(180.51,290.63) | 6.15(4.83,7.78) | 466.48(351.56,597.42) | 5.70(4.30,7.30) | 103.06(47.50,186.10) | -0.40(-0.65,-0.16) | -0.012(-0.018,-0.007) |  |
| Malaysia | 486.09(420.32,554.17) | 6.55(5.66,7.47) | 592.41(497.93,691.52) | 4.26(3.58,4.97) | 21.87(-3.34,53.03) | -1.48(-1.89,-1.07) | -0.080(-0.094,-0.066) |  |
| Maldives | 10.05(8.33,13.19) | 12.37(10.25,16.23) | 11.75(9.02,14.72) | 4.52(3.47,5.66) | 16.94(-18.71,57.66) | -3.68(-4.18,-3.19) | -0.254(-0.275,-0.234) |  |
| Mali | 240.80(183.31,307.92) | 8.07(6.14,10.31) | 478.05(349.79,622.51) | 5.37(3.93,6.99) | 98.53(41.17,177.58) | -1.23(-1.37,-1.09) | -0.087(-0.091,-0.083) |  |
| Malta | 3.56(3.25,3.88) | 2.58(2.36,2.81) | 1.11(0.97,1.26) | 0.83(0.73,0.94) | -68.76(-73.21,-63.39) | -3.25(-3.49,-3.02) | -0.055(-0.060,-0.051) |  |
| Marshall Islands | 3.35(2.66,4.16) | 19.51(15.49,24.27) | 5.25(3.76,7.11) | 22.11(15.85,29.99) | 56.71(11.95,116.12) | 0.61(0.48,0.75) | 0.074(0.064,0.085) |  |
| Mauritania | 57.62(43.87,75.04) | 7.49(5.71,9.76) | 59.73(38.33,92.17) | 3.50(2.25,5.40) | 3.65(-30.00,48.78) | -2.54(-2.64,-2.45) | -0.131(-0.135,-0.126) |  |
| Mauritius | 42.51(38.97,46.63) | 8.55(7.84,9.38) | 32.27(29.30,34.95) | 7.09(6.44,7.68) | -24.09(-33.36,-13.74) | 0.73(0.28,1.18) | 0.003(-0.020,0.027) |  |
| Mexico | 1150.94(1122.88,1181.44) | 3.23(3.15,3.31) | 1295.77(1170.42,1426.64) | 2.52(2.27,2.77) | 12.58(1.10,24.10) | -0.71(-1.04,-0.37) | -0.022(-0.024,-0.019) |  |
| Micronesia (Federated States of) | 9.25(6.50,11.71) | 23.09(16.22,29.24) | 8.35(6.15,10.98) | 19.67(14.47,25.85) | -9.71(-35.94,30.54) | -0.45(-0.50,-0.40) | -0.108(-0.111,-0.106) |  |
| Monaco | 0.34(0.26,0.43) | 3.73(2.88,4.74) | 0.17(0.11,0.26) | 1.82(1.13,2.80) | -50.21(-70.58,-21.05) | -2.46(-2.67,-2.25) | -0.062(-0.063,-0.060) |  |
| Mongolia | 50.98(39.09,66.23) | 5.77(4.42,7.49) | 64.20(45.38,85.05) | 5.09(3.60,6.74) | 25.92(-14.98,79.69) | -0.92(-1.46,-0.38) | -0.047(-0.066,-0.027) |  |
| Montenegro | 17.62(14.24,21.30) | 7.02(5.67,8.49) | 7.19(5.51,9.38) | 3.49(2.68,4.56) | -59.20(-70.08,-44.48) | -2.54(-3.07,-2.01) | -0.107(-0.125,-0.089) |  |
| Morocco | 835.74(589.98,1161.53) | 8.04(5.67,11.17) | 617.49(402.22,999.49) | 4.21(2.74,6.81) | -26.11(-50.50,22.67) | -2.25(-2.48,-2.02) | -0.124(-0.128,-0.120) |  |
| Mozambique | 257.85(198.58,325.19) | 5.44(4.19,6.86) | 857.41(589.96,1162.09) | 7.13(4.91,9.67) | 232.52(120.08,376.34) | 1.70(1.38,2.01) | 0.056(0.052,0.060) |  |
| Myanmar | 3077.82(2361.86,4002.37) | 17.93(13.76,23.31) | 2360.55(1766.59,3160.30) | 10.50(7.86,14.06) | -23.30(-45.09,12.31) | -1.91(-2.19,-1.64) | -0.244(-0.251,-0.237) |  |
| Namibia | 21.36(13.27,28.76) | 3.82(2.37,5.14) | 32.44(18.23,51.96) | 3.10(1.74,4.97) | 51.82(-5.38,140.74) | -1.07(-1.58,-0.57) | -0.023(-0.028,-0.018) |  |
| Nauru | 1.21(0.86,1.74) | 29.84(21.39,43.15) | 1.38(0.99,1.98) | 29.56(21.22,42.56) | 14.02(-17.91,61.76) | -0.16(-0.63,0.32) | 0.002(-0.018,0.022) |  |
| Nepal | 326.44(233.23,454.92) | 4.47(3.19,6.23) | 338.09(239.07,480.21) | 2.52(1.78,3.58) | 3.57(-32.62,57.28) | -1.88(-1.99,-1.77) | -0.065(-0.067,-0.063) |  |
| Netherlands | 138.10(127.93,147.48) | 2.29(2.12,2.45) | 29.80(27.37,32.39) | 0.56(0.52,0.61) | -78.42(-80.65,-75.69) | -5.15(-5.59,-4.71) | -0.058(-0.061,-0.055) |  |
| New Zealand | 30.62(28.71,32.52) | 2.22(2.08,2.35) | 13.72(12.62,14.92) | 0.76(0.70,0.83) | -55.21(-60.13,-50.14) | -3.98(-4.23,-3.73) | -0.047(-0.052,-0.043) |  |
| Nicaragua | 54.59(47.86,62.08) | 3.70(3.24,4.21) | 58.82(47.17,72.18) | 2.07(1.66,2.54) | 7.76(-16.79,39.85) | -1.83(-2.08,-1.57) | -0.056(-0.063,-0.049) |  |
| Niger | 144.37(104.78,200.22) | 5.19(3.76,7.19) | 325.27(222.34,440.47) | 3.65(2.49,4.94) | 125.31(57.93,218.99) | -1.19(-1.33,-1.05) | -0.049(-0.051,-0.046) |  |
| Nigeria | 1257.34(974.19,1601.82) | 3.68(2.85,4.69) | 1962.46(1396.34,2753.85) | 2.18(1.55,3.06) | 56.08(7.33,132.15) | -1.97(-2.19,-1.76) | -0.048(-0.049,-0.047) |  |
| Niue | 0.10(0.07,0.15) | 12.48(8.92,18.18) | 0.07(0.05,0.09) | 12.05(9.39,15.71) | -31.90(-54.41,0.92) | -1.05(-1.32,-0.78) | -0.004(-0.025,0.016) |  |
| North Macedonia | 60.55(51.86,69.98) | 7.63(6.53,8.81) | 24.50(18.08,31.73) | 3.20(2.36,4.15) | -59.54(-70.00,-46.22) | -2.88(-3.17,-2.58) | -0.145(-0.158,-0.132) |  |
| Northern Mariana Islands | 2.58(1.68,3.58) | 11.03(7.17,15.29) | 1.04(0.82,1.34) | 6.34(4.95,8.13) | -59.57(-72.61,-36.28) | -2.18(-2.39,-1.97) | -0.170(-0.188,-0.152) |  |
| Norway | 31.74(30.57,32.95) | 1.98(1.91,2.06) | 7.05(6.70,7.42) | 0.40(0.38,0.42) | -77.79(-79.20,-76.36) | -5.37(-5.71,-5.02) | -0.050(-0.052,-0.047) |  |
| Oman | 45.25(33.12,59.82) | 5.45(3.99,7.21) | 62.41(47.05,80.21) | 2.70(2.03,3.47) | 37.94(-8.86,102.35) | -1.55(-1.85,-1.25) | -0.089(-0.096,-0.082) |  |
| Pakistan | 1521.89(1146.92,1873.71) | 3.73(2.81,4.59) | 4685.27(3483.49,6110.98) | 4.74(3.52,6.18) | 207.86(123.36,347.59) | 0.39(0.05,0.74) | 0.038(0.034,0.041) |  |
| Palau | 1.14(0.83,1.51) | 16.39(11.86,21.60) | 1.16(0.93,1.45) | 19.75(15.83,24.68) | 1.79(-29.29,50.24) | 0.77(0.64,0.89) | 0.104(0.078,0.131) |  |
| Palestine | 45.78(34.63,59.82) | 5.96(4.51,7.79) | 65.31(53.18,76.75) | 2.99(2.44,3.51) | 42.66(4.72,102.59) | -2.25(-2.56,-1.93) | -0.096(-0.101,-0.092) |  |
| Panama | 36.56(33.78,39.26) | 3.61(3.34,3.88) | 40.31(33.35,48.65) | 2.44(2.02,2.95) | 10.25(-11.52,35.58) | -1.33(-1.63,-1.03) | -0.037(-0.042,-0.033) |  |
| Papua New Guinea | 155.86(97.43,238.97) | 9.42(5.89,14.44) | 355.52(228.25,501.59) | 8.31(5.33,11.72) | 128.11(41.17,283.70) | -0.58(-0.74,-0.43) | -0.034(-0.046,-0.022) |  |
| Paraguay | 85.37(72.77,100.21) | 5.45(4.64,6.39) | 85.42(64.65,110.82) | 2.79(2.11,3.62) | 0.05(-27.13,34.09) | -2.16(-2.32,-2.00) | -0.076(-0.083,-0.068) |  |
| Peru | 655.93(552.24,771.22) | 7.39(6.23,8.69) | 623.36(477.12,791.76) | 4.19(3.21,5.33) | -4.96(-31.10,26.71) | -1.80(-2.14,-1.46) | -0.082(-0.102,-0.063) |  |
| Philippines | 1797.82(1641.74,1955.92) | 6.94(6.33,7.55) | 3720.07(3173.97,4288.73) | 7.87(6.72,9.08) | 106.92(73.82,146.54) | 1.21(0.83,1.59) | 0.031(0.024,0.038) |  |
| Poland | 920.85(885.99,957.55) | 6.37(6.13,6.63) | 261.58(240.05,283.73) | 2.16(1.98,2.35) | -71.59(-74.20,-68.81) | -3.45(-3.79,-3.11) | -0.135(-0.140,-0.131) |  |
| Portugal | 233.60(218.63,248.23) | 6.17(5.77,6.56) | 40.68(36.94,44.38) | 1.38(1.25,1.50) | -82.59(-84.60,-80.68) | -5.36(-5.76,-4.96) | -0.153(-0.163,-0.142) |  |
| Puerto Rico | 36.35(33.38,39.70) | 2.57(2.36,2.81) | 11.78(9.85,13.99) | 1.14(0.95,1.35) | -67.60(-73.43,-61.33) | -3.26(-3.59,-2.92) | -0.044(-0.049,-0.039) |  |
| Qatar | 11.24(9.02,13.83) | 4.75(3.81,5.85) | 27.67(20.75,36.87) | 1.67(1.26,2.23) | 146.25(72.13,248.93) | -3.86(-4.29,-3.43) | -0.093(-0.104,-0.082) |  |
| Republic of Korea | 1499.17(1274.67,1752.22) | 7.12(6.06,8.32) | 259.04(221.08,324.03) | 1.62(1.38,2.02) | -82.72(-86.20,-76.92) | -5.35(-5.68,-5.03) | -0.177(-0.182,-0.172) |  |
| Republic of Moldova | 87.94(77.75,99.29) | 5.05(4.46,5.70) | 31.22(26.27,37.05) | 2.52(2.12,2.99) | -64.50(-71.25,-55.99) | -2.92(-3.29,-2.56) | -0.077(-0.092,-0.061) |  |
| Romania | 477.14(433.82,524.77) | 5.49(4.99,6.04) | 168.34(141.95,194.80) | 3.12(2.63,3.61) | -64.72(-71.27,-57.57) | -1.90(-2.07,-1.74) | -0.074(-0.082,-0.067) |  |
| Russian Federation | 3078.34(2998.86,3141.78) | 5.29(5.16,5.40) | 2706.55(2479.28,2892.52) | 5.82(5.33,6.22) | -12.08(-19.76,-5.23) | -0.37(-0.84,0.10) | 0.027(0.011,0.043) |  |
| Rwanda | 431.77(330.56,560.06) | 15.75(12.06,20.44) | 232.21(154.33,322.25) | 4.09(2.72,5.68) | -46.22(-66.46,-17.83) | -5.68(-6.35,-5.01) | -0.384(-0.398,-0.370) |  |
| Saint Kitts and Nevis | 2.03(1.86,2.22) | 11.75(10.75,12.85) | 0.60(0.43,0.83) | 2.65(1.88,3.64) | -70.37(-79.63,-59.01) | -5.60(-6.28,-4.91) | -0.295(-0.303,-0.286) |  |
| Saint Lucia | 3.37(3.12,3.65) | 5.99(5.54,6.48) | 2.03(1.66,2.41) | 3.07(2.51,3.64) | -39.88(-51.15,-27.15) | -2.58(-2.90,-2.25) | -0.114(-0.131,-0.097) |  |
| Saint Vincent and the Grenadines | 2.87(2.61,3.14) | 6.24(5.69,6.85) | 1.65(1.41,1.91) | 3.99(3.41,4.62) | -42.43(-52.42,-31.94) | -2.24(-2.56,-1.92) | -0.067(-0.078,-0.055) |  |
| Samoa | 6.83(5.01,9.32) | 10.20(7.48,13.92) | 8.94(6.24,12.23) | 11.13(7.77,15.22) | 30.98(-14.00,92.86) | 0.30(0.14,0.47) | 0.029(0.024,0.035) |  |
| San Marino | 0.14(0.11,0.17) | 1.49(1.22,1.82) | 0.05(0.03,0.07) | 0.54(0.32,0.78) | -65.70(-80.19,-48.27) | -2.90(-3.17,-2.64) | -0.028(-0.030,-0.025) |  |
| Sao Tome and Principe | 2.41(1.53,3.29) | 5.62(3.56,7.66) | 5.06(3.25,7.68) | 5.57(3.57,8.45) | 110.00(13.67,294.14) | -0.39(-0.99,0.21) | 0.008(-0.024,0.039) |  |
| Saudi Arabia | 477.70(351.83,630.05) | 7.19(5.29,9.48) | 1231.05(867.02,1722.53) | 6.65(4.68,9.30) | 157.71(59.76,315.88) | 0.11(-0.12,0.35) | -0.016(-0.020,-0.012) |  |
| Senegal | 213.51(171.45,264.89) | 7.74(6.21,9.60) | 307.51(231.88,413.84) | 4.77(3.60,6.42) | 44.03(-1.74,105.01) | -1.33(-1.52,-1.14) | -0.102(-0.118,-0.086) |  |
| Serbia | 256.79(211.06,302.79) | 7.15(5.88,8.43) | 62.00(47.07,77.20) | 2.09(1.59,2.60) | -75.85(-83.03,-66.97) | -4.17(-4.34,-4.00) | -0.161(-0.169,-0.153) |  |
| Seychelles | 2.43(2.11,2.80) | 7.79(6.74,8.95) | 1.68(1.38,1.99) | 4.39(3.61,5.18) | -30.71(-44.94,-14.21) | -1.54(-1.82,-1.27) | -0.089(-0.118,-0.060) |  |
| Sierra Leone | 111.05(78.09,147.37) | 6.95(4.89,9.23) | 245.33(170.55,341.81) | 6.58(4.57,9.16) | 120.92(54.26,220.25) | 0.17(-0.02,0.37) | -0.011(-0.019,-0.004) |  |
| Singapore | 41.27(38.21,44.59) | 2.73(2.53,2.95) | 18.20(16.28,20.23) | 0.95(0.85,1.05) | -55.90(-61.61,-49.85) | -3.99(-4.37,-3.60) | -0.055(-0.062,-0.048) |  |
| Slovakia | 90.31(75.95,106.48) | 4.41(3.71,5.20) | 27.17(21.20,34.40) | 1.59(1.24,2.01) | -69.92(-77.42,-58.80) | -2.91(-3.17,-2.66) | -0.090(-0.094,-0.086) |  |
| Slovenia | 18.27(16.04,20.87) | 2.38(2.09,2.72) | 1.87(1.52,2.35) | 0.33(0.27,0.41) | -89.76(-92.03,-86.35) | -6.67(-6.96,-6.39) | -0.067(-0.070,-0.063) |  |
| Solomon Islands | 10.57(5.66,14.70) | 8.24(4.42,11.46) | 27.70(19.47,36.84) | 10.13(7.12,13.47) | 162.14(67.92,378.26) | 0.86(0.76,0.97) | 0.063(0.057,0.068) |  |
| Somalia | 276.50(196.88,375.31) | 9.54(6.79,12.94) | 487.68(333.95,694.99) | 5.88(4.03,8.38) | 76.38(17.83,168.90) | -1.42(-1.75,-1.09) | -0.114(-0.118,-0.110) |  |
| South Africa | 1361.18(1229.60,1502.93) | 8.65(7.81,9.55) | 1064.76(946.83,1209.99) | 4.39(3.90,4.99) | -21.78(-32.88,-9.42) | -2.84(-3.86,-1.81) | -0.165(-0.186,-0.144) |  |
| South Sudan | 135.21(95.34,186.38) | 5.86(4.13,8.07) | 171.17(114.30,248.72) | 4.76(3.18,6.91) | 26.60(-14.87,87.05) | -0.89(-1.41,-0.35) | -0.027(-0.035,-0.019) |  |
| Spain | 508.55(475.02,545.70) | 3.43(3.20,3.68) | 106.43(97.93,115.29) | 0.86(0.79,0.93) | -79.07(-81.65,-76.63) | -4.63(-4.79,-4.47) | -0.087(-0.091,-0.084) |  |
| Sri Lanka | 463.97(399.76,534.87) | 6.27(5.41,7.23) | 285.51(195.34,393.38) | 3.54(2.42,4.88) | -38.46(-60.38,-11.06) | -2.16(-2.50,-1.82) | -0.088(-0.104,-0.071) |  |
| Sudan | 894.01(603.00,1226.36) | 11.73(7.91,16.09) | 1164.30(695.47,1715.62) | 6.30(3.76,9.28) | 30.23(-23.51,113.94) | -1.99(-2.07,-1.91) | -0.176(-0.178,-0.174) |  |
| Suriname | 13.08(9.12,15.32) | 8.03(5.60,9.41) | 13.13(10.48,16.52) | 6.12(4.88,7.70) | 0.37(-23.70,43.04) | -1.32(-1.68,-0.96) | -0.062(-0.089,-0.034) |  |
| Sweden | 52.62(49.04,56.15) | 1.79(1.67,1.91) | 12.21(10.70,13.80) | 0.38(0.33,0.43) | -76.80(-79.82,-73.23) | -4.63(-4.81,-4.46) | -0.046(-0.049,-0.044) |  |
| Switzerland | 54.52(50.79,58.58) | 2.07(1.93,2.22) | 10.35(9.31,11.37) | 0.37(0.34,0.41) | -81.02(-83.66,-78.65) | -6.25(-6.54,-5.95) | -0.057(-0.059,-0.054) |  |
| Syrian Arab Republic | 715.72(565.02,893.23) | 14.92(11.78,18.62) | 322.78(243.93,432.10) | 6.35(4.80,8.50) | -54.90(-69.42,-33.30) | -2.76(-3.14,-2.38) | -0.283(-0.297,-0.269) |  |
| Taiwan (Province of China) | 426.35(398.87,456.05) | 4.62(4.32,4.94) | 176.20(158.00,193.72) | 2.34(2.09,2.57) | -58.67(-63.34,-53.76) | -1.82(-2.11,-1.53) | -0.075(-0.089,-0.061) |  |
| Tajikistan | 132.35(106.49,163.19) | 6.26(5.04,7.72) | 149.95(99.98,207.89) | 3.59(2.40,4.98) | 13.30(-31.01,68.34) | -3.20(-3.78,-2.62) | -0.088(-0.096,-0.079) |  |
| Thailand | 1363.40(1065.04,1677.01) | 5.26(4.11,6.47) | 1840.57(1392.72,2329.93) | 8.68(6.57,10.99) | 35.00(-3.39,91.55) | 0.68(-0.09,1.46) | 0.102(0.079,0.125) |  |
| Timor-Leste | 24.33(18.27,31.40) | 7.64(5.74,9.87) | 37.59(23.05,53.42) | 6.58(4.03,9.35) | 54.52(-6.76,124.54) | -0.52(-1.12,0.09) | -0.037(-0.050,-0.024) |  |
| Togo | 101.72(79.55,128.32) | 7.42(5.80,9.36) | 201.99(139.32,269.94) | 6.00(4.14,8.02) | 98.57(29.92,191.01) | -0.64(-0.81,-0.48) | -0.043(-0.046,-0.040) |  |
| Tokelau | 0.08(0.05,0.11) | 13.46(9.02,19.20) | 0.07(0.05,0.08) | 14.00(11.09,16.78) | -11.32(-37.06,32.66) | -0.63(-0.94,-0.32) | 0.048(0.024,0.072) |  |
| Tonga | 1.47(1.13,1.86) | 4.00(3.07,5.05) | 1.72(1.25,2.43) | 4.41(3.22,6.25) | 16.33(-21.82,82.02) | 0.52(0.40,0.65) | 0.012(0.009,0.015) |  |
| Trinidad and Tobago | 28.92(26.96,31.30) | 5.77(5.38,6.24) | 24.68(18.83,31.41) | 4.96(3.78,6.31) | -14.65(-35.76,12.19) | -1.06(-1.48,-0.64) | -0.023(-0.037,-0.009) |  |
| Tunisia | 140.08(106.06,183.18) | 4.07(3.08,5.33) | 131.24(86.36,182.88) | 3.02(1.98,4.20) | -6.31(-32.96,27.57) | -1.15(-1.22,-1.08) | -0.033(-0.036,-0.031) |  |
| Turkey | 1807.91(1451.01,2244.18) | 7.56(6.07,9.38) | 769.74(603.82,945.37) | 2.42(1.90,2.97) | -57.42(-68.67,-43.02) | -3.89(-4.19,-3.59) | -0.162(-0.166,-0.158) |  |
| Turkmenistan | 85.60(74.23,98.41) | 5.58(4.84,6.41) | 185.94(144.51,235.64) | 8.94(6.95,11.33) | 117.21(60.30,184.60) | 1.24(0.67,1.81) | 0.117(0.053,0.181) |  |
| Tuvalu | 0.81(0.62,1.05) | 22.52(17.11,29.00) | 0.81(0.62,1.02) | 16.37(12.56,20.47) | 0.08(-27.49,35.01) | -1.09(-1.19,-0.99) | -0.195(-0.201,-0.189) |  |
| Uganda | 324.10(230.54,436.37) | 5.05(3.59,6.80) | 626.91(443.35,869.39) | 3.65(2.58,5.05) | 93.43(21.55,198.06) | -2.19(-2.64,-1.73) | -0.049(-0.053,-0.045) |  |
| Ukraine | 700.40(605.20,802.85) | 3.69(3.19,4.23) | 724.72(513.29,972.88) | 5.26(3.72,7.06) | 3.47(-27.46,43.45) | 0.35(-0.03,0.73) | 0.034(0.016,0.053) |  |
| United Arab Emirates | 41.56(31.43,56.36) | 4.35(3.29,5.90) | 90.16(64.75,124.59) | 2.24(1.61,3.10) | 116.92(48.94,209.83) | -2.15(-2.38,-1.91) | -0.063(-0.073,-0.053) |  |
| United Kingdom | 511.97(504.29,519.85) | 2.45(2.41,2.49) | 195.28(189.83,200.61) | 0.90(0.87,0.92) | -61.86(-63.05,-60.68) | -3.38(-3.66,-3.10) | -0.049(-0.053,-0.045) |  |
| United Republic of Tanzania | 535.22(416.61,668.43) | 5.52(4.30,6.90) | 847.40(596.58,1163.44) | 3.63(2.56,4.99) | 58.33(4.47,132.34) | -1.68(-1.85,-1.50) | -0.061(-0.064,-0.058) |  |
| United States of America | 2387.24(2323.40,2453.74) | 2.34(2.27,2.40) | 1619.25(1512.76,1717.78) | 1.45(1.36,1.54) | -32.17(-37.32,-27.75) | -1.63(-1.86,-1.41) | -0.027(-0.030,-0.025) |  |
| United States Virgin Islands | 1.90(1.49,2.37) | 4.81(3.76,5.98) | 0.74(0.47,1.05) | 3.21(2.02,4.54) | -60.99(-75.04,-42.44) | -1.31(-1.65,-0.96) | -0.041(-0.055,-0.028) |  |
| Uruguay | 81.43(76.69,86.05) | 7.17(6.75,7.58) | 32.68(30.42,35.37) | 2.73(2.54,2.96) | -59.87(-63.69,-55.77) | -3.23(-3.58,-2.88) | -0.144(-0.153,-0.135) |  |
| Uzbekistan | 569.02(523.50,621.93) | 6.63(6.10,7.25) | 503.95(430.33,585.86) | 3.67(3.13,4.26) | -11.44(-26.65,5.19) | -2.16(-2.89,-1.42) | -0.102(-0.117,-0.087) |  |
| Vanuatu | 9.98(7.00,13.66) | 17.03(11.95,23.31) | 21.51(15.65,28.04) | 17.26(12.56,22.49) | 115.65(35.64,227.46) | -0.23(-0.33,-0.13) | 0.001(-0.012,0.015) |  |
| Venezuela (Bolivarian Republic of) | 398.30(369.03,429.99) | 4.97(4.61,5.37) | 407.11(297.51,530.78) | 4.35(3.18,5.67) | 2.21(-25.80,34.85) | -0.90(-1.43,-0.36) | -0.012(-0.020,-0.005) |  |
| Viet Nam | 2000.39(1485.47,2602.90) | 7.01(5.21,9.13) | 2195.71(1620.23,3096.34) | 5.72(4.22,8.07) | 9.76(-28.97,70.04) | -0.70(-0.96,-0.43) | -0.044(-0.046,-0.042) |  |
| Yemen | 356.87(190.08,518.14) | 7.76(4.14,11.27) | 754.71(468.61,1100.49) | 5.48(3.41,8.00) | 111.48(38.97,231.93) | -1.16(-1.35,-0.97) | -0.087(-0.098,-0.076) |  |
| Zambia | 216.56(170.31,273.61) | 7.14(5.61,9.02) | 429.42(271.12,627.97) | 5.31(3.35,7.76) | 98.29(20.35,186.28) | -1.31(-1.55,-1.07) | -0.063(-0.066,-0.059) |  |
| Zimbabwe | 78.68(62.50,98.18) | 1.98(1.58,2.48) | 374.34(266.73,511.09) | 5.91(4.21,8.06) | 375.75(217.27,622.51) | 4.72(3.56,5.90) | 0.126(0.119,0.133) |  |

Note: EAPC, estimated annual percentage change; AAPC, average annual percentage change; CI, confidence interval; GBD, Global Burden of Disease; SDI, socio-demographic

| **Table S4. Age-standardized DALY rate due to stroke and their temporal change among youths and young adults (15-39 years) in 204 countries or territories from 1990 to 2021** | | | | | | | | |
| --- | --- | --- | --- | --- | --- | --- | --- | --- |
|  | **Rate per 100 000 (95% UI)** | |  |  |  |  |  |  |
|  | **1990** |  | **2021** |  | **1990-2021** |  |  |  |
| **Countries and Territories** | **DALYs cases** | **DALYs rate** | **DALYs cases** | **DALYs rate** | **Cases change(%)** | **EAPC** | **AAPC** |  |
| Afghanistan | 25999.19(18315.01,33714.68) | 825.49(581.51,1070.46) | 76539.66(57461.40,100041.37) | 626.30(470.19,818.60) | 194.39(120.23,310.57) | -0.85(-1.28,-0.42) | -6.491(-7.552,-5.429) |  |
| Albania | 4715.78(4017.29,5439.17) | 331.99(282.81,382.91) | 1846.37(1488.15,2258.00) | 194.75(156.97,238.17) | -60.85(-69.41,-50.07) | -2.13(-2.47,-1.78) | -4.859(-5.283,-4.435) |  |
| Algeria | 46375.95(36090.43,57707.14) | 458.97(357.18,571.11) | 47215.13(36372.82,60723.14) | 277.30(213.62,356.63) | 1.81(-19.20,29.46) | -1.88(-2.07,-1.69) | -5.615(-5.805,-5.426) |  |
| American Samoa | 137.36(113.61,167.79) | 678.96(561.57,829.33) | 110.28(84.45,136.80) | 631.09(483.26,782.87) | -19.72(-41.51,8.33) | -0.46(-0.60,-0.32) | -1.940(-2.351,-1.530) |  |
| Andorra | 31.13(24.23,39.57) | 124.45(96.90,158.22) | 17.86(13.70,22.43) | 70.03(53.74,87.99) | -42.63(-58.71,-23.41) | -1.84(-2.00,-1.68) | -1.599(-1.709,-1.488) |  |
| Angola | 15454.93(11989.05,19641.02) | 395.00(306.42,501.99) | 31726.10(23558.42,40651.22) | 260.77(193.64,334.13) | 105.28(41.73,189.07) | -1.29(-1.44,-1.14) | -4.683(-5.406,-3.961) |  |
| Antigua and Barbuda | 90.30(82.52,98.24) | 350.16(319.99,380.96) | 45.42(40.31,51.17) | 132.05(117.19,148.76) | -49.70(-56.42,-41.69) | -2.68(-3.10,-2.26) | -6.137(-7.225,-5.049) |  |
| Argentina | 65465.36(61308.28,69551.16) | 535.93(501.90,569.38) | 31716.57(28978.08,34786.24) | 181.00(165.38,198.52) | -51.55(-55.88,-46.96) | -3.32(-3.67,-2.97) | -12.119(-12.636,-11.602) |  |
| Armenia | 3462.05(2986.05,3908.09) | 240.89(207.77,271.92) | 1424.30(1203.52,1678.95) | 132.49(111.95,156.17) | -58.86(-65.58,-52.01) | -2.67(-3.02,-2.32) | -3.443(-4.089,-2.798) |  |
| Australia | 8933.52(8053.78,9853.29) | 131.91(118.92,145.49) | 6204.93(5310.74,7122.73) | 71.57(61.26,82.16) | -30.54(-37.82,-22.28) | -2.33(-2.55,-2.11) | -1.781(-1.911,-1.651) |  |
| Austria | 5559.46(5110.58,6078.65) | 185.19(170.24,202.49) | 2041.26(1723.12,2383.10) | 72.32(61.05,84.43) | -63.28(-67.56,-58.84) | -3.47(-3.70,-3.24) | -3.612(-3.736,-3.488) |  |
| Azerbaijan | 11084.15(8584.82,13960.83) | 348.76(270.12,439.28) | 7627.67(5541.85,9862.87) | 180.07(130.83,232.84) | -31.18(-50.81,-5.57) | -3.46(-3.91,-3.01) | -5.309(-6.183,-4.435) |  |
| Bahamas | 429.84(384.80,466.91) | 364.22(326.06,395.64) | 360.47(290.65,435.24) | 233.07(187.92,281.41) | -16.14(-32.67,4.98) | -2.08(-2.29,-1.87) | -4.838(-5.779,-3.897) |  |
| Bahrain | 850.80(744.36,961.38) | 331.91(290.38,375.04) | 1522.36(1288.14,1772.61) | 216.38(183.09,251.95) | 78.93(48.80,118.06) | -1.74(-2.03,-1.45) | -3.548(-3.890,-3.206) |  |
| Bangladesh | 304657.13(244322.31,363072.46) | 721.77(578.83,860.16) | 335601.93(259417.68,430879.28) | 487.69(376.98,626.15) | 10.16(-18.02,48.17) | -1.26(-1.52,-0.99) | -6.246(-6.861,-5.631) |  |
| Barbados | 329.79(304.42,356.55) | 302.14(278.91,326.66) | 173.11(142.31,216.34) | 175.25(144.07,219.02) | -47.51(-57.87,-33.78) | -2.23(-2.49,-1.98) | -4.082(-4.731,-3.433) |  |
| Belarus | 12553.60(11012.21,14134.36) | 318.26(279.18,358.33) | 8116.76(6789.40,9732.39) | 276.67(231.43,331.75) | -35.34(-46.99,-20.96) | -1.34(-1.79,-0.88) | -1.491(-2.410,-0.572) |  |
| Belgium | 8472.64(7827.79,9169.06) | 227.72(210.39,246.44) | 2426.10(2095.19,2752.04) | 69.27(59.82,78.57) | -71.37(-74.56,-68.06) | -3.75(-3.98,-3.53) | -5.214(-5.410,-5.018) |  |
| Belize | 202.05(181.82,222.44) | 276.15(248.50,304.01) | 321.39(285.81,360.38) | 170.23(151.39,190.88) | 59.07(37.16,84.91) | -1.80(-2.17,-1.44) | -3.439(-4.248,-2.630) |  |
| Benin | 5613.65(4622.65,6939.28) | 329.67(271.47,407.52) | 14155.85(11117.28,17330.60) | 270.02(212.06,330.58) | 152.17(91.16,230.62) | -0.72(-0.89,-0.56) | -1.935(-2.079,-1.790) |  |
| Bermuda | 45.64(40.78,50.51) | 177.49(158.57,196.42) | 14.34(12.02,16.95) | 81.92(68.68,96.78) | -68.57(-73.60,-62.98) | -2.73(-2.93,-2.52) | -3.170(-3.456,-2.884) |  |
| Bhutan | 600.44(413.88,828.74) | 222.83(153.60,307.56) | 553.14(380.00,747.94) | 159.59(109.64,215.80) | -7.88(-38.76,39.63) | -1.52(-1.66,-1.39) | -2.026(-2.186,-1.867) |  |
| Bolivia (Plurinational State of) | 18569.65(14736.83,23970.43) | 753.01(597.59,972.02) | 15371.96(11225.05,20646.58) | 312.82(228.43,420.16) | -17.22(-43.66,18.20) | -3.14(-3.38,-2.90) | -14.361(-14.511,-14.211) |  |
| Bosnia and Herzegovina | 6242.39(5108.52,7582.63) | 328.70(269.00,399.27) | 2002.22(1573.54,2427.03) | 198.97(156.37,241.19) | -67.93(-75.14,-59.82) | -2.05(-2.32,-1.77) | -4.324(-5.226,-3.421) |  |
| Botswana | 1795.59(1102.36,2573.91) | 348.82(214.15,500.02) | 2125.59(1360.53,2993.73) | 199.52(127.70,281.00) | 18.38(-18.29,69.09) | -1.97(-2.22,-1.71) | -5.162(-5.810,-4.515) |  |
| Brazil | 337060.00(327287.99,347272.70) | 537.18(521.61,553.46) | 198673.70(189948.87,207382.02) | 233.05(222.81,243.26) | -41.06(-43.60,-38.47) | -2.96(-3.21,-2.72) | -9.888(-10.223,-9.553) |  |
| Brunei Darussalam | 600.98(503.64,722.83) | 487.46(408.50,586.29) | 506.24(413.33,600.68) | 248.11(202.57,294.40) | -15.76(-34.11,7.78) | -2.67(-3.11,-2.23) | -7.692(-8.009,-7.376) |  |
| Bulgaria | 18210.77(16623.62,19793.39) | 611.74(558.42,664.90) | 7718.55(6588.88,8841.14) | 406.32(346.85,465.42) | -57.62(-64.27,-50.07) | -1.86(-2.07,-1.65) | -6.417(-7.416,-5.418) |  |
| Burkina Faso | 8981.32(7192.33,10986.11) | 281.72(225.61,344.61) | 21588.15(16573.96,26608.47) | 249.44(191.51,307.45) | 140.37(79.67,215.15) | -0.30(-0.45,-0.14) | -0.893(-1.207,-0.579) |  |
| Burundi | 17669.78(13201.53,22538.00) | 852.38(636.83,1087.22) | 20857.47(15934.30,26074.39) | 395.61(302.23,494.56) | 18.04(-15.93,68.65) | -3.00(-3.42,-2.58) | -15.060(-15.344,-14.776) |  |
| Cabo Verde | 628.90(524.05,745.07) | 480.96(400.78,569.80) | 654.55(504.36,835.90) | 261.17(201.25,333.53) | 4.08(-18.26,32.76) | -2.15(-2.37,-1.93) | -7.591(-8.054,-7.127) |  |
| Cambodia | 22210.67(17727.46,26637.09) | 576.86(460.42,691.82) | 26778.23(20039.26,36913.25) | 369.70(276.67,509.63) | 20.56(-15.15,77.96) | -1.93(-2.13,-1.72) | -6.681(-6.892,-6.470) |  |
| Cameroon | 17011.98(13287.94,21339.85) | 447.30(349.38,561.09) | 51953.02(37862.98,67752.23) | 402.96(293.67,525.50) | 205.39(119.82,325.60) | -0.30(-0.67,0.06) | -1.409(-1.596,-1.222) |  |
| Canada | 17588.47(15740.01,19787.94) | 158.22(141.59,178.00) | 12815.61(11107.55,14603.42) | 108.04(93.64,123.12) | -27.14(-34.58,-19.98) | -1.48(-1.82,-1.14) | -1.432(-1.565,-1.300) |  |
| Central African Republic | 4958.86(3723.17,6410.59) | 476.20(357.54,615.61) | 8890.72(6142.08,12493.07) | 407.23(281.33,572.23) | 79.29(30.16,145.64) | -0.65(-0.75,-0.55) | -2.333(-2.752,-1.913) |  |
| Chad | 8521.68(6814.07,10580.33) | 405.97(324.62,504.05) | 24761.64(18224.58,32438.13) | 393.49(289.61,515.47) | 190.57(111.16,296.31) | -0.07(-0.28,0.14) | -0.318(-0.893,0.257) |  |
| Chile | 15690.62(14496.85,16897.57) | 273.96(253.12,295.03) | 10629.86(9489.16,11770.35) | 150.24(134.11,166.35) | -32.25(-38.35,-26.17) | -1.94(-2.04,-1.84) | -3.882(-4.147,-3.618) |  |
| China | 2738672.72(2394191.70,3090898.62) | 499.63(436.79,563.89) | 1654368.25(1394426.09,1906661.45) | 358.52(302.19,413.19) | -39.59(-48.93,-26.88) | -1.36(-1.55,-1.17) | -4.598(-4.981,-4.215) |  |
| Colombia | 44197.19(41413.38,46961.96) | 314.48(294.67,334.15) | 29884.68(25451.14,34699.47) | 148.72(126.66,172.68) | -32.38(-42.22,-21.58) | -2.40(-2.76,-2.03) | -5.319(-5.720,-4.919) |  |
| Comoros | 939.47(454.88,1278.82) | 543.86(263.33,740.31) | 960.89(741.79,1214.43) | 310.18(239.45,392.02) | 2.28(-31.21,111.18) | -2.54(-3.24,-1.84) | -7.945(-9.666,-6.225) |  |
| Congo | 4227.94(3010.77,5543.38) | 446.30(317.82,585.16) | 7023.04(5110.27,9786.58) | 316.98(230.65,441.71) | 66.11(13.50,145.03) | -1.35(-1.59,-1.11) | -3.355(-4.008,-2.702) |  |
| Cook Islands | 50.84(39.62,66.44) | 658.66(513.32,860.86) | 23.02(17.66,30.14) | 390.58(299.66,511.48) | -54.73(-69.05,-32.34) | -1.40(-1.69,-1.11) | -8.653(-9.142,-8.164) |  |
| Costa Rica | 2143.98(1957.33,2331.09) | 166.90(152.37,181.46) | 2330.23(2049.44,2591.12) | 122.43(107.68,136.14) | 8.69(-3.73,21.73) | -1.44(-1.78,-1.09) | -1.568(-1.818,-1.318) |  |
| Croatia | 5545.84(4945.21,6112.73) | 305.64(272.54,336.88) | 1257.90(1046.29,1485.32) | 100.78(83.82,119.00) | -77.32(-80.63,-73.44) | -3.80(-3.95,-3.65) | -6.597(-6.863,-6.332) |  |
| Cuba | 12980.25(12104.04,13826.46) | 266.01(248.05,283.35) | 5161.04(4486.62,5861.09) | 143.94(125.13,163.46) | -60.24(-65.28,-54.59) | -2.21(-2.42,-1.99) | -3.409(-3.872,-2.946) |  |
| Cyprus | 473.93(392.52,548.44) | 154.09(127.62,178.32) | 361.43(292.27,422.86) | 72.03(58.25,84.28) | -23.74(-39.00,-7.23) | -3.36(-3.82,-2.90) | -2.536(-2.698,-2.374) |  |
| Czechia | 9778.15(8687.88,10953.82) | 263.52(234.13,295.20) | 3382.60(2795.48,4040.67) | 114.55(94.66,136.83) | -65.41(-70.58,-59.53) | -2.38(-2.57,-2.19) | -4.877(-5.073,-4.681) |  |
| Côte d'Ivoire | 22412.03(17701.89,28632.55) | 473.82(374.24,605.33) | 46368.48(34709.60,59809.73) | 413.57(309.58,533.46) | 106.89(48.74,186.01) | -0.32(-0.49,-0.15) | -1.983(-2.258,-1.708) |  |
| Democratic People's Republic of Korea | 49684.20(36895.36,67736.50) | 595.67(442.35,812.11) | 69621.32(51166.43,99402.47) | 692.17(508.70,988.26) | 40.13(-3.48,110.59) | 0.17(-0.04,0.38) | 3.199(3.083,3.314) |  |
| Democratic Republic of the Congo | 45430.64(34031.16,58761.28) | 317.14(237.56,410.19) | 85837.97(61897.53,113729.75) | 237.87(171.53,315.16) | 88.94(44.50,158.35) | -1.01(-1.09,-0.94) | -2.739(-2.934,-2.544) |  |
| Denmark | 3642.97(3363.81,3926.86) | 190.94(176.31,205.82) | 1201.08(1033.09,1364.24) | 65.86(56.65,74.81) | -67.03(-70.78,-63.03) | -3.80(-4.07,-3.52) | -4.169(-4.460,-3.878) |  |
| Djibouti | 684.08(492.11,901.88) | 390.18(280.69,514.41) | 1597.13(1111.87,2266.85) | 295.35(205.61,419.19) | 133.47(55.37,245.91) | -1.08(-1.29,-0.86) | -2.674(-3.554,-1.794) |  |
| Dominica | 58.10(50.77,67.21) | 198.95(173.83,230.14) | 48.40(38.58,59.72) | 186.89(148.95,230.60) | -16.70(-36.20,8.24) | -0.42(-0.63,-0.22) | -0.414(-0.605,-0.223) |  |
| Dominican Republic | 12193.35(10486.82,14101.29) | 396.92(341.37,459.03) | 15572.07(12576.79,19713.36) | 342.45(276.58,433.52) | 27.71(-3.28,68.76) | 0.15(-0.19,0.49) | -2.256(-3.435,-1.078) |  |
| Ecuador | 20865.16(19582.86,22076.40) | 505.66(474.58,535.01) | 16639.85(13656.89,20000.60) | 227.80(186.97,273.81) | -20.25(-34.49,-2.98) | -2.70(-3.09,-2.31) | -8.937(-9.855,-8.019) |  |
| Egypt | 152198.79(123948.43,179878.45) | 694.26(565.40,820.52) | 157923.11(124203.33,195714.67) | 374.12(294.24,463.65) | 3.76(-18.73,33.97) | -1.67(-2.00,-1.35) | -10.370(-11.214,-9.526) |  |
| El Salvador | 8811.76(7743.03,9927.58) | 421.75(370.60,475.16) | 4899.69(3876.18,5924.41) | 189.03(149.55,228.57) | -44.40(-56.67,-30.60) | -2.52(-3.05,-1.99) | -7.630(-8.761,-6.499) |  |
| Equatorial Guinea | 692.18(512.84,943.21) | 458.39(339.62,624.63) | 1393.42(917.53,2072.19) | 200.32(131.90,297.90) | 101.31(33.70,212.89) | -3.22(-3.60,-2.84) | -8.085(-8.460,-7.710) |  |
| Eritrea | 9120.74(6927.98,11681.58) | 703.82(534.61,901.43) | 12720.18(8852.81,17505.89) | 454.30(316.18,625.22) | 39.46(-5.34,94.65) | -1.51(-1.63,-1.38) | -7.575(-8.320,-6.830) |  |
| Estonia | 1407.55(1225.51,1610.44) | 247.81(215.76,283.54) | 382.14(317.18,454.50) | 96.63(80.21,114.93) | -72.85(-77.90,-67.12) | -4.28(-4.71,-3.85) | -5.126(-5.421,-4.832) |  |
| Eswatini | 836.59(588.18,1087.02) | 277.60(195.17,360.70) | 1769.11(1158.33,2518.87) | 347.48(227.51,494.74) | 111.47(29.45,244.44) | 1.20(0.45,1.95) | 2.369(2.057,2.680) |  |
| Ethiopia | 97047.73(80218.51,133124.15) | 531.20(439.08,728.66) | 101600.76(81956.17,122144.12) | 219.18(176.80,263.50) | 4.69(-33.05,39.82) | -3.35(-3.57,-3.13) | -10.177(-10.298,-10.056) |  |
| Fiji | 2833.72(2392.74,3359.80) | 878.79(742.04,1041.94) | 2389.93(1879.48,3010.84) | 669.95(526.86,844.00) | -15.66(-35.55,11.22) | -0.96(-1.13,-0.79) | -6.563(-7.189,-5.937) |  |
| Finland | 5167.27(4711.60,5591.03) | 284.63(259.53,307.97) | 1657.69(1451.44,1878.74) | 99.50(87.12,112.77) | -67.92(-71.60,-63.89) | -3.32(-3.61,-3.04) | -6.018(-6.339,-5.696) |  |
| France | 34994.65(31993.85,37776.10) | 159.06(145.42,171.70) | 14051.46(12439.28,15795.36) | 70.73(62.61,79.51) | -59.85(-63.79,-55.74) | -2.76(-2.89,-2.63) | -2.834(-2.983,-2.686) |  |
| Gabon | 1217.08(956.01,1531.75) | 316.43(248.55,398.24) | 1552.86(1086.33,2176.65) | 207.17(144.93,290.39) | 27.59(-9.68,88.15) | -1.59(-1.76,-1.42) | -3.352(-3.691,-3.014) |  |
| Gambia | 1769.00(1378.06,2239.29) | 469.19(365.50,593.92) | 4445.75(3339.45,5861.12) | 444.51(333.90,586.03) | 151.31(73.56,263.95) | -0.45(-0.71,-0.19) | -1.092(-2.167,-0.018) |  |
| Georgia | 11869.79(10142.09,13788.62) | 557.54(476.39,647.67) | 3735.28(3090.29,4376.61) | 329.19(272.35,385.71) | -68.53(-74.53,-61.06) | -2.94(-3.95,-1.92) | -6.983(-10.789,-3.177) |  |
| Germany | 77844.39(72363.17,84425.29) | 262.03(243.58,284.18) | 21750.93(18717.38,24975.42) | 85.98(73.99,98.72) | -72.06(-75.28,-68.71) | -3.61(-3.87,-3.34) | -5.667(-5.816,-5.518) |  |
| Ghana | 44545.50(35654.12,54716.78) | 776.03(621.13,953.23) | 76099.54(58685.95,96423.63) | 532.10(410.34,674.21) | 70.84(23.82,138.97) | -1.11(-1.25,-0.97) | -7.810(-8.073,-7.548) |  |
| Greece | 10559.32(9770.96,11418.51) | 280.88(259.91,303.74) | 3648.41(3255.84,4069.90) | 131.06(116.96,146.20) | -65.45(-69.16,-61.53) | -2.50(-2.64,-2.35) | -5.050(-5.222,-4.878) |  |
| Greenland | 154.47(126.88,188.07) | 583.62(479.36,710.56) | 46.59(34.76,57.66) | 228.39(170.40,282.66) | -69.84(-77.41,-60.85) | -3.92(-4.29,-3.55) | -11.434(-11.759,-11.108) |  |
| Grenada | 190.46(172.60,209.07) | 571.25(517.69,627.06) | 81.89(69.69,94.35) | 202.59(172.41,233.43) | -57.01(-64.09,-49.14) | -3.21(-3.53,-2.90) | -12.890(-13.559,-12.221) |  |
| Guam | 256.28(212.39,295.05) | 404.05(334.86,465.17) | 236.80(205.78,273.21) | 427.14(371.20,492.83) | -7.60(-22.21,17.00) | 0.24(0.00,0.48) | 0.592(-0.432,1.617) |  |
| Guatemala | 12150.29(11386.66,12956.26) | 411.32(385.47,438.61) | 15818.74(13725.12,18139.46) | 232.40(201.64,266.49) | 30.19(11.55,50.49) | -2.66(-3.15,-2.17) | -4.710(-5.897,-3.524) |  |
| Guinea | 9695.84(7885.39,12279.57) | 471.95(383.83,597.72) | 22850.15(16967.93,29849.68) | 442.23(328.39,577.69) | 135.67(68.19,227.71) | 0.03(-0.06,0.13) | -1.021(-1.317,-0.724) |  |
| Guinea-Bissau | 3360.74(2535.03,4379.70) | 906.25(683.59,1181.02) | 5778.22(4375.86,7427.43) | 684.80(518.60,880.25) | 71.93(23.94,141.61) | -0.85(-0.88,-0.83) | -7.108(-7.338,-6.878) |  |
| Guyana | 2225.97(1873.04,2538.51) | 654.00(550.31,745.83) | 1358.79(1054.79,1758.85) | 437.28(339.45,566.03) | -38.96(-54.44,-20.05) | -1.45(-1.76,-1.13) | -6.647(-8.363,-4.931) |  |
| Haiti | 25037.31(19229.26,31851.26) | 1028.53(789.94,1308.45) | 37718.72(26769.40,51767.95) | 687.12(487.66,943.06) | 50.65(6.03,108.36) | -1.00(-1.20,-0.79) | -9.117(-10.152,-8.083) |  |
| Honduras | 11572.66(9539.59,13701.28) | 670.32(552.55,793.61) | 12972.24(7712.91,18973.21) | 295.12(175.47,431.64) | 12.09(-37.49,66.90) | -3.20(-3.53,-2.88) | -12.295(-12.849,-11.740) |  |
| Hungary | 17850.67(16174.48,19519.69) | 482.98(437.63,528.14) | 3832.19(3189.95,4493.94) | 139.28(115.93,163.33) | -78.53(-81.80,-74.97) | -4.24(-4.58,-3.89) | -10.977(-11.454,-10.499) |  |
| Iceland | 179.83(163.93,197.04) | 173.11(157.80,189.68) | 86.54(74.06,100.13) | 72.32(61.89,83.67) | -51.88(-57.43,-46.05) | -2.65(-2.88,-2.43) | -3.147(-3.251,-3.043) |  |
| India | 940272.37(816827.30,1067926.43) | 275.72(239.52,313.15) | 1149634.35(1015161.12,1283538.30) | 188.63(166.57,210.60) | 22.27(2.90,41.96) | -1.31(-1.54,-1.07) | -3.174(-3.476,-2.871) |  |
| Indonesia | 657599.70(575473.86,746500.26) | 842.60(737.37,956.51) | 725905.81(613719.40,921080.74) | 637.46(538.94,808.86) | 10.39(-9.65,45.68) | -0.83(-1.04,-0.62) | -6.768(-6.918,-6.617) |  |
| Iran (Islamic Republic of) | 69233.73(61372.09,77894.07) | 318.78(282.58,358.66) | 79658.18(71527.94,87469.11) | 229.54(206.11,252.05) | 15.06(1.82,30.98) | -0.91(-1.06,-0.76) | -2.889(-3.004,-2.775) |  |
| Iraq | 50639.87(42014.63,61092.44) | 704.78(584.74,850.26) | 69260.18(55005.67,89982.86) | 397.21(315.46,516.06) | 36.77(5.54,87.25) | -1.97(-2.24,-1.71) | -10.380(-10.851,-9.908) |  |
| Ireland | 2351.85(2177.26,2536.38) | 171.45(158.72,184.90) | 956.81(802.17,1118.44) | 61.15(51.27,71.48) | -59.32(-64.40,-54.19) | -3.32(-3.56,-3.09) | -3.480(-3.736,-3.224) |  |
| Israel | 2868.69(2604.19,3126.87) | 150.12(136.28,163.63) | 1702.10(1414.89,1992.68) | 51.22(42.58,59.97) | -40.67(-48.50,-31.85) | -3.63(-3.82,-3.44) | -3.165(-3.325,-3.005) |  |
| Italy | 40266.83(37619.61,43070.94) | 188.62(176.22,201.75) | 12539.86(11033.05,14101.03) | 79.39(69.85,89.28) | -68.86(-71.19,-66.53) | -2.99(-3.12,-2.86) | -3.548(-3.723,-3.372) |  |
| Jamaica | 3171.41(2847.08,3545.33) | 322.70(289.70,360.75) | 2526.07(1945.77,3250.87) | 211.70(163.07,272.44) | -20.35(-40.56,5.45) | -2.07(-2.73,-1.41) | -3.398(-4.295,-2.501) |  |
| Japan | 94863.22(87573.93,102246.98) | 211.68(195.41,228.16) | 46600.18(41586.23,51966.05) | 143.78(128.31,160.34) | -50.88(-53.31,-48.47) | -1.15(-1.35,-0.96) | -2.185(-2.390,-1.981) |  |
| Jordan | 6846.53(5773.90,8168.43) | 445.46(375.67,531.47) | 10718.09(8965.37,12530.82) | 199.66(167.01,233.43) | 56.55(21.44,95.70) | -3.11(-3.38,-2.85) | -7.919(-8.357,-7.480) |  |
| Kazakhstan | 31653.43(28614.19,34718.40) | 466.25(421.48,511.39) | 20039.34(15923.22,23932.12) | 287.53(228.47,343.39) | -36.69(-48.56,-25.42) | -2.49(-3.18,-1.79) | -5.824(-6.766,-4.882) |  |
| Kenya | 22096.55(17952.55,26006.70) | 252.31(204.99,296.96) | 48153.35(37982.03,60895.11) | 222.40(175.42,281.25) | 117.92(78.69,172.13) | -0.14(-0.41,0.13) | -1.046(-1.109,-0.983) |  |
| Kiribati | 406.49(327.21,494.17) | 1331.38(1071.70,1618.55) | 708.18(545.74,908.39) | 1424.24(1097.55,1826.89) | 74.22(27.09,140.02) | -0.03(-0.17,0.10) | 2.940(2.668,3.213) |  |
| Kuwait | 2052.05(1819.22,2281.59) | 242.81(215.26,269.98) | 3720.80(3105.85,4367.86) | 175.28(146.31,205.77) | 81.32(57.27,108.92) | -1.45(-2.08,-0.82) | -1.595(-2.054,-1.135) |  |
| Kyrgyzstan | 9708.88(8514.81,11039.57) | 538.30(472.09,612.08) | 7981.17(6606.90,9653.20) | 293.26(242.76,354.70) | -17.80(-33.61,2.12) | -3.66(-4.21,-3.10) | -7.709(-8.729,-6.689) |  |
| Lao People's Democratic Republic | 15386.94(11917.83,19585.07) | 995.91(771.37,1267.63) | 20890.78(15260.29,28155.13) | 651.24(475.72,877.69) | 35.77(-9.34,96.24) | -1.63(-1.73,-1.53) | -11.242(-11.475,-11.008) |  |
| Latvia | 2769.20(2507.71,3064.04) | 290.28(262.87,321.19) | 822.37(681.38,972.73) | 152.68(126.51,180.60) | -70.30(-74.71,-65.08) | -3.26(-3.69,-2.83) | -4.043(-4.707,-3.378) |  |
| Lebanon | 6087.13(4728.69,7974.24) | 528.04(410.20,691.74) | 5077.76(4269.85,6027.07) | 218.84(184.02,259.75) | -16.58(-39.51,14.58) | -2.54(-3.03,-2.04) | -9.685(-9.999,-9.371) |  |
| Lesotho | 1071.97(690.05,1454.16) | 198.67(127.89,269.50) | 3290.70(2336.24,4453.01) | 395.60(280.86,535.33) | 206.98(103.86,419.23) | 3.39(2.83,3.94) | 6.461(6.132,6.790) |  |
| Liberia | 4351.51(3406.58,5578.85) | 471.66(369.24,604.70) | 9567.25(7199.11,12998.06) | 426.12(320.65,578.93) | 119.86(56.26,216.05) | -0.20(-0.43,0.03) | -1.610(-1.984,-1.237) |  |
| Libya | 7034.56(5555.95,8876.34) | 418.67(330.67,528.29) | 13134.62(9908.52,17386.31) | 437.74(330.23,579.44) | 86.72(43.32,146.07) | 0.53(0.27,0.80) | 1.279(0.834,1.724) |  |
| Lithuania | 3336.78(2909.14,3806.17) | 239.48(208.79,273.17) | 1026.31(842.12,1219.93) | 127.48(104.60,151.53) | -69.24(-73.52,-64.13) | -2.24(-2.66,-1.82) | -3.720(-4.295,-3.146) |  |
| Luxembourg | 392.38(360.99,421.61) | 265.88(244.62,285.69) | 127.38(107.03,148.83) | 57.74(48.52,67.46) | -67.54(-72.14,-62.12) | -5.64(-5.89,-5.38) | -6.910(-7.114,-6.706) |  |
| Madagascar | 50511.12(42133.70,59068.58) | 1115.13(930.18,1304.05) | 94941.29(67986.09,123016.44) | 810.72(580.54,1050.46) | 87.96(30.84,155.84) | -1.03(-1.12,-0.93) | -10.209(-10.943,-9.475) |  |
| Malawi | 15877.07(12835.43,19656.30) | 424.92(343.52,526.06) | 31886.38(24794.85,39682.27) | 389.66(303.00,484.92) | 100.83(50.46,172.08) | -0.44(-0.66,-0.21) | -0.977(-1.287,-0.667) |  |
| Malaysia | 35470.95(31424.44,40285.78) | 477.92(423.40,542.79) | 45890.72(40097.59,52550.90) | 330.09(288.42,378.00) | 29.38(9.96,54.75) | -1.28(-1.62,-0.94) | -5.056(-5.720,-4.392) |  |
| Maldives | 669.93(563.47,856.61) | 824.37(693.37,1054.07) | 860.82(693.86,1028.72) | 330.88(266.70,395.41) | 28.49(-4.76,62.34) | -3.29(-3.71,-2.86) | -15.981(-17.255,-14.708) |  |
| Mali | 16366.67(12941.90,20403.92) | 548.17(433.47,683.39) | 34014.67(25715.59,43202.98) | 381.88(288.71,485.04) | 107.83(55.57,179.87) | -1.11(-1.23,-0.98) | -5.221(-5.507,-4.934) |  |
| Malta | 263.95(240.70,288.69) | 191.28(174.43,209.20) | 107.75(92.66,123.56) | 80.50(69.22,92.31) | -59.18(-64.10,-54.28) | -2.65(-2.81,-2.48) | -3.517(-3.776,-3.257) |  |
| Marshall Islands | 219.16(177.90,267.78) | 1277.68(1037.13,1561.14) | 332.12(246.95,439.15) | 1400.08(1041.04,1851.28) | 51.54(11.66,103.20) | 0.52(0.38,0.66) | 3.228(2.408,4.048) |  |
| Mauritania | 4054.09(3239.32,5083.71) | 527.31(421.33,661.23) | 4572.01(3267.73,6515.01) | 267.83(191.43,381.66) | 12.78(-17.21,51.71) | -2.28(-2.37,-2.19) | -8.482(-8.769,-8.194) |  |
| Mauritius | 2865.75(2627.38,3111.99) | 576.47(528.52,626.01) | 2169.14(1950.96,2363.14) | 476.52(428.59,519.13) | -24.31(-32.15,-15.67) | 0.47(0.11,0.83) | -0.356(-1.872,1.161) |  |
| Mexico | 82382.01(78650.35,86356.61) | 231.01(220.54,242.15) | 91695.42(83110.99,100897.17) | 178.01(161.34,195.87) | 11.31(1.68,21.08) | -0.77(-1.05,-0.49) | -1.631(-1.906,-1.356) |  |
| Micronesia (Federated States of) | 587.91(432.99,735.54) | 1467.37(1080.71,1835.82) | 538.11(408.67,692.97) | 1267.16(962.35,1631.82) | -8.47(-32.80,27.76) | -0.41(-0.46,-0.37) | -6.297(-6.484,-6.110) |  |
| Monaco | 23.19(18.80,28.63) | 253.43(205.52,312.89) | 12.96(9.25,18.33) | 138.98(99.27,196.67) | -44.13(-61.68,-19.43) | -2.10(-2.26,-1.93) | -3.696(-3.804,-3.587) |  |
| Mongolia | 3826.50(3073.77,4833.63) | 433.02(347.84,546.99) | 4805.62(3651.51,6114.52) | 380.75(289.31,484.46) | 25.59(-7.84,66.85) | -0.87(-1.29,-0.44) | -3.006(-4.218,-1.794) |  |
| Montenegro | 1191.00(1005.28,1412.95) | 474.45(400.47,562.87) | 542.23(436.15,665.81) | 263.57(212.00,323.64) | -54.47(-64.36,-41.55) | -2.18(-2.65,-1.71) | -6.485(-7.610,-5.360) |  |
| Morocco | 57508.76(42341.40,77199.37) | 553.14(407.26,742.54) | 45397.54(32205.37,66709.01) | 309.22(219.36,454.38) | -21.06(-42.69,18.72) | -2.03(-2.23,-1.83) | -7.893(-8.131,-7.656) |  |
| Mozambique | 17640.47(14140.58,21740.54) | 371.92(298.13,458.36) | 56962.58(41117.99,75292.67) | 473.76(341.98,626.21) | 222.91(126.57,347.39) | 1.50(1.22,1.79) | 3.383(3.138,3.629) |  |
| Myanmar | 195931.34(154732.12,252400.54) | 1141.30(901.31,1470.23) | 155005.07(120568.55,202713.86) | 689.47(536.30,901.68) | -20.89(-42.03,13.05) | -1.81(-2.06,-1.56) | -14.862(-15.289,-14.436) |  |
| Namibia | 1527.84(1024.65,1989.35) | 273.21(183.23,355.74) | 2309.16(1469.16,3486.89) | 220.96(140.58,333.65) | 51.14(4.29,123.34) | -1.04(-1.47,-0.61) | -1.668(-1.960,-1.376) |  |
| Nauru | 77.37(56.95,109.47) | 1913.67(1408.48,2707.50) | 88.77(65.68,124.39) | 1907.37(1411.15,2672.63) | 14.73(-14.71,57.56) | -0.13(-0.57,0.32) | 0.523(-0.657,1.702) |  |
| Nepal | 21816.16(16415.40,29376.82) | 298.64(224.71,402.14) | 24279.27(18104.96,32649.24) | 181.09(135.03,243.51) | 11.29(-22.04,58.53) | -1.66(-1.75,-1.56) | -3.911(-4.014,-3.809) |  |
| Netherlands | 10547.01(9563.80,11603.17) | 174.92(158.61,192.43) | 3478.02(2921.65,4047.81) | 65.87(55.33,76.66) | -67.02(-70.50,-62.76) | -3.69(-3.98,-3.39) | -3.645(-3.804,-3.486) |  |
| New Zealand | 2300.74(2107.91,2495.84) | 166.54(152.58,180.67) | 1406.75(1212.09,1625.52) | 78.09(67.29,90.24) | -38.86(-45.11,-32.45) | -2.89(-3.07,-2.71) | -2.886(-3.162,-2.609) |  |
| Nicaragua | 3691.10(3268.52,4183.61) | 250.07(221.44,283.43) | 4169.32(3485.72,5008.28) | 146.49(122.47,175.97) | 12.96(-8.20,40.99) | -1.72(-1.94,-1.50) | -3.519(-3.933,-3.105) |  |
| Niger | 10571.33(8104.96,13996.03) | 379.81(291.19,502.85) | 24552.08(18060.19,31749.37) | 275.33(202.53,356.04) | 132.25(76.45,208.68) | -1.10(-1.23,-0.98) | -3.330(-3.486,-3.174) |  |
| Nigeria | 94751.95(77362.47,116532.18) | 277.51(226.58,341.29) | 165306.26(130553.16,211725.93) | 183.80(145.16,235.41) | 74.46(31.91,135.79) | -1.57(-1.74,-1.41) | -3.032(-3.096,-2.968) |  |
| Niue | 6.89(5.21,9.64) | 855.77(646.73,1197.88) | 4.77(3.86,5.94) | 840.77(680.45,1046.78) | -30.69(-51.33,-4.05) | -0.89(-1.14,-0.65) | 0.148(-1.121,1.418) |  |
| North Macedonia | 4234.78(3650.55,4818.94) | 533.28(459.71,606.84) | 1988.79(1604.29,2464.28) | 260.05(209.78,322.23) | -53.04(-62.13,-41.19) | -2.46(-2.69,-2.23) | -9.002(-9.789,-8.215) |  |
| Northern Mariana Islands | 171.50(122.16,228.62) | 732.30(521.63,976.20) | 74.81(61.55,92.22) | 453.70(373.28,559.29) | -56.38(-68.75,-37.08) | -1.91(-2.08,-1.73) | -8.652(-9.875,-7.428) |  |
| Norway | 2479.88(2294.46,2696.33) | 155.01(143.42,168.54) | 966.91(786.95,1146.39) | 54.48(44.34,64.59) | -61.01(-65.66,-56.68) | -3.67(-3.83,-3.50) | -3.140(-3.289,-2.991) |  |
| Oman | 3372.53(2633.94,4251.69) | 406.48(317.46,512.44) | 5653.56(4584.16,6745.63) | 244.29(198.08,291.48) | 67.64(25.67,122.68) | -1.08(-1.31,-0.85) | -5.284(-5.707,-4.861) |  |
| Pakistan | 109287.77(84623.15,132018.39) | 267.83(207.38,323.54) | 327264.07(257353.12,413326.30) | 330.88(260.20,417.90) | 199.45(127.97,307.33) | 0.36(0.07,0.66) | 2.112(1.849,2.374) |  |
| Palau | 75.34(57.26,97.20) | 1080.06(820.91,1393.40) | 74.96(61.09,91.95) | 1272.70(1037.25,1561.16) | -0.51(-28.58,39.26) | 0.67(0.56,0.79) | 6.027(4.646,7.409) |  |
| Palestine | 3303.75(2567.23,4174.92) | 430.46(334.49,543.97) | 5151.78(4364.96,5918.50) | 235.93(199.89,271.04) | 55.94(20.03,108.77) | -1.95(-2.22,-1.67) | -6.611(-7.094,-6.129) |  |
| Panama | 2518.12(2334.17,2705.80) | 248.93(230.75,267.49) | 2802.78(2366.57,3290.99) | 169.82(143.39,199.40) | 11.30(-7.57,32.63) | -1.29(-1.56,-1.03) | -2.537(-2.786,-2.287) |  |
| Papua New Guinea | 10215.13(6750.19,15154.57) | 617.16(407.82,915.58) | 23557.46(16044.30,32596.15) | 550.47(374.91,761.68) | 130.61(51.37,264.94) | -0.53(-0.67,-0.40) | -2.057(-2.787,-1.327) |  |
| Paraguay | 5417.38(4670.85,6265.45) | 345.65(298.02,399.76) | 5648.55(4485.24,7157.71) | 184.62(146.60,233.95) | 4.27(-20.55,35.95) | -2.04(-2.19,-1.89) | -4.644(-5.087,-4.201) |  |
| Peru | 43564.92(37325.13,50821.25) | 491.08(420.74,572.87) | 42071.48(33150.53,51539.66) | 283.10(223.07,346.81) | -3.43(-28.16,24.82) | -1.74(-2.05,-1.43) | -5.412(-6.560,-4.263) |  |
| Philippines | 121364.21(111110.10,132460.59) | 468.24(428.68,511.05) | 250397.95(218491.02,284398.33) | 529.94(462.41,601.90) | 106.32(78.03,139.93) | 1.12(0.78,1.46) | 2.041(1.608,2.474) |  |
| Poland | 61554.25(57839.24,65040.81) | 426.13(400.41,450.27) | 20803.15(18579.05,23148.36) | 171.95(153.56,191.33) | -66.20(-69.04,-63.37) | -2.90(-3.21,-2.60) | -8.154(-8.420,-7.887) |  |
| Portugal | 15265.62(14332.52,16263.65) | 403.20(378.56,429.56) | 3155.94(2882.27,3467.17) | 106.92(97.65,117.47) | -79.33(-81.25,-77.28) | -4.79(-5.14,-4.44) | -9.426(-10.066,-8.786) |  |
| Puerto Rico | 2556.08(2357.76,2786.67) | 180.70(166.68,197.00) | 978.24(840.64,1118.53) | 94.58(81.28,108.15) | -61.73(-67.13,-56.36) | -2.61(-2.87,-2.36) | -2.664(-2.960,-2.368) |  |
| Qatar | 882.72(728.20,1047.97) | 373.32(307.97,443.21) | 2824.47(2285.46,3398.49) | 170.92(138.30,205.66) | 219.97(147.35,312.02) | -3.03(-3.34,-2.72) | -6.143(-6.750,-5.535) |  |
| Republic of Korea | 104643.56(90237.23,119526.11) | 497.16(428.72,567.87) | 23476.79(19998.28,27298.52) | 146.72(124.98,170.60) | -77.56(-81.42,-72.52) | -4.44(-4.74,-4.14) | -11.283(-11.575,-10.992) |  |
| Republic of Moldova | 6119.95(5425.56,6818.31) | 351.15(311.31,391.22) | 2517.60(2165.60,2932.11) | 202.99(174.61,236.41) | -58.86(-65.04,-52.14) | -2.35(-2.65,-2.05) | -4.472(-5.347,-3.597) |  |
| Romania | 33088.76(29960.17,36418.45) | 380.90(344.88,419.23) | 12812.00(11159.32,14795.72) | 237.73(207.07,274.54) | -61.28(-66.82,-55.13) | -1.60(-1.73,-1.47) | -4.446(-4.916,-3.976) |  |
| Russian Federation | 219188.14(204264.60,232959.04) | 376.80(351.14,400.47) | 182960.41(169125.34,197631.80) | 393.67(363.90,425.24) | -16.53(-22.48,-10.95) | -0.40(-0.80,-0.01) | 1.119(0.212,2.025) |  |
| Rwanda | 27793.03(21521.30,35630.87) | 1014.14(785.29,1300.13) | 16185.55(11335.14,21453.81) | 285.39(199.87,378.28) | -41.76(-61.62,-14.37) | -5.33(-5.94,-4.72) | -24.108(-25.005,-23.211) |  |
| Saint Kitts and Nevis | 124.88(115.48,135.60) | 722.65(668.26,784.66) | 42.13(32.08,55.47) | 185.38(141.16,244.08) | -66.26(-74.96,-55.87) | -5.09(-5.69,-4.48) | -17.430(-17.893,-16.968) |  |
| Saint Lucia | 217.46(202.26,234.43) | 386.52(359.51,416.70) | 138.06(116.28,161.35) | 208.89(175.92,244.12) | -36.51(-46.63,-24.90) | -2.37(-2.65,-2.08) | -6.426(-7.319,-5.533) |  |
| Saint Vincent and the Grenadines | 187.14(172.23,204.20) | 407.43(374.97,444.57) | 107.10(91.87,122.63) | 259.19(222.34,296.76) | -42.77(-51.57,-33.55) | -2.19(-2.47,-1.90) | -4.629(-5.255,-4.004) |  |
| Samoa | 475.45(364.01,622.59) | 710.01(543.59,929.73) | 613.35(454.37,805.82) | 763.37(565.50,1002.91) | 29.00(-8.18,79.27) | 0.25(0.11,0.39) | 1.670(1.347,1.994) |  |
| San Marino | 11.16(9.47,13.02) | 118.85(100.85,138.68) | 5.56(4.28,7.02) | 61.99(47.75,78.35) | -50.22(-63.10,-35.90) | -1.95(-2.10,-1.80) | -1.891(-1.960,-1.822) |  |
| Sao Tome and Principe | 176.42(120.49,229.95) | 410.98(280.69,535.67) | 376.13(264.40,529.80) | 413.84(290.91,582.91) | 113.20(30.60,258.11) | -0.32(-0.84,0.21) | -0.770(-1.921,0.381) |  |
| Saudi Arabia | 32484.62(24715.17,41752.55) | 488.84(371.92,628.31) | 81839.52(60776.89,109682.13) | 441.84(328.13,592.16) | 151.93(67.57,284.62) | -0.02(-0.22,0.18) | -1.416(-1.662,-1.170) |  |
| Senegal | 15057.22(12267.25,18298.43) | 545.66(444.55,663.12) | 22732.08(18202.86,29275.24) | 352.55(282.31,454.03) | 50.97(11.84,103.38) | -1.23(-1.38,-1.07) | -6.321(-7.500,-5.142) |  |
| Serbia | 17504.77(14880.99,20137.73) | 487.44(414.38,560.76) | 5298.75(4224.92,6391.01) | 178.75(142.53,215.60) | -69.73(-76.73,-61.72) | -3.45(-3.59,-3.32) | -9.842(-10.305,-9.379) |  |
| Seychelles | 166.17(145.83,187.19) | 532.28(467.12,599.61) | 124.94(106.11,144.76) | 325.50(276.43,377.13) | -24.81(-37.57,-10.87) | -1.29(-1.51,-1.07) | -5.998(-7.012,-4.984) |  |
| Sierra Leone | 7895.47(5956.26,10146.85) | 494.27(372.87,635.21) | 17381.29(12821.81,23386.22) | 466.02(343.77,627.02) | 120.14(62.33,200.11) | 0.12(-0.06,0.29) | -0.779(-1.227,-0.332) |  |
| Singapore | 3209.89(2902.27,3544.84) | 212.68(192.30,234.87) | 1940.85(1650.03,2255.75) | 100.89(85.77,117.26) | -39.54(-46.60,-32.16) | -2.78(-3.08,-2.48) | -3.582(-3.865,-3.300) |  |
| Slovakia | 6533.62(5534.32,7557.56) | 319.17(270.35,369.19) | 2615.27(2151.61,3094.51) | 152.82(125.73,180.83) | -59.97(-67.41,-50.91) | -2.10(-2.32,-1.88) | -5.295(-5.527,-5.063) |  |
| Slovenia | 1475.91(1282.91,1674.83) | 192.59(167.41,218.55) | 363.10(278.09,447.09) | 63.77(48.84,78.52) | -75.40(-80.01,-71.24) | -3.98(-4.16,-3.80) | -4.195(-4.367,-4.022) |  |
| Solomon Islands | 743.81(462.14,996.93) | 579.89(360.30,777.23) | 1889.33(1437.53,2401.41) | 690.75(525.57,877.97) | 154.01(74.73,310.29) | 0.73(0.64,0.82) | 3.669(3.362,3.976) |  |
| Somalia | 18110.53(13149.34,23841.62) | 624.66(453.54,822.34) | 33313.44(23793.51,45150.56) | 401.82(286.99,544.59) | 83.95(29.00,167.99) | -1.34(-1.60,-1.07) | -6.917(-7.149,-6.685) |  |
| South Africa | 88394.11(79816.54,96972.81) | 561.74(507.23,616.26) | 71816.10(64103.21,80060.98) | 296.19(264.38,330.19) | -18.75(-28.90,-7.71) | -2.64(-3.53,-1.73) | -10.129(-11.332,-8.927) |  |
| South Sudan | 9291.24(6874.95,12413.03) | 402.49(297.81,537.72) | 11907.46(8467.48,16650.42) | 330.85(235.27,462.63) | 28.16(-8.50,78.71) | -0.84(-1.31,-0.37) | -1.816(-2.307,-1.326) |  |
| Spain | 35881.82(33375.90,38685.99) | 241.96(225.06,260.87) | 9554.77(8458.62,10755.34) | 76.95(68.13,86.62) | -73.37(-75.96,-70.23) | -3.80(-3.96,-3.63) | -5.433(-5.635,-5.231) |  |
| Sri Lanka | 33438.16(28862.00,37864.67) | 452.20(390.31,512.06) | 22205.27(16642.50,28209.41) | 275.34(206.36,349.79) | -33.59(-51.90,-10.68) | -1.84(-2.11,-1.58) | -5.702(-6.703,-4.701) |  |
| Sudan | 59124.10(41098.11,79399.49) | 775.85(539.31,1041.91) | 81543.17(52059.42,114414.22) | 441.12(281.62,618.94) | 37.92(-12.29,114.54) | -1.82(-1.88,-1.75) | -10.884(-11.006,-10.762) |  |
| Suriname | 837.08(591.21,977.12) | 514.03(363.05,600.03) | 831.10(674.05,1034.80) | 387.18(314.02,482.08) | -0.71(-22.58,37.86) | -1.34(-1.68,-1.00) | -3.194(-4.517,-1.871) |  |
| Sweden | 4138.65(3749.01,4580.27) | 140.99(127.72,156.03) | 2139.67(1705.45,2623.25) | 66.01(52.61,80.93) | -48.30(-55.78,-40.39) | -2.36(-2.47,-2.25) | -2.404(-2.510,-2.298) |  |
| Switzerland | 4081.75(3746.23,4433.60) | 154.91(142.17,168.26) | 1319.85(1108.94,1564.83) | 47.54(39.94,56.36) | -67.66(-71.71,-63.03) | -4.40(-4.66,-4.14) | -3.583(-3.705,-3.462) |  |
| Syrian Arab Republic | 49675.91(39764.12,61090.91) | 1035.47(828.86,1273.40) | 23737.95(18560.44,30716.73) | 466.72(364.92,603.93) | -52.21(-65.77,-33.75) | -2.62(-2.94,-2.29) | -17.774(-18.924,-16.624) |  |
| Taiwan (Province of China) | 33532.85(30449.35,36549.33) | 363.44(330.02,396.13) | 16458.10(14089.41,18642.88) | 218.14(186.74,247.10) | -50.92(-55.64,-46.33) | -1.44(-1.65,-1.24) | -4.764(-5.555,-3.972) |  |
| Tajikistan | 9155.39(7620.39,11031.55) | 432.95(360.36,521.67) | 11172.60(8083.02,14484.32) | 267.85(193.78,347.25) | 22.03(-16.46,69.64) | -2.81(-3.33,-2.29) | -5.056(-5.484,-4.628) |  |
| Thailand | 99975.94(81534.68,119450.63) | 385.53(314.42,460.63) | 120209.84(94101.93,147989.99) | 567.04(443.88,698.08) | 20.24(-8.86,58.55) | 0.42(-0.24,1.09) | 5.268(3.886,6.650) |  |
| Timor-Leste | 1633.87(1279.99,2089.89) | 513.31(402.13,656.58) | 2605.04(1728.62,3526.46) | 455.95(302.56,617.23) | 59.44(1.77,122.43) | -0.40(-0.94,0.14) | -2.019(-2.845,-1.192) |  |
| Togo | 7122.28(5724.70,8679.48) | 519.54(417.59,633.13) | 14166.05(10275.77,18409.12) | 421.04(305.42,547.15) | 98.90(37.52,175.28) | -0.66(-0.80,-0.52) | -3.000(-3.209,-2.792) |  |
| Tokelau | 5.14(3.62,7.01) | 885.97(624.46,1209.12) | 4.65(3.78,5.49) | 939.36(763.56,1109.07) | -9.58(-33.19,28.61) | -0.55(-0.84,-0.26) | 3.531(2.025,5.038) |  |
| Tonga | 116.88(96.72,140.06) | 316.77(262.13,379.61) | 133.40(104.50,178.43) | 342.95(268.66,458.71) | 14.14(-15.77,58.33) | 0.42(0.33,0.52) | 0.800(0.666,0.934) |  |
| Trinidad and Tobago | 1904.41(1780.60,2044.70) | 379.86(355.16,407.84) | 1581.69(1247.57,1985.45) | 317.75(250.63,398.86) | -16.95(-34.50,6.13) | -1.07(-1.42,-0.71) | -1.827(-2.615,-1.038) |  |
| Tunisia | 10240.45(7990.04,12974.12) | 297.70(232.28,377.18) | 10098.51(7458.75,13193.49) | 232.06(171.40,303.18) | -1.39(-23.45,25.90) | -0.96(-1.02,-0.90) | -2.048(-2.213,-1.884) |  |
| Turkey | 128220.02(106936.24,156532.51) | 536.06(447.08,654.43) | 65056.27(53877.68,76422.27) | 204.20(169.11,239.87) | -49.26(-60.12,-37.12) | -3.30(-3.54,-3.06) | -10.481(-10.710,-10.251) |  |
| Turkmenistan | 6185.39(5412.16,7034.55) | 402.94(352.57,458.26) | 12943.99(10461.16,15793.60) | 622.35(502.97,759.36) | 109.27(61.79,163.33) | 1.15(0.65,1.65) | 9.040(6.294,11.785) |  |
| Tuvalu | 51.52(40.36,65.72) | 1427.67(1118.29,1821.00) | 52.77(41.64,64.48) | 1061.97(838.06,1297.72) | 2.41(-23.47,34.36) | -1.00(-1.09,-0.91) | -11.573(-11.918,-11.229) |  |
| Uganda | 22951.49(17120.85,29756.71) | 357.82(266.92,463.91) | 45549.79(33990.89,60445.54) | 264.85(197.64,351.46) | 98.46(32.52,188.19) | -1.99(-2.40,-1.58) | -3.244(-3.508,-2.981) |  |
| Ukraine | 54891.67(47737.43,61764.45) | 288.99(251.32,325.17) | 51936.34(39166.12,64962.81) | 376.76(284.12,471.26) | -5.38(-27.41,22.55) | 0.27(-0.02,0.56) | 2.730(0.939,4.520) |  |
| United Arab Emirates | 3367.55(2705.82,4277.23) | 352.29(283.06,447.45) | 8616.51(6888.31,10584.51) | 214.48(171.46,263.47) | 155.87(102.30,227.85) | -1.67(-1.86,-1.47) | -4.412(-5.083,-3.740) |  |
| United Kingdom | 38141.58(35644.23,40715.33) | 182.50(170.55,194.81) | 17941.00(15892.22,19931.69) | 82.48(73.06,91.63) | -52.96(-55.52,-50.42) | -2.76(-2.95,-2.56) | -3.443(-3.581,-3.304) |  |
| United Republic of Tanzania | 37002.39(29592.31,45432.10) | 381.90(305.42,468.91) | 62731.71(46818.64,82414.05) | 268.85(200.65,353.20) | 69.53(20.33,135.48) | -1.43(-1.57,-1.28) | -3.612(-3.772,-3.452) |  |
| United States of America | 203158.54(183975.69,223337.87) | 198.84(180.07,218.59) | 159717.44(140763.72,181109.89) | 143.50(126.47,162.72) | -21.38(-25.63,-17.26) | -1.12(-1.22,-1.03) | -1.777(-1.855,-1.698) |  |
| United States Virgin Islands | 122.59(98.42,150.12) | 309.48(248.45,378.96) | 50.01(33.16,68.62) | 216.23(143.39,296.72) | -59.21(-71.84,-43.21) | -1.12(-1.42,-0.82) | -2.468(-3.215,-1.721) |  |
| Uruguay | 5446.81(5119.58,5782.20) | 479.55(450.74,509.08) | 2432.23(2247.45,2633.14) | 203.32(187.88,220.12) | -55.35(-58.82,-51.56) | -2.90(-3.20,-2.60) | -9.007(-9.485,-8.530) |  |
| Uzbekistan | 39245.41(35971.29,42597.02) | 457.22(419.08,496.27) | 38877.25(33415.75,44463.06) | 283.01(243.25,323.67) | -0.94(-15.32,14.65) | -1.81(-2.42,-1.18) | -5.827(-6.734,-4.920) |  |
| Vanuatu | 658.88(485.11,874.72) | 1124.73(828.09,1493.17) | 1429.87(1059.44,1802.98) | 1147.21(850.01,1446.56) | 117.01(44.89,212.34) | -0.18(-0.28,-0.09) | -0.243(-1.091,0.605) |  |
| Venezuela (Bolivarian Republic of) | 26267.70(24250.03,28199.61) | 327.97(302.78,352.09) | 26586.30(19992.58,33818.63) | 283.96(213.53,361.21) | 1.21(-23.34,30.68) | -0.86(-1.33,-0.39) | -0.925(-1.368,-0.483) |  |
| Viet Nam | 134608.52(103761.65,169479.77) | 472.02(363.85,594.30) | 152123.45(117778.23,202520.54) | 396.26(306.79,527.53) | 13.01(-21.46,60.39) | -0.59(-0.82,-0.35) | -2.628(-2.748,-2.508) |  |
| Yemen | 23344.90(13682.73,32876.48) | 507.84(297.65,715.19) | 51118.57(34248.70,72367.48) | 371.51(248.91,525.94) | 118.97(51.35,228.25) | -1.04(-1.21,-0.86) | -4.163(-4.904,-3.422) |  |
| Zambia | 14578.60(11759.38,18034.85) | 480.47(387.55,594.37) | 29102.92(19740.00,40747.11) | 359.61(243.92,503.49) | 99.63(30.99,180.77) | -1.28(-1.51,-1.05) | -4.161(-4.395,-3.926) |  |
| Zimbabwe | 6348.69(5231.59,7637.49) | 160.13(131.96,192.64) | 25432.56(18762.77,33591.55) | 401.29(296.05,530.03) | 300.60(184.06,469.88) | 3.97(2.99,4.96) | 7.724(7.289,8.158) |  |

Note: EAPC, estimated annual percentage change; AAPC, average annual percentage change; CI, confidence interval; GBD, Global Burden of Disease; SDI, socio-demographic
